# A cAMP signalosome in primary cilia drives gene expression and kidney cyst formation

**DOI:** 10.15252/embr.202154315

**Published:** 2022-06-13

**Authors:** Jan N Hansen, Fabian Kaiser, Philipp Leyendecker, Birthe Stüven, Jens‐Henning Krause, Fatemeh Derakhshandeh, Jaazba Irfan, Tommy J Sroka, Kenley M Preval, Paurav B Desai, Michael Kraut, Heidi Theis, Anna‐Dorothee Drews, Elena De‐Domenico, Kristian Händler, Gregory J Pazour, David J P Henderson, David U Mick, Dagmar Wachten

**Affiliations:** ^1^ Institute of Innate Immunity Medical Faculty University of Bonn Bonn Germany; ^2^ Mironid Ltd. SIPBS Glasgow Scotland, UK; ^3^ Center for Molecular Signaling (PZMS) Center of Human and Molecular Biology (ZHMB) Saarland University, School of Medicine Homburg Germany; ^4^ Program in Molecular Medicine University of Massachusetts Chan Medical School, Biotech II Worcester MA USA; ^5^ Precise Platform for Single Cell Genomics and Epigenomics Department of Systems Medicine German Center for Neurogenerative Diseases Bonn Germany

**Keywords:** cAMP, CREB, optogenetics, PKD, primary cilia, Cell Adhesion, Polarity & Cytoskeleton, Signal Transduction

## Abstract

The primary cilium constitutes an organelle that orchestrates signal transduction independently from the cell body. Dysregulation of this intricate molecular architecture leads to severe human diseases, commonly referred to as ciliopathies. However, the molecular underpinnings how ciliary signaling orchestrates a specific cellular output remain elusive. By combining spatially resolved optogenetics with RNA sequencing and imaging, we reveal a novel cAMP signalosome that is functionally distinct from the cytoplasm. We identify the genes and pathways targeted by the ciliary cAMP signalosome and shed light on the underlying mechanisms and downstream signaling. We reveal that chronic stimulation of the ciliary cAMP signalosome transforms kidney epithelia from tubules into cysts. Counteracting this chronic cAMP elevation in the cilium by small molecules targeting activation of phosphodiesterase‐4 long isoforms inhibits cyst growth. Thereby, we identify a novel concept of how the primary cilium controls cellular functions and maintains tissue integrity in a specific and spatially distinct manner and reveal novel molecular components that might be involved in the development of one of the most common genetic diseases, polycystic kidney disease.

## Graphical abstract

This study reveals a cAMP signalosome in primary cilia that evokes a distinct gene expression program *via* PKA‐dependent CREB phosphorylation in the cilium. Chronic stimulation of this pathway remodels renal epithelial cells and drives cystogenesis.

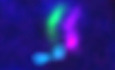

## Introduction

Primary cilia are microtubule‐based protrusions of the plasma membrane, which are present on most vertebrate cells. The primary cilium receives environmental stimuli and transduces this information into an intracellular response. In turn, ciliary signaling controls cellular functions, such as cell proliferation or differentiation (Pazour & Witman, 2003; Anvarian *et al*, 2019; Nachury & Mick, 2019; Wachten & Mick, 2021). Signal transduction in the primary cilium is temporally and spatially compartmentalized. This is maintained by (i) the transition zone, which prevents lateral protein diffusion, (ii) the intraflagellar transport (IFT) machinery, which provides anterograde and retrograde protein transport along the axoneme in and out of the cilium, and (iii) the BBSome, which is part of the IFT (Nachury, 2018). Thereby, ciliary signaling controls cellular functions, such as cell proliferation or differentiation, independently of the rest of the cell (Pazour & Witman, 2003; Anvarian *et al*, 2019; Nachury & Mick, 2019; Truong *et al*, 2021; Wachten & Mick, 2021). Highlighting the relevance of primary cilia for tissue and organ function, primary cilia dysfunction leads to severe diseases commonly referred to as ciliopathies with a broad band of phenotypes (Hildebrandt *et al*, 2011; Pazour *et al*, 2020; Richards *et al*, 2021). However, how ciliary signals are transduced into a cellular response and the molecular mechanisms underlying disease development are not well understood.

Cyclic AMP (cAMP) signaling is an important second messenger for ciliary signaling. Components of the cAMP signaling cascade are enriched in primary cilia (Mick *et al*, 2015; Bachmann *et al*, 2016; Hilgendorf *et al*, 2016; Mykytyn & Askwith, 2017; Wachten & Mick, 2021). Remarkably, adenylyl cyclase 3 (AC3) is commonly used as a primary cilia marker in different cell types (Bishop *et al*, 2007). Recent reports demonstrating that mislocalization of ciliary cAMP signaling is associated with ciliopathies and their symptoms further substantiate the central role of cAMP in ciliary signaling (Siljee *et al*, 2018; Somatilaka *et al*, 2020; Wang *et al*, 2021).

This applies to the most common ciliopathy, autosomal‐dominant polycystic kidney disease (ADPKD), which occurs with an incidence of 1:500 to 1:1,000 (Solazzo *et al*, 2018) and where the role of cAMP remains enigmatic. ADPKD is caused by mutations in *PKD1* or *PKD2*, encoding for the ciliary proteins polycystin‐1 (PC1) and polycystin‐2 (PC2), respectively (Bergmann, 2017). Levels of cAMP are high in ADPKD patients, and the only approved therapy for the treatment of rapidly progressive ADPKD, tolvaptan, reduces renal cAMP levels and reduces cyst progression in ADPKD (Torres & Harris, 2014; Blair, 2019). Thus, there is a strong association between cAMP signaling and kidney cyst growth, but how cAMP signaling promotes cyst development and its spatial organization in the cell, in particular the contribution of the cilium, is not well defined.

Here, we have applied an integrated approach using optogenetics and next‐generation RNA sequencing (RNA‐seq) and reveal the molecular basis for compartmentalized cAMP signaling and its role in kidney cyst development. By specifically targeting the photoactivated adenylyl cyclase bPAC to primary cilia and contrasting this against bPAC action in the cell body, we identify a novel cAMP signalosome that is functionally distinct from the cytoplasm. In contrast to HH signaling, it promotes a specific gene expression program upon increasing ciliary cAMP levels. We identify the genes and pathways targeted by the ciliary cAMP signalosome, shed light on the underlying mechanisms and downstream signaling, and show that chronic stimulation of the ciliary cAMP signalosome transforms kidney epithelial morphology from tubules into cysts. Counteracting this chronic cAMP elevation in the cilium by small molecules that activate long isoforms of phosphodiesterase 4 (PDE4) inhibits cyst development. Thereby, we identify a novel concept of how the primary cilium controls cellular functions and maintains tissue integrity in a specific and spatially distinct manner. The identification of these new molecular components in cilia‐specific epithelial cell remodeling might provide new insights into the pathomechanism underlying one of the most common genetic diseases, polycystic kidney disease.

## Results

### Spatial manipulation of cAMP levels using optogenetics

**Figure 1:**
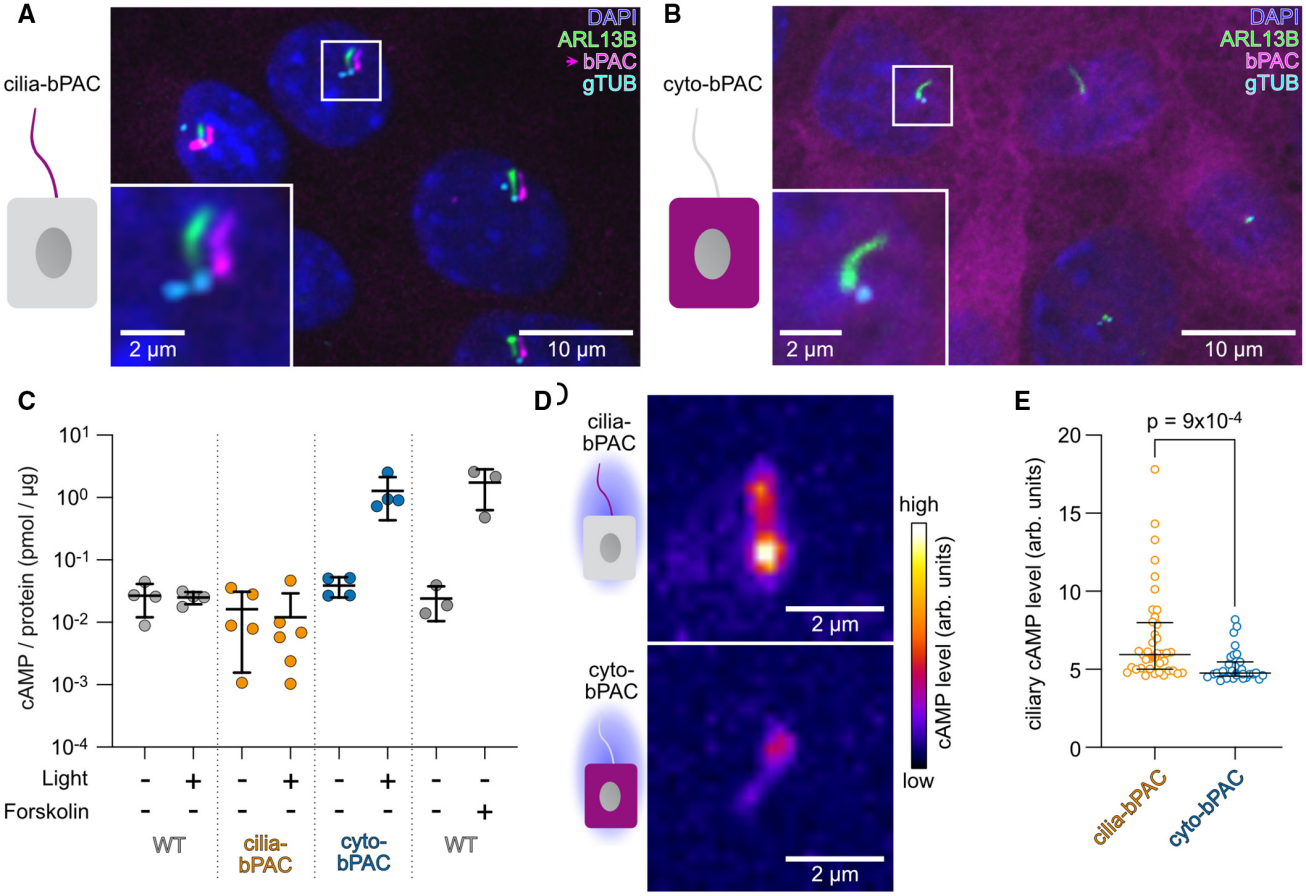
Spatial manipulation of cAMP levels using optogenetics A, BStable mIMCD‐3 cell lines expressing (A) mNphp3(1‐201)‐bPAC‐mCherry (magenta, cilia‐bPAC), which localizes to the primary cilium, or (B) bPAC‐mCherry (magenta, cyto‐bPAC), which resides in the cytoplasm. Cells were labeled with DAPI (blue) to label the DNA, an ARL13B antibody (green) to label cilia, and a gamma‐Tubulin antibody (gTub, cyan) to label the microtubule‐organizing centers. The box indicates the position of the magnified view shown at the bottom left. Magenta arrow indicates the direction and the length of the shift of the respective fluorescence channel. Scale bars are indicated.CELISA‐based measurements of total cAMP levels from wild‐type (WT), cilia‐bPAC, or cyto‐bPAC mIMCD‐3 cells kept in the dark or light‐stimulated (1 h, 465 nm, 38.8 µW/cm²) and WT cells stimulated with DMSO (as control) or 10 µM of Forskolin (1 h). Each data point represents an individual biological replicate (*n* = 4–6 biological replicates). Bars display mean ± SD.D, EMeasurements of ciliary cAMP levels using cilia‐Pink Flamindo. (D) Exemplary images are shown for cilia (cilia‐bPAC or cyto‐bPAC cells) expressing the cilia‐Pink Flamindo biosensor after light stimulation. Scale bars are indicated. cAMP levels are color‐coded using a high‐low look‐up table. (E) Quantification of data exemplified in (D). The cAMP level is determined as the mean ciliary fluorescence intensity during the first 60 s after light stimulation (measurement interval 10 s); *P*‐value for a Kolmogorov‐Smirnov test is indicated. Each data point represents an individual cilium. Bars display median and interquartile range; cilia‐bPAC: 40 cilia (biological replicates) from *n* = 14 independent experiments; cyto‐bPAC: 31 cilia (biological replicates) from *n* = 31 independent experiments.

To investigate the role of ciliary cAMP signaling in epithelial remodeling, we used mIMCD‐3 cells as a model system. We generated mIMCD‐3 cell lines stably expressing the photoactivated adenylyl cyclase mNphp3(201)‐bPAC‐mCherry in the cilium (cilia‐bPAC) (Fig 1A). We have previously shown that bPAC fused to mNphp3(201) can be used to moderately increase ciliary cAMP levels when stimulated by light (Hansen *et al*, 2020). By comparing cilia‐bPAC cells to cells stably expressing bPAC‐mCherry, which localizes to the cytoplasm (cyto‐bPAC, Fig 1B), we aimed to differentially manipulate ciliary versus cytoplasmic cAMP levels. We first verified the light‐stimulated cAMP synthesis in our cell lines: Photoactivation of cyto‐bPAC significantly increased total cAMP levels, similar to stimulation with the transmembrane AC activator Forskolin (Fig 1C). In contrast, photoactivation of cilia‐bPAC did not significantly change total cAMP levels (Fig 1C), which is in line with our earlier findings using a nanobody‐based approach (Hansen *et al*, 2020). We verified the light‐induced increase in ciliary cAMP levels using the genetically encoded biosensor Pink Flamindo (Harada *et al*, 2017). In line with previous reports (Truong *et al*, 2021), photoactivation of cilia‐bPAC increased ciliary cAMP levels to a greater extent than photoactivation of cyto‐bPAC cells (Fig 1D and E). Thus, cilia‐ and cyto‐bPAC are perfectly suited to reveal the molecular basis for compartmentalized cAMP signaling and its role in kidney cyst development.

### Ciliary cAMP signaling drives cyst growth

**Figure 2:**
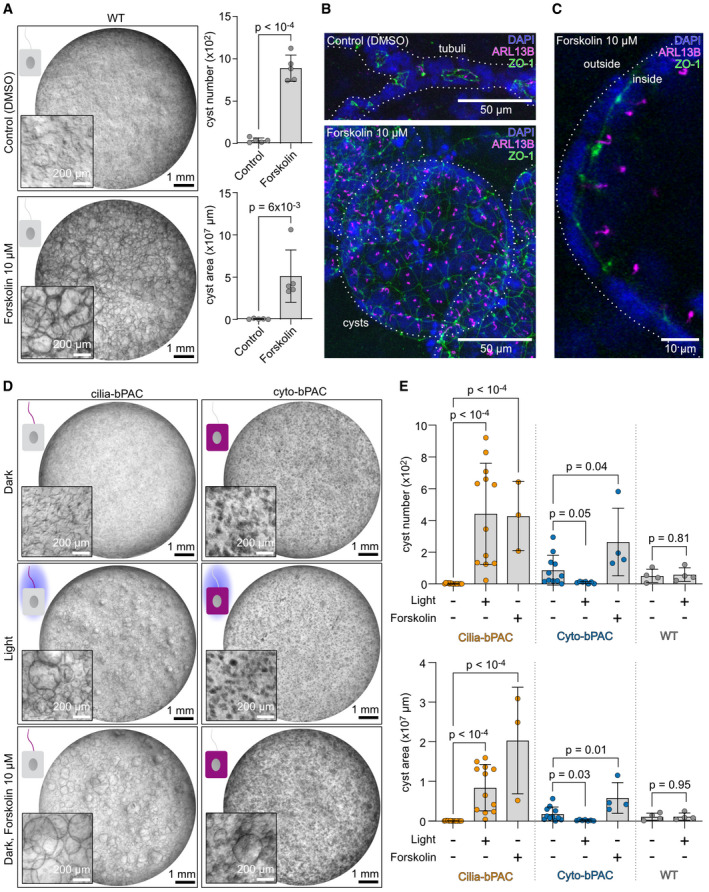
Ciliary cAMP signaling drives cyst growth AWild‐type (WT) mIMCD‐3 cells cultured in a 3D matrix during continuous exposure to DMSO (control) or 10 µM of Forskolin. Exemplary images are shown (*n* = 5 biological replicates). Quantification of cyst number and area is shown on the right (quantification approach is illustrated in Appendix Fig S1). Data are shown as mean ± SD, each datapoint corresponds to an independent experiment, *P*‐values calculated using an unpaired, two‐sided Student’s *t*‐test are indicated.B, CImmunocytochemistry of mIMCD‐3 cells cultured in a 3D matrix during continuous exposure to DMSO (control) or 10 µM of Forskolin. Cells were labeled with DAPI (blue) to label the DNA, with an ARL13B antibody (magenta, ciliary marker) to label cilia, and a ZO‐1 antibody (green) to label tight junctions of the epithelium (green). Scale bars are indicated. (B) Maximum intensity projection of a z‐stack through the entire tubulus (top) or cyst (bottom). (C) Projection of confocal slices acquired from a cyst, illustrating that cilia are facing the lumen.DmIMCD‐3 cells stably expressing cilia‐bPAC or cyto‐bPAC, cultured in a 3D matrix in the dark, during light exposure (1 h light/1 h dark, 9 days, 465 nm, 38.8 µW/cm²) or in the dark and incubated with 10 µM of Forskolin. Exemplary images are shown (*n* ≥ 3).EQuantification of the data exemplified in (D). Data are shown as mean ± SD, each datapoint corresponds to an independent experiment; *P*‐values calculated using an unpaired, two‐sided Student’s *t*‐test are indicated.

To investigate the role of ciliary cAMP levels in epithelial cyst development, we applied a matrix‐based 3D culture system, which has been widely used as an *in vitro* model (Elberg *et al*, 2012). We confirmed that a chronic increase of cAMP levels using Forskolin, which non‐selectively activates ACs, promoted cyst growth (Fig 2A) (Elberg *et al*, 2012). Using immunofluorescence labeling, we verified that the 3D approach maintains the integrity of epithelial cell polarity and adherens junctions. It also promotes primary cilia formation on the luminal apical membrane (Fig 2B and C), recapitulating the *in vivo* situation. Without stimulation, cells formed tubular structures, whereas in the presence of Forskolin, large cysts formed with primary cilia facing the lumen (Fig 2B and C). Thus, chronic stimulation of AC activity and cAMP signaling emulate the disease drive in ADPKD, providing a relevant cellular *in vitro* model for cAMP‐dependent cyst growth with the limitation that it only includes one cell type, that is, mIMCD‐3 cells.

**Figure EV1:**
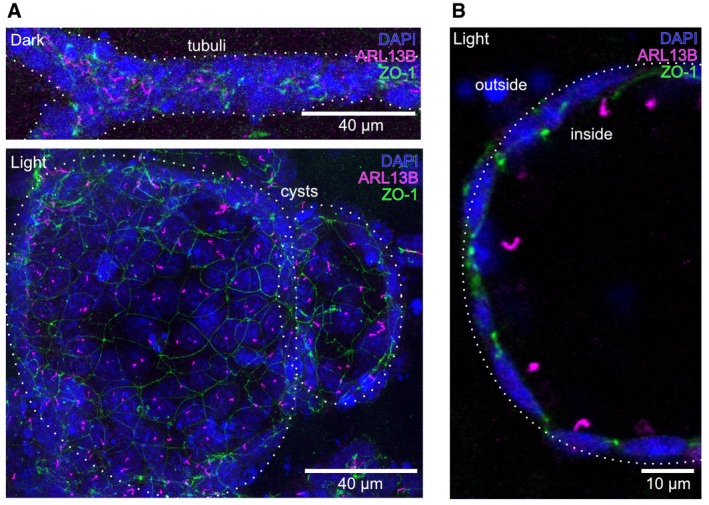
Ciliary cAMP signaling drives cyst growth A, BImmunocytochemistry of mIMCD‐3 cells stably expressing cilia‐bPAC, cultured in a 3D matrix in the dark or during light exposure (1 h light/1 h dark, 9 days, 465 nm, 38.8 µW/cm²). Cells were labeled with DAPI (blue) to label the DNA, with an ARL13B antibody (magenta, ciliary marker) to label cilia, and a ZO‐1 antibody (green) to label tight junctions of the epithelium. Scale bars are indicated. (A) Maximum intensity projection of a z‐stack through the entire tubule (top) or cyst (bottom). (B) Projection of confocal slices acquired from a cyst. Scale bars are indicated.

When applying cilia‐bPAC or cyto‐bPAC in mIMCD‐3 cells cultured in 3D, we were astonished by the results: in contrast to the long‐standing supposition that whole‐cell cAMP elevation drives cystogenesis (Wallace, 2011), we observed that photoactivation of cilia‐bPAC, but not cyto‐bPAC, triggered cystogenesis, as determined by an increase in cyst number and the area covered by cysts (Fig 2D and E). Our results uncover that compartmentalized cAMP signaling underlies cyst development. Importantly, we verified that both cell types are in principle responsive to a pharmacological cAMP stimulus in the dark: Forskolin stimulated cyst development in both cell lines (Fig 2D and E). Furthermore, in cysts induced by photoactivation of cilia‐bPAC, primary cilia were also facing the lumen, similar to the organization observed after Forskolin stimulation (Fig 1, EV1). Thus, our results demonstrate for the first time that a specific increase in ciliary cAMP levels is sufficient to induce cyst growth in a relevant cellular model for renal cyst development.

### Ciliary cAMP signaling controls cell proliferation via mTOR signaling

**Figure 3:**
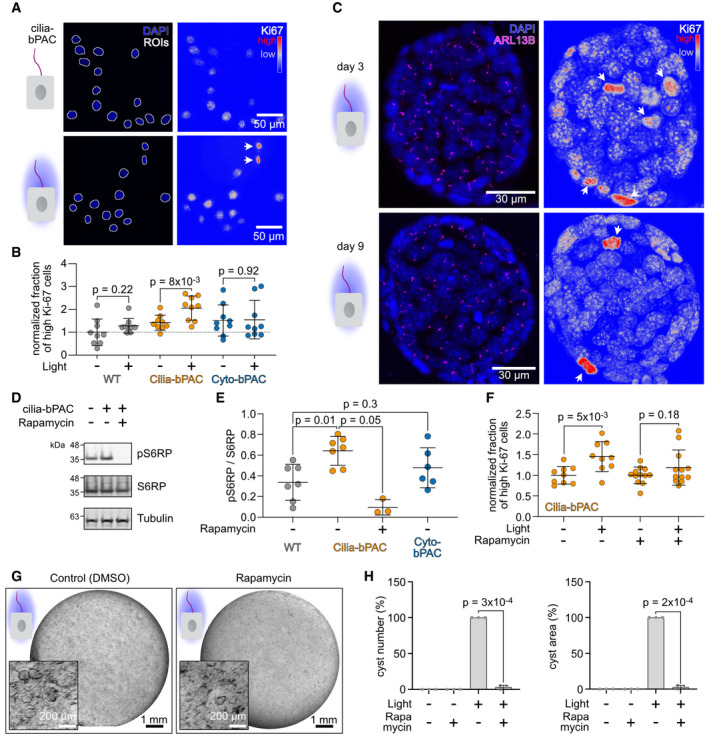
Ciliary cAMP signaling controls cell proliferation via mTOR signaling A–CAnalysis of Ki‐67 expression in wild‐type (WT) and cilia‐bPAC mIMCD‐3 cells, kept in the dark or stimulated with light (1 h light/1 h dark, 72 h, 465 nm, 38.8 µW/cm²). (A) Cells were labeled with DAPI (blue) to label the DNA and a Ki‐67 antibody (look‐up table indicated). The DAPI channel was used as a mask (determined single‐cell ROIs indicated) for the Ki‐67 signal. Arrows indicate cells considered as Ki‐67 high cells in the quantification shown in (B). Scale bars are indicated. (B) Quantification of Ki‐67 high cells. The fraction of Ki‐67 high cells was calculated in wild‐type (WT), cilia‐bPAC, and cyto‐bPAC mIMCD‐3 cells, kept in the dark or after light stimulation and the fold change compared to WT dark was calculated. Data are shown as mean ± SD, *n* = 9, 134–5,509 (median: 702) cells were analyzed per condition and experiment, each datapoint corresponds to an individual experiment; *P*‐values were calculated using an unpaired, two‐sided Student’s *t*‐test. (C) Immunocytochemistry of mIMCD‐3 cells stably expressing cilia‐bPAC, cultured in a 3D matrix during continuous exposure to light for 3 days (top) or 9 days (bottom) (1 h light/1 h dark, 48 h, 465 nm, 38.8 µW/cm²). Cells were labeled with DAPI (blue) to label the DNA, with an ARL13B antibody (magenta) to label cilia, and a Ki‐67 antibody (shown on right, look‐up‐table indicated). Arrows indicate Ki‐67 high cells.DImmunoblotting of lysates from wild‐type (WT) and cilia‐bPAC mIMCD‐3 cells for S6 ribosomal protein (S6RP) phosphorylation at Ser235/236 (pS6RP). Levels of total S6RP protein (S6RP) and beta‐Tubulin have been determined as controls. Cilia‐bPAC cells were treated with DMSO (control) or with 10 nM of Rapamycin. All cells were stimulated with light (1 h light/1 h dark, 48 h, 465 nm, 38.8 µW/cm²).EQuantification of the pS6RP/S6RP ratio. Data are shown as mean ± SD, *n* > 3; *P*‐values were calculated using a paired, two‐sided Student’s *t*‐test. Each data point shows an independent experiment.FQuantification of Ki‐67 high cells. The fraction of Ki‐67 high cells was calculated in cilia‐bPAC mIMCD‐3 cells kept in the dark or stimulated with light and in the presence or absence of 10 nM rapamycin. The fold change compared to cilia‐bPAC dark was calculated. Data are shown as mean ± SD, *n* = 9, 134–16,781 (median: 1,309) cells were analyzed per condition and experiment, each data point corresponds to an individual experiment; *P*‐values were calculated using an unpaired, two‐sided Student’s *t*‐test. Data for cilia‐bPAC in the dark and after light stimulation (both without rapamycin) has been taken from (B) but normalized as indicated here.GmIMCD‐3 cells stably expressing cilia‐bPAC, cultured in a 3D matrix in the dark or during light exposure (1 h light/1 h dark, 9 days, 465 nm, 38.8 µW/cm²) and incubated with or without 10 nM rapamycin. Light exposure started 2 days later than incubation with 10 nM rapamycin. Exemplary images are shown (*n* = 3).HQuantification of the data exemplified in (G). Data are shown as mean ± SD, each datapoint corresponds to an independent experiment; *P*‐values calculated using a paired, two‐sided Student’s *t*‐test are indicated.

Epithelial cell proliferation is key for cyst development in ADPKD (Reif & Wallace, 2019). Thus, we investigated whether an increase in ciliary cAMP levels promotes cell proliferation and performed a fluorescent assay using the proliferation marker Ki‐67. Photoactivation of cilia‐bPAC cells, but not cyto‐bPAC cells, significantly increased Ki‐67 labeling compared to control cells (Fig 3A and B). In 3D culture, cysts formed by photoactivation of cilia‐bPAC also showed Ki‐67 high cells throughout cyst development (Fig 3C). Thus, ciliary cAMP signaling controls cell proliferation.

Cell proliferation in ADPKD has been proposed to be driven by mTOR signaling (Ye *et al*, 2017). We hypothesized that ciliary cAMP signaling controls cell proliferation via stimulating mTOR signaling. To test this experimentally, we determined phosphorylation levels of the S6 ribosomal protein (S6RP) as a read‐out for mitogenic mTOR signaling. Photoactivation of cilia‐bPAC cells, but not cyto‐bPAC cells, increased levels of phosphorylated S6RP compared to control cells (Fig 3D and E). The increase was fully blocked by the mTOR inhibitor rapamycin (Fig 3D and E). Furthermore, in the presence of rapamycin, photoactivation of cilia‐bPAC did not induce cell proliferation (Fig 3F). In turn, rapamycin also inhibited ciliary cAMP‐dependent cyst development (Fig 3G and H). In summary, our results reveal that ciliary cAMP signaling engages mTOR signaling to promote cell proliferation during cyst development.

### Ciliary cAMP signaling is maintained by long‐isoform PDE4 activity

**Figure 4:**
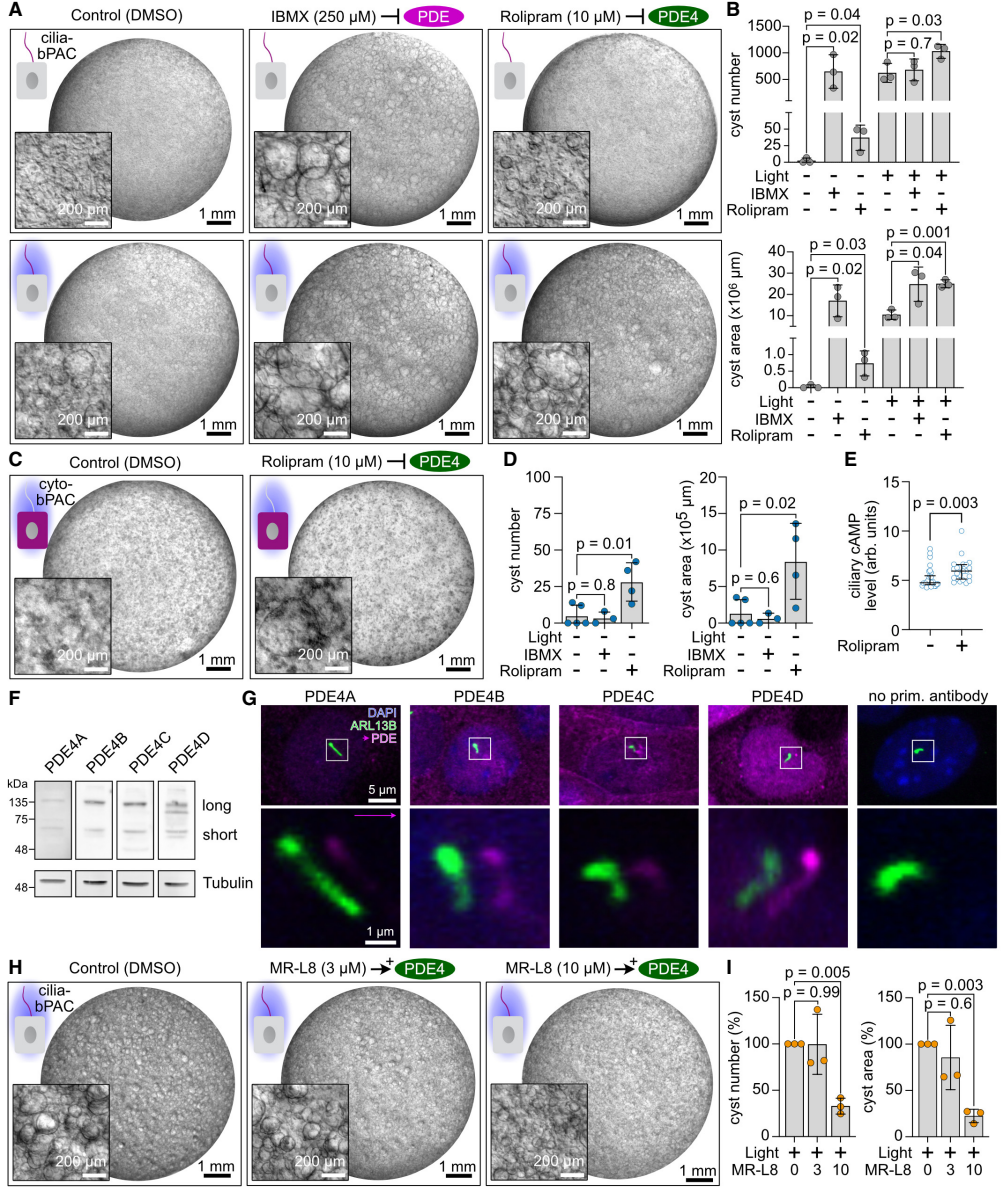
Ciliary cAMP signaling is maintained by isoform‐specific PDE4 activity mIMCD‐3 cells stably expressing cilia‐bPAC, cultured in a 3D matrix in the dark or during light exposure (1 h light/1 h dark, 9 days, 465 nm, 38.8 µW/cm²) and incubated with DMSO (control), 250 µM IBMX, a ubiquitous PDE inhibitor, or 10 µM rolipram, a PDE4‐specific inhibitor. Exemplary images are shown (*n* = 3). Scale bars are indicated.Quantification of data exemplified in (A). Data are shown as mean ± SD, *P*‐values calculated using an unpaired, two‐sided Student’s *t*‐test are indicated. Data points show individual experiments.mIMCD‐3 cells stably expressing cyto‐bPAC, cultured in a 3D matrix during light exposure (1 h light/1 h dark, 9 days, 465 nm, 38.8 µW/cm², 1 day after pharmacological stimulus) and incubation with DMSO or 10 µM rolipram. Exemplary images are shown (*n* = 4–5). Scale bars are indicated. A matching 3D culture, incubated with 250 µM IBMX, is shown in Fig EV2B.Quantification of data exemplified in (C) and Fig EV2B (250 µM IBMX). Data are shown as mean ± SD, *P*‐values calculated using an unpaired Student’s *t*‐test are indicated. Data points show individual experiments.Measurements of ciliary cAMP levels using cilia‐Pink Flamindo. mIMCD‐3 cells expressing cyto‐bPAC have been pre‐treated with 10 μM rolipram or DMSO (ctrl) for 10 min before the measurement. Ciliary cAMP levels have been determined as the mean ciliary fluorescence intensity during the first 60 s after light stimulation (measurement interval 10 s); *P*‐value for a Kolmogorov‐Smirnov test is indicated. Each data point represents an individual cilium (31 cilia (biological replicates) from *n* = 12 independent experiments (DMSO) and 32 cilia (biological replicates) from *n* = 6 independent experiments (rolipram)). Bars display median and interquartile range.Analysis of PDE4 isoform expression (long and short isoforms) in wild‐type mIMCD‐3 cells, revealed by immunoblotting against PDE4A, PDE4B, PDE4C, and PDE4D. The original immunoblots are shown in Appendix Fig S2.Immunocytochemical labeling of mIMCD‐3 cells with antibodies against PDE4A‐D (pink) and an ARL13B antibody (green) to label cilia. DAPI (blue) was used to label the DNA. Pink arrow indicates the direction and the length of the shift of the channel showing the PDE4 labeling. The box indicates the position of the magnified view shown on the bottom of each panel. The magnified views show only one confocal plane, whereas the whole image is shown as maximum‐intensity projection. The control shows cells only stained with the secondary antibody.mIMCD‐3 cells stably expressing cilia‐bPAC, cultured in a 3D matrix during light exposure (1 h light/1 h dark, 9 days, 465 nm, 38.8 µW/cm², started 1 day after pharmacological stimulus) and incubation with DMSO (0 µM), 3 µM, or 10 µM of the PDE4‐long‐isoform activator MR‐L8. Exemplary images are shown (*n* = 3). Scale bars are indicated. The respective dark controls are shown in Fig EV2E and F.Quantification of data exemplified in (H). Data are shown as mean ± SD, *P*‐values calculated using a paired, two‐sided Student’s *t*‐test are indicated. Data have been normalized to the light, 0 µM MR‐L8 condition (Set to 100%) within each experiment. Data points show individual experiments.

The activity of cAMP phosphodiesterases (PDEs) represents the only known means to degrade intracellular cAMP levels, and their expression and localization is crucial to maintain cAMP homeostasis and compartmentalized cAMP signaling (Houslay, 2010). Thus, we sought to investigate whether the ciliary cAMP‐signaling compartment is maintained by the activity of cAMP PDEs. We first assessed whether PDEs in general contribute to cystogenic cAMP signaling in mIMCD‐3 cells. The non‐selective PDE inhibitor 3‐isobutyl‐1‐methylxanthine (IBMX) induced cyst growth in cilia‐bPAC cells in the dark (Fig 4A and B), demonstrating that PDE activity maintains basal ciliary cAMP levels. Furthermore, cyst development in cilia‐bPAC cells was exacerbated by IBMX during light stimulation (Fig 4A and B).

**Figure EV2:**
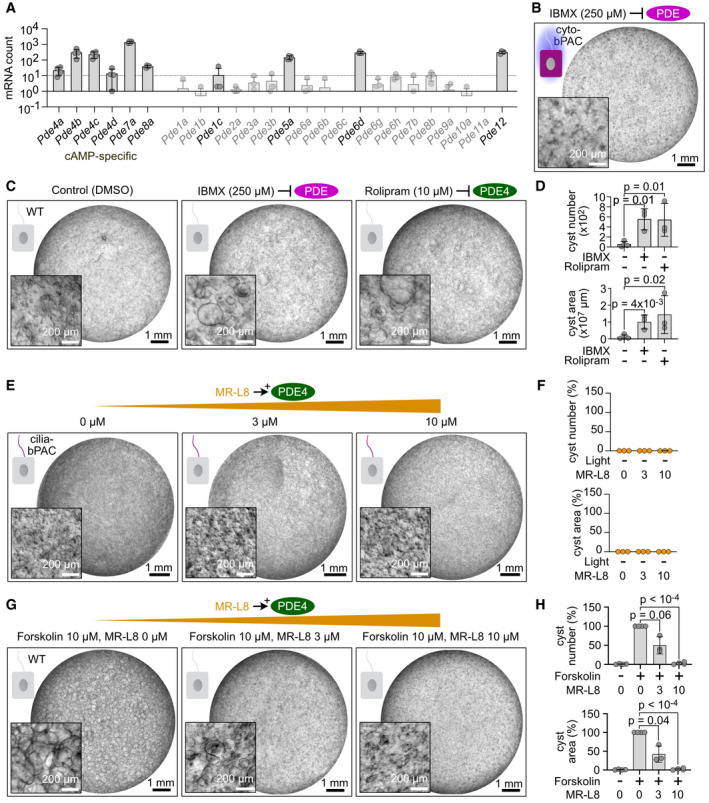
The role of PDE4 isoforms in ciliary cAMP‐dependent cyst formation Analysis of PDE expression in wild‐type mIMCD‐3 cells, determined by unbiased RNA‐Sequencing. PDEs specific for cAMP are indicated. Data are shown as mean ± SD. Data points show individual experiments.mIMCD‐3 cells stably expressing cyto‐bPAC, cultured in a 3D matrix during light exposure (1 h light/1 h dark, 9 days, 465 nm, 38.8 µW/cm², started 1 day after pharmacological stimulus) and incubated with 250 µM IBMX. Exemplary image shown (*n* = 3 experiments). Scale bars are indicated. Matching 3D cultures, incubated with DMSO or 10 µM rolipram, are shown in Fig 4C.Wild‐type (WT) mIMCD‐3 cells, cultured in a 3D matrix and incubated with DMSO (control), 250 µM IBMX, or 10 µM rolipram. Exemplary images are shown (*n* = 3‐4 experiments). Scale bars are indicated.Quantification of images exemplified in (C). Data are shown as mean ± SD, data points show individual experiments. *P*‐values were calculated using a ratio‐paired, two‐sided Student’s *t*‐test.3D culture of mIMCD‐3 cells stably expressing cilia‐bPAC in the dark and incubated with increasing concentrations of the PDE4 long‐isoform activator MR‐L8. Exemplary images are shown (*n* = 3 independent experiments). Scale bars are indicated.Quantification of images exemplified in (E). Data are shown as mean ± SD, data points show individual experiments.3D culture of wild‐type mIMCD‐3 cells incubated with 10 μM Forskolin and increasing concentrations of MR‐L8. Exemplary images are shown (*n* = 3). Scale bars are indicated.Quantification of images exemplified in (G). Data were normalized to 10 μM Forskolin/0 μM MR‐L8 (set to 100%) and are shown as mean ± SD. Data points show individual experiments. *P*‐values were calculated using a paired, two‐sided Student’s *t*‐test.

To identify which PDE isoforms contribute to cystogenic cAMP signaling, we analyzed the expression levels of cAMP‐specific PDEs. At mRNA level, mIMCD‐3 cells express transcripts encoded by *Pde4a, b*, *c*, and *d* as well as *Pde7a* and *Pde8a* (Fig EV2A). It has been previously demonstrated that isoforms of PDE4C localize to primary cilia and associate with the PC1/PC2 complex in mIMCD‐3 cells (Choi *et al*, 2011). PDE4 activity has also been shown to play an important role in cAMP‐mediated cyst growth in Madin‐Darby canine kidney (MDCK) and primary cells derived from human ADPKD patients (Omar *et al*, 2019). We therefore examined if PDE4 activity contributes to the PDE‐dependent control of cystogenic cAMP signaling in primary cilia. Indeed, the PDE4 selective inhibitor rolipram induced cyst growth in cilia‐bPAC cells in the dark and exacerbated light‐stimulated, cilia cAMP‐driven cyst growth (Fig 4A and B). Furthermore, in the presence of rolipram, but not IBMX, photoactivation of cyto‐bPAC induced cyst formation (Figs 4C and D, and EV2B), whereas both rolipram and IBMX evoked cyst formation in wild‐type mIMCD‐3 cells (Fig EV2C and D). This shows that in particular PDE4‐regulated cAMP signaling is required for cystogenesis, whereas cAMP signaling regulated by diverse PDEs rather impairs cyst formation. Strinkingly, rolipram increased ciliary cAMP levels in cyto‐bPAC cells (Fig 4E), indicating that PDE4 activity is crucial to functionally separate the ciliary cAMP compartment from the cytoplasm. If PDE4 activity is inhibited, cytosolic cAMP can accumulate in the cilium and evoke a ciliary cAMP response, that is, cyst formation. This demonstrates that PDE4 activity constitutes a significant proportion of the PDE‐mediated compartmentalization of cAMP in primary cilia and elicits an important role in controlling ciliary cAMP signaling and cyst development.

The PDE4 enzyme family comprises over 20 different isoforms, encoded across four distinct sub‐family genes (*Pde4a‐d*) (Conti *et al*, 2003; Houslay *et al*, 2007). These isoforms can be grouped into an overarching regulatory structure into long, short, and super‐short isoforms (Houslay *et al*, 2007). To profile *Pde4* isoform expression, we performed Western blot analysis using antisera, which specifically recognize enzymes of each of the four different PDE4 sub‐families. Our results demonstrated that mIMCD‐3 cells express the long and short PDE4A‐D isoforms (Fig 4F). Strikingly, all four protein isoforms were enriched in primary cilia of wild‐type mIMCD‐3 cells (Fig 4G).

A previous study showed that a novel therapeutic approach, which focuses on the specific activation of long isoforms of PDE4 by the small molecule MR‐L2 reduced cellular cAMP levels and, thereby, suppressed cAMP‐mediated cyst development in MDCK cells and in ADPKD patient‐derived kidney epithelial cells (Omar *et al*, 2019). Importantly, long‐isoforms of PDE4C are known to localize to primary cilia together with PC1/PC2 complexes, placing these enzymes at a critical location to modulate the underlying disease driver in ADPKD. To investigate whether PDE4 long isoforms control ciliary cAMP signaling and in turn, cyst growth, we incubated cilia‐bPAC cells with MR‐L8, a novel compound based on MR‐L2, during photoactivation of cilia‐bPAC cells. Indeed, MR‐L8 inhibited cyst development evoked by photoactivation of cilia‐bPAC (Figs 4H and I, and EV2E and F) and also by Forskolin (Fig EV2G and H), demonstrating that PDE4 long isoforms predominantly control cyst growth by regulating cAMP signaling in the primary cilium.

### Stimulation of endogenous ciliary GPCR signaling mimics the effect of cilia‐bPAC

**Figure 5:**
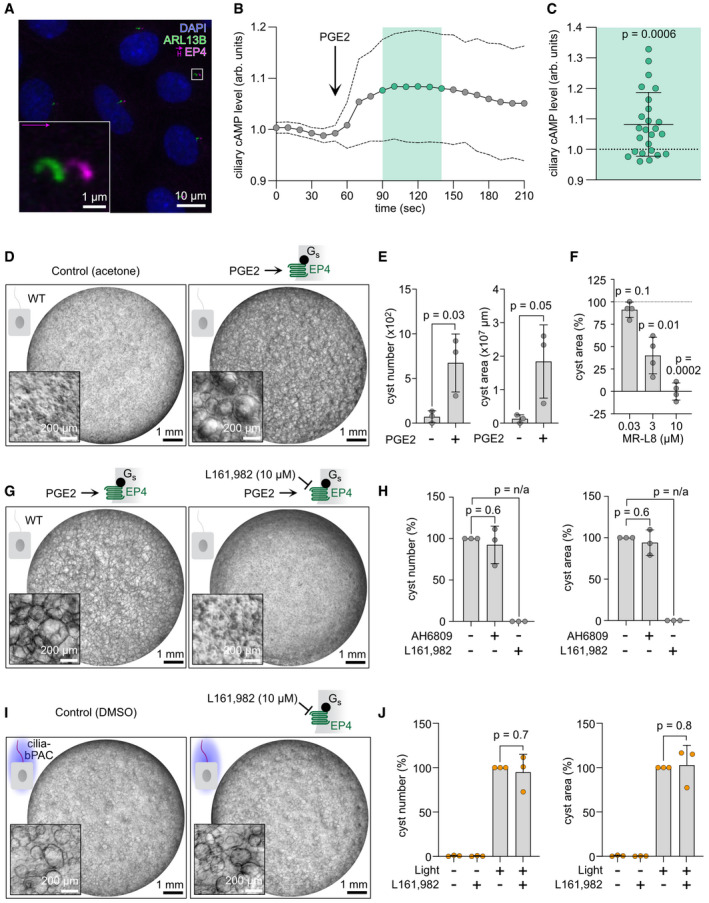
Ciliary GPCR stimulation phenocopies ciliary bPAC‐driven cyst growth Ciliary localization of the EP4 receptor in mIMCD‐3 cells. Immunocytochemical labeling of wild‐type (WT) mIMCD‐3 cells with an anti‐EP4 antibody (pink) and an ARL13B antibody (green) to label cilia. DAPI (blue) was used to label the DNA. The pink arrow indicates the direction and the length of the shift of the channel showing the EP4 labeling. The box indicates the position of the magnified view shown on the bottom left. The scale bars are indicated.Ciliary cAMP dynamics in mIMCD‐3 WT cells measured using 5‐HT6‐mCherry‐cADDis. After baseline measurements, cells were stimulated with 50 nM of Prostaglandin E2 (PGE2). The ratio of ciliary mCherry/cpEGFP fluorescence, normalized to the first 30 s of the recording, is shown as mean (points, solid line) ± SD (dotted lines) of 25 cilia (biological replicates) from *n* = 6 independent experiments.Ciliary cAMP increase after PGE2 stimulation. Each data‐point represents one cilium, data is shown as mean normalized ratio ± SD of the time points indicated in light green in (B); *P*‐value was calculated using a one‐sample *t*‐test compared to unity.3D culture of WT mIMCD‐3 cells continuously exposed to acetone (control) or 100 nM PGE2. Exemplary images are shown (*n* = 3 biological replicates).Quantification of images exemplified in (D). Data are shown as mean ± SD, *P*‐values calculated using an unpaired, two‐sided Student’s *t*‐test are indicated. Data points show individual biological replicates.Quantification of the cyst area in PGE2‐stimulated WT mIMCD‐3 cells in the presence of the PDE4 long isoform‐specific activator MR‐L8. Data were normalized to PGE2‐ (set to 100%) and control‐treated (set to 0%) cells and are shown as mean ± SD, *P*‐values calculated using a one‐sample *t*‐test compared to 100% are indicated, *n* = 4 biological replicates.3D culture of WT mIMCD‐3 cells continuously exposed to acetone (control) or 100 nM PGE2, and DMSO (control) or 10 µM of the EP4 inhibitor L161,982. PGE2 treatment started 2 days after seeding. Exemplary images are shown (*n* = 3). Scale bars are indicated.Quantification for images exemplified in (G), including addition of 3 µM AH6809, an EP2 inhibitor (images see Fig EV3C). Data are shown as mean ± SD, *P*‐values calculated using a paired, two‐sided Student’s *t*‐test are indicated (*P*‐value of n/a indicates that the test is not applicable because both conditions showed no variance). Data points show individual experiments and have been normalized to addition of 100 nM PGE2 (set to 100%) within each experiment.3D culture of mIMCD‐3 cells stably expressing cilia‐bPAC, incubated with DMSO (control) or the EP4‐inhibitor L161,982 in the dark or during light exposure (1 light/1 h dark, 9 days, 465 nm, 38.8 µW/cm², started 2 days after pre‐incubation with DMSO or L161,982). Exemplary images are shown (*n* = 3).Quantification of images exemplified in (I). Data are shown as mean ± SD, *P*‐values calculated using a paired, two‐sided Student’s *t*‐test are indicated. Data points show individual experiments and have been normalized to the light/DMSO condition (set to 100%) within each experiment.

**Figure EV3:**
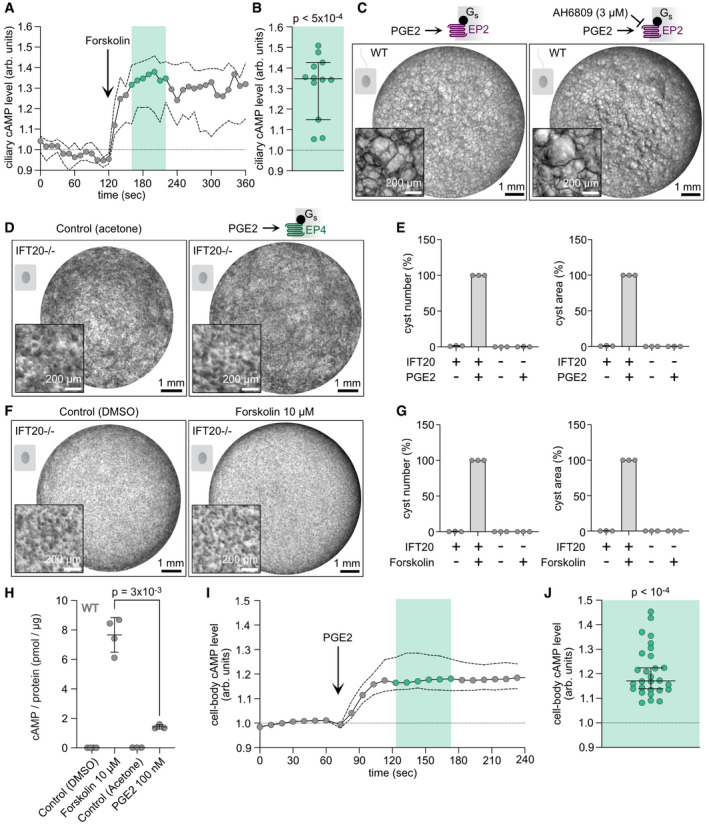
Forskolin increases ciliary cAMP levels Ciliary cAMP dynamics in wild‐type (WT) mIMCD‐3 cells measured using 5‐HT6‐mCherry‐cADDis. After baseline measurements, cells were stimulated with 40 μM of Forskolin. The ratio of ciliary mCherry/cpEGFP fluorescence, normalized to the first 120 s of the recording, is shown as median (points, solid line) ± interquartile range (dotted lines) of 12 cilia (biological replicates) from *n* = 3 independent experiments.Ciliary cAMP increase after Forskolin stimulation. Each data‐point represents one cilium; data is shown as median normalized ratio ± interquartile range of the time points indicated in light green in (A); *P*‐value is indicated for Wilcoxon Singed Rank Test compared to 1.0.3D culture of wild‐type mIMCD‐3 cells incubated with 100 nM PGE2 and DMSO (control) or 3 µM AH6809, an EP2‐inhibitor. The PGE2 stimulus started 2 days later than AH6809 incubation. Exemplary images are shown (*n* = 3). Scale bars are indicated.3D culture of *Ift20^−/−^* mIMCD‐3 cells incubated with 100 nM PGE2 and acetone (control). Exemplary images are shown (*n* = 3). Scale bars are indicated.Quantification of images exemplified in (D). Data were normalized to matching wild‐type controls (set to 100%) and are shown as mean ± SD. Data points show individual experiments.3D culture of *Ift20^−/−^* mIMCD‐3 cells incubated with 10 µM Forskolin and DMSO (control). Exemplary images are shown (*n* = 3). Scale bars are indicated.Quantification of images exemplified in (F). Data were normalized to matching wild‐type controls (set to 100%) and are shown as mean ± SD. Data points show individual experiments.ELISA‐based measurements of total cAMP levels from wild‐type (WT) mIMCD‐3 stimulated with DMSO or acetone (as control) and 10 µM Forskolin (1 h), or 100 nM PGE2 (1 h). Data are shown as mean ± SD, data points show individual experiments (*n* = 3–4), *P*‐values calculated using a paired, two‐sided Student’s *t*‐test are indicated.Cytoplasmic cAMP levels in wild‐type mIMCD‐3 cells were measured before and after stimulation with 100 nM PGE2 using the cytosolic, non‐ratiometric cADDis cAMP biosensor. Data are shown as ratio 1/cADDis fluorescence and normalized to *t* = 0‐60 s. Data are shown as median (points, solid line) ± interquartile range (dotted lines) of 28 cells (biological replicates) from *n* = 4 individual experiments.Individual values for region highlighted in green in (I). Data are shown as median ± interquartile range; *P*‐value is indicated for Wilcoxon Singed Rank Test compared to 1.0. Data represents 28 cells (biological replicates) from *n* = 4 individual experiments.

To investigate whether stimulation of endogenous GPCR‐G_αs_ signaling in the cilium contributes to cyst growth and mimics the effect of cilia‐bPAC, we chose to stimulate mIMCD‐3 cells with prostaglandin E2 (PGE2) as (i) PGE2 is known to stimulate cystogenesis via the G_as_‐coupled EP4 receptor *in vitro* (Breyer & Breyer, 2001; Elberg *et al*, 2012), (ii) PGE2‐dependent signaling is regulated by the activity of long‐isoforms of PDE4 (Omar *et al*, 2019), and (iii) in zebrafish, hRPE1 cells, and mIMCD‐3 cells, and in primary human and murine islet cells, EP4 was detected in primary cilia (Jin *et al*, 2014; Wu *et al*, 2021). We hypothesized that PGE2‐dependent activation of EP4 receptors in primary cilia increases ciliary cAMP levels, which contributes to cystogenesis. To test this hypothesis, we first verified the subcellular localization of the EP4 receptor in mIMCD‐3 cells and could demonstrate that the EP4 receptor indeed is enriched in primary cilia (Fig 5A). Next, we measured changes in ciliary cAMP levels in mIMCD‐3 cells upon stimulation with PGE2 using the ratiometric ciliary cAMP biosensor 5‐HT_6_‐cADDis: mIMCD‐3 cells displayed a significant increase in ciliary cAMP levels upon stimulation with PGE2 (Fig 5B and C), similar to Forskolin (Fig EV3A and B). Furthermore, PGE2 stimulated cyst growth in mIMCD‐3 cells, which was abolished in the presence of the EP4 blocker L161,982, but not in the presence of AH6809, an EP2 blocker (Figs 5D and E, and G and H, and EV3C). The PDE4 long isoform activator MR‐L8 inhibited PGE2‐dependent cyst growth in a dose‐dependent manner (Fig 5F), underlining the role of PDE4 long isoforms in controlling cAMP signaling in the cilium. To reveal whether PGE2‐dependent cyst formation relies on the presence of primary cilia, we stimulated *Ift20* ^−/−^ mIMCD‐3 cells, which do not form primary cilia, with PGE2. We did not observe cyst formation (Fig EV3D and E), demonstrating that PGE2‐dependent cyst formation is primary cilia dependent. This also applied to treatment with Forskolin, which did not induce cyst formation in *Ift20* ^−/−^ cells either (Fig EV3F and G). Furthermore, cyst growth in cilia‐bPAC cells after light stimulation remained unchanged in the presence of L161,982 (Fig 5I and J), indicating that ciliary cAMP‐dependent cystogenesis is downstream of EP4 stimulation. Of note, PGE2 also increased total and cytoplasmic cAMP levels (Fig EV3H–J), although significantly less than, for example, Forskolin, indicating that PGE2 increases cAMP levels in both compartments, the primary cilium and the cytoplasm. However, our data provide compelling evidence (e.g., cilia‐bPAC but not cyto‐bPAC activation induced cyst formation, EP4 localizes predominantly to the cilium, and PGE2‐induced cyst formation is fully abolished under EP4 blockade) that PGE2‐dependent cyst formation predominantly relies on ciliary cAMP signaling. In summary, a physiological increase of ciliary cAMP levels via stimulation of a GPCR that is enriched in primary cilia, phenocopied the results observed by photoactivation of cilia‐bPAC. This highlights that cilia‐bPAC provides a powerful tool to induce and delineate cystogenic signaling.

### Ciliary cAMP signaling evokes a specific gene‐expression signature controlling cyst growth

**Figure EV4:**
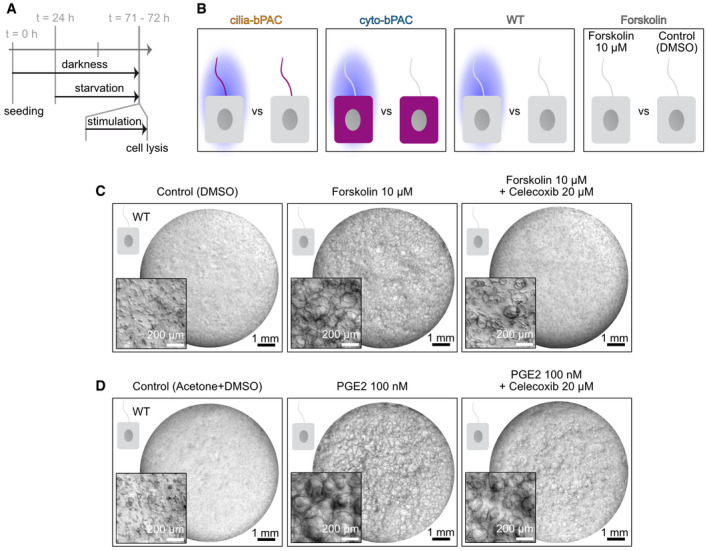
RNA sequencing and signaling downstream of ciliary cAMP Experimental approach. mIMCD‐3 cells were in the dark, starved 24 h post seeding, and stimulated 71 h post seeding for 1 h with the conditions shown in (B), followed by immediate cell lysis.Conditions compared in the transcriptomics experiment: wild‐type (WT) mIMCD‐3 cells and mIMCD‐3 cells stably expressing cilia‐bPAC or cyto‐bPAC were stimulated for 1 h by light (465 nm, 38.8 µW/cm²) or kept in the dark (as control). WT cells were stimulated with DMSO (control) or 10 µM of Forskolin (1 h).Wild‐type (WT) mIMCD‐3 cells cultured in a 3D matrix during continuous exposure to DMSO (control), 10 µM Forskolin, or Forskolin plus 20 μM celecoxib. Exemplary images are shown (*n* = 3).Wild‐type (WT) mIMCD‐3 cells cultured in a 3D matrix during continuous exposure to acetone (control), 10 nM PGE2, or PGE2 plus 20 μM celecoxib. Exemplary images are shown (*n* = 3).

**Figure 6:**
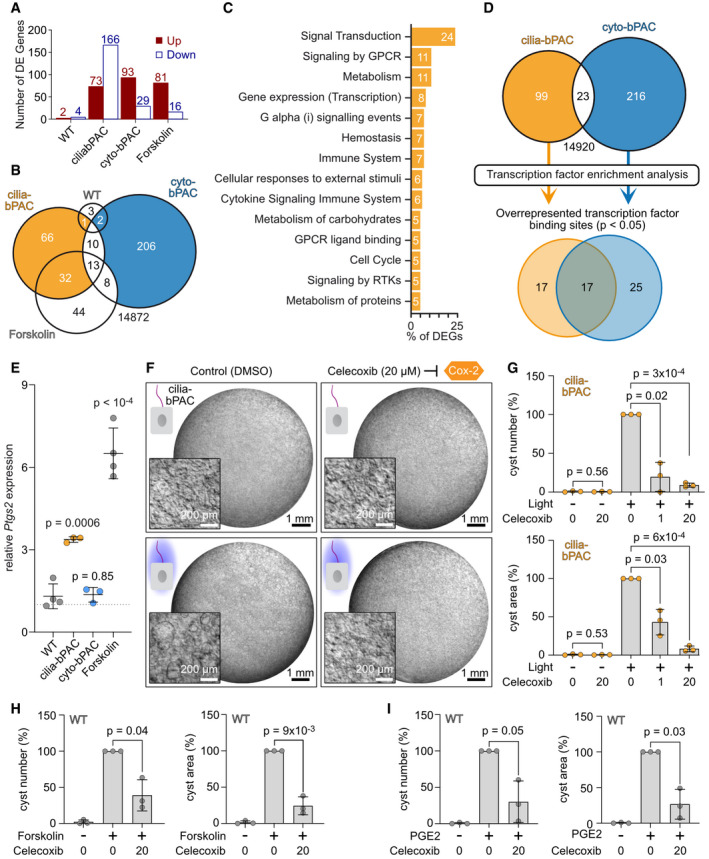
Ciliary cAMP signaling evokes a specific gene expression signature controlling cyst development ADifferentially expressed (DE) genes unraveled by RNA‐Sequence analysis of the conditions shown Fig EV4A and B (*P*‐value < 0.05). Wild‐type (WT), cilia‐bPAC, and cyto‐bPAC: light‐stimulated cells were compared to cells kept in the dark. Forskolin: WT cells stimulated with 10 µM of Forskolin were compared to WT cells incubated with DMSO (control).BVenn diagram of the DE genes; for more details, see Table 1.CParticipation of the DE genes identified in cilia‐bPAC cells in specific reactomes; for more details, see Table 2.DVenn diagram of the transcription factor binding‐sites unraveled by Transcription factor enrichment analysis of the DE genes detected exclusively in cilia‐bPAC or cyto‐bPAC cells; for more details, see Table 3.EQuantitative RT‐PCR analysis. WT, cilia‐bPAC, or cyto‐bPAC cells were treated as shown in Fig EV4A and B. WT, cilia‐bPAC, and cyto‐bPAC: light‐stimulated cells were compared to cells kept in the dark. Forskolin: WT cells stimulated with 10 µM of Forskolin were compared to WT cells incubated with DMSO (control). Data are shown as mean ± SD, *P*‐values were calculated by a two‐sided, unpaired Student’s *t*‐test compared to WT. Data points show individual experiments.F3D culture of mIMCD‐3 cells stably expressing cilia‐bPAC, incubated with DMSO (control) or 20 µM celecoxib, a COX‐2‐inhibitor, in the dark or during light exposure (1 light/1 h dark, 9 days, 465 nm, 38.8 µW/cm², started 2 days after pre‐incubation with DMSO or celecoxib). Exemplary images are shown (*n* = 3). Scale bars are indicated.GQuantification of images exemplified in (F) and from 3D cultures incubated with 1 μM celecoxib. Data are shown as mean ± SD, *P*‐values calculated using a paired, two‐sided Student’s *t*‐test are indicated. Data points show individual experiments.H, IQuantification of Forskolin‐ (H) and PGE2‐dependent (I) cyst formation in the presence or absence of 20 μM celecoxib. Exemplary images are shown in the Fig EV4C and D. Data are shown as mean ± SD, *P*‐values calculated using a paired, two‐sided Student’s *t*‐test are indicated. Data points show individual experiments (*n*=3). Forskolin / PGE2 stimulation started 2 d later than incubation with DMSO (0 µM celecoxib) or 20 µM celecoxib.

To investigate signaling downstream of cAMP and identify the signaling pathways that are specifically engaged by an increase in ciliary cAMP levels, we performed RNA sequencing (RNA‐seq) from cells with increased ciliary (cilia‐bPAC) or cytoplasmic (cyto‐bPAC) cAMP levels and compared them to the respective controls and to Forskolin stimulation (Fig EV4A and B). Our data revealed that increasing cAMP levels in the cilium is sufficient to evoke a change in gene expression: 93 genes were up‐ and 29 genes were down‐regulated (Fig 6A). Increasing cAMP levels in the cell body showed an opposite pattern with 73 genes being up‐and 166 genes being down‐regulated (Fig 6A and Table 1, and Appendix Table S4). Stimulation with Forskolin followed a similar pattern as cilia‐bPAC stimulation with 81 genes being up‐ and 16 genes being down‐regulated (Fig 6A and Table 1, and Appendix Table S4). Light stimulation of wild‐type mIMCD‐3 cells did not significantly change gene expression (Fig 6A and Table 1, and Appendix Table S4). Strikingly, the gene expression program between the different conditions was functionally distinct: Only 23 (19%) of the differentially expressed genes (DEGs) were shared between cilia‐bPAC and cyto‐bPAC and the majority, 99 and 216, respectively, were unique (Fig 6B). Unexpectedly, Forskolin treatment and cilia‐bPAC showed higher commonalities (45 shared DEGs) than Forskolin and cyto‐bPAC (21 shared DEGs; Fig 6B).

**Table 1:** Gene symbols of differentially expressed genes.

Conditions	Gene symbols
cilia‐bPAC only	*8430408G22Rik, Abraxas1, Adgre5, Adora1, Agpat5, Amotl2, Ankrd1, Anks6, Aurka, Bdkrb2, Cables1, Cdc42se2, Chd1, Chp1, Cks2, Coq10b, Csf1, Dclk1, Ddit4l, Dusp14, Dusp4, Dync1h1, Edn1, Efna1, Emx2, Enc1, Eva1b, F2r, Fam171a2, Gm10719, Gm21897, Gpd2, Hivep2, Hs3st1, Jun, Kctd10, Lbh, Lima1, Mafk, Mef2d, Moxd1, N4bp3, Nfxl1, Ngf, Nipal1, Nrbp1, Pax9, Ppif, Ppp2r1b, Pqlc1, Rasl11a, Rgs2, Rnf121, Rpl9‐ps6, Sat1, Sdc3, Sh3bp4, Sik2, Slc3a2, Slit2, Sphk1, Tbx15, Tgif2, Tmem252, Tob2, Trib1*
cilia‐bPAC and Forskolin	*Aff1, Akap12, Atp1b1, Bhlhe40, Cbx4, Cebpd, Crem, Csrnp1, Dlc1, Dusp2, Dusp5, Ets2, Got1, Irf2bp2, Junb, Kcnk1, Klf13, Klf2, Klf4, Nfat5, Nfil3, Nr4a1, Pde4b, Pde4d, Per1, Ptgs2, Ramp3, Sdc4, Spry1, Tcim, Tgif1, Vmn1r217*
cilia‐bPAC and cyto‐bPAC	*Avpi1, Bdnf, Cited2, Coil, Cyr61, Sgo1, Ska2, Slco4a1, Tfap2a, Trim59*
cilia‐bPAC, cyto‐bPAC, and Forskolin	*Dusp1, Dusp10, Errfi1, Fosl2, Gdf15, Hoxb6, Irs2, Nr4a2, Phyhipl, Sgk1, Sik1, Spry2, Thbd*
cyto‐bPAC only	*Aar2, Aco2, Adgra2, Aftph, Ajuba, Akr1c19, Ankrd23, Anxa9, Arid3a, Arid5a, Asxl1, Atf4, Atp5b, Bcar1, Bcl2l11, Bcl3, Bms1, Bora, Brd3os, Btg1, C1d, Casc3, Ccdc14, Ccdc51, Ccnf, Ccnjl, Ccnl1, Cdc42ep1, Cdca4, Cenpl, Chac1, Chaf1b, Chmp6, Cldn12, Cog2, Cstf2t, Ctso, Cwf19l1, D030056L22Rik, D930048N14Rik, Daw1, Dbf4, Ddias, Ddit3, Deaf1, Dgcr14, Dnajb4, Dnajb9, Dnajc16, Dpf2, Dpysl5, Dusp8, E2f8, Edar, Egr1, Eif5, Elmsan1, Epha2, Ercc6, Eri2, Exo5, Fam110a, Fam118a, Fam83d, Fam83h, Fem1c, Fhl1, Fhod3, Fignl1, Filip1l, Fnip2, Gabarapl1, Gjb4, Gpr160, Gprc5c, Gramd4, Haspin, Hbegf, Herpud1, Hist1h2ad, Hist1h2ap, Hist2h3b, Hist2h3c1, Hoxb4, Hoxb5, Hsbp1, Hspbap1, Hyls1, Id1, Ier2, Ifit1bl1, Il17ra, Il17rd, Inpp5f, Irgm1, Irx3, Jrk, Kcnj2, Klf11, Lrif1, Lrp2, Lrrc8c, Luc7l3, Mat2a, Mbip, Mcm6, Med20, Med7, Midn, Mios, Mis12, Mphosph10, Mras, Mrpl10, Mtf2, Mtfr2, Mum1, Myb, Nae1, Nek2, Nfe2l2, Nol12, Nup35, Nup62, Nxt1, Pcdhb22, Pcf11, Pgrmc2, Phax, Pik3r2, Pim1, Pitpnm3, Podxl, Pola2, Polr3f, Ppan, Prkar1a, Prorsd1, Rabif, Racgap1, Rasgef1b, Rbbp6, Rbm34, Rbm7, Rbsn, Repin1, Rhbdf1, Rnf6, Rsrp1, Sac3d1, Sdhc, Sema5a, Sertad1, Sertad2, Sesn2, Sh3bp2, Slc25a27, Slc29a2, Slc52a3, Smad7, Sorcs2, Sowahc, Sphk2, Srsf1, Srsf7, Ston2, Syde1, Tada3, Tardbp, Tjap1, Tmem171, Tmem214, Tmem254a, Tnfaip8l1, Toe1, Traf3ip2, Trim16, Troap, Tsc22d1, Tut1, Ube4a, Ubox5, Urb2, Usp33, Usp42, Wee1, Ywhae, Zbtb40, Zbtb42, Zfp113, Zfp119b, Zfp143, Zfp202, Zfp207, Zfp324, Zfp39, Zfp62, Zfp639, Zfp646, Zfp647, Zfp68, Zfp994, Zfp995, Znfx1, Zwint, Zzz3*
cyto‐bPAC and Forskolin	*Arrdc3, Clcf1, Clk1, Ier5l, Ints5, Klf10, Txnip, Zfp623*
Forskolin only	*Adamts1, Adh7, Ano2, Arl4c, Atf3, Bckdk, Btg2, Cdca7, Cdk18, Cnpy4, Ddx28, Dmxl1, Duoxa1, Dusp6, Fos, Foxa1, Foxc1, Gadd45g, Gfra4, Gm12184, Gm20695, Gm21974, Gna12, Gprc5a, Hist1h2bp, Isca1, Jade1, Krt18, Mapk7, Mast4, Odf3b, Osgin1, Pcdhga1, Pim3, Plac1, Rassf10, Sgms2, Spry4, Srf, Stk40, Tbcel, Thbs1, Tiparp, Zfp689*
WT only	*1700020L24Rik, Gm973, Mybl1*
WT and cyto‐bPAC	*Gm20431, Aldh1l1*
WT and cilia‐bPAC	*Gm10696*

Genes represent data shown in Fig 6, grouped according to the Venn Diagram (Fig 6B). Detailed table including log2‐fold changes and adjusted *P*‐values are shown in Appendix Table S4.

To investigate the underlying signaling pathways, we performed a Reactome pathway analysis for the DEGs of cilia‐bPAC (Fabregat *et al*, 2017). The majority of these DEGs were classified as “Signal Transduction,” followed by “Signaling by GPCR,” “Metabolism,” and “Gene Expression” (Fig 6C and Table 2). These results underline the importance of cAMP for GPCR signaling in the cilium, supporting the model of the primary cilium as a signaling hub and cAMP as the second messenger that locally transduces extracellular stimuli into an intracellular response.

**Table 2:** Results from a reactome analysis of the genes differentially expressed in cilia‐bPAC cells after light stimulation for 1 h.

Pathway identifier	Pathway name	Submitted entities found
R‐MMU‐162582	Signal Transduction	*Jun, Edn1, Cbx4, Sdc4, Sdc3, F2r, Amotl2, Pde4b, Bdkrb2, Spry1, Ngf, Dync1h1, Ramp3, Dusp2, Tgif1, Dusp5, Rgs2, Ppp2r1b, Tgif2, Dusp4, Adora1, Pde4d, Sphk1, Nr4a1*
R‐MMU‐388396	GPCR downstream signaling	*Ramp3, Ppp2r1b, Rgs2, Edn1, Sdc4, Sdc3, Adora1, Pde4d, F2r, Pde4b, Bdkrb2*
R‐MMU‐372790	Signaling by GPCR	*Ramp3, Ppp2r1b, Rgs2, Edn1, Sdc4, Sdc3, Adora1, Pde4d, F2r, Pde4b, Bdkrb2*
R‐MMU‐1430728	Metabolism	*Hs3st1, Agpat5, Got1, Sdc4, Gpd2, Sdc3, Chp1, Sphk1, Slc3a2, Sat1, Ptgs2*
R‐MMU‐74160	Gene expression (Transcription)	*Tgif1, Ppp2r1b, Tgif2, Cbx4, Cited2, Aurka, Nr4a1, Nrbp1*
R‐MMU‐73857	RNA Polymerase II Transcription	*Tgif1, Ppp2r1b, Tgif2, Cbx4, Cited2, Aurka, Nr4a1, Nrbp1*
R‐MMU‐212436	Generic Transcription Pathway	*Tgif1, Ppp2r1b, Tgif2, Cbx4, Cited2, Aurka, Nr4a1, Nrbp1*
R‐MMU‐418594	G alpha (i) signaling events	*Ppp2r1b, Sdc4, Sdc3, Adora1, Pde4d, Pde4b, Bdkrb2*
R‐MMU‐168256	Immune System	*Dusp2, Ppp2r1b, Jun, Dusp5, Dusp4, Csf1, Dync1h1*
R‐MMU‐109582	Hemostasis	*Ppp2r1b, Atp1b1, Sdc4, Sdc3, F2r, Slc3a2, Mafk*
R‐MMU‐8953897	Cellular responses to external stimuli	*Jun, Cbx4, Cited2, Sh3bp4, Dync1h1, Ets2*
R‐MMU‐2262752	Cellular responses to stress	*Jun, Cbx4, Cited2, Sh3bp4, Dync1h1, Ets2*
R‐MMU‐1280215	Cytokine Signaling in Immune system	*Dusp2, Ppp2r1b, Jun, Dusp5, Dusp4, Csf1*
R‐MMU‐9006934	Signaling by Receptor Tyrosine Kinases	*Ppp2r1b, Dusp4, Sphk1, Spry1, Ngf*
R‐MMU‐71387	Metabolism of carbohydrates	*Hs3st1, Got1, Sdc4, Sdc3, Chp1*
R‐MMU‐500792	GPCR ligand binding	*Ramp3, Edn1, Adora1, F2r, Bdkrb2*
R‐MMU‐392499	Metabolism of proteins	*Abraxas1, Cbx4, Sphk1, Csf1, Dync1h1*
R‐MMU‐1640170	Cell Cycle	*Abraxas1, Ppp2r1b, Cables1, Dync1h1, Aurka*
R‐MMU‐9607240	FLT3 Signaling	*Dusp2, Ppp2r1b, Dusp5, Dusp4*
R‐MMU‐69278	Cell Cycle, Mitotic	*Ppp2r1b, Cables1, Dync1h1, Aurka*
R‐MMU‐597592	Post‐translational protein modification	*Abraxas1, Cbx4, Csf1, Dync1h1*
R‐MMU‐5684996	MAPK1/MAPK3 signaling	*Dusp2, Ppp2r1b, Dusp5, Dusp4*
R‐MMU‐5683057	MAPK family signaling cascades	*Dusp2, Ppp2r1b, Dusp5, Dusp4*
R‐MMU‐5675221	Negative regulation of MAPK pathway	*Dusp2, Ppp2r1b, Dusp5, Dusp4*
R‐MMU‐5673001	RAF/MAP kinase cascade	*Dusp2, Ppp2r1b, Dusp5, Dusp4*
R‐MMU‐556833	Metabolism of lipids	*Agpat5, Gpd2, Sphk1, Ptgs2*
R‐MMU‐449147	Signaling by Interleukins	*Ppp2r1b, Jun, Dusp4, Csf1*
R‐MMU‐416476	G alpha (q) signaling events	*Rgs2, Edn1, F2r, Bdkrb2*
R‐MMU‐373076	Class A/1 (Rhodopsin‐like receptors)	*Edn1, Adora1, F2r, Bdkrb2*
R‐MMU‐202733	Cell surface interactions at the vascular wall	*Atp1b1, Sdc4, Sdc3, Slc3a2*
R‐MMU‐168249	Innate Immune System	*Ppp2r1b, Jun, Dusp4, Dync1h1*
R‐MMU‐1630316	Glycosaminoglycan metabolism	*Hs3st1, Sdc4, Sdc3, Chp1*

Only reactomes covering at least four of the differentially expressed genes are shown.

To investigate which transcription factors (TFs) are involved in transducing the information and evoking a change in gene expression mediated by ciliary cAMP signaling, we performed a TF enrichment analysis for the DEGs specific for cilia‐bPAC or cyto‐bPAC (Hansen *et al*, 2012): 17 TFs were enriched in both, whereas 17 or 25 were unique for cilia‐bPAC or cyto‐bPAC, respectively (Fig 6D and Table 3). Among the TFs that were uniquely enriched in light‐stimulated cilia‐bPAC cells was CREB, which is activated upon PKA‐induced protein phosphorylation and dimerization (Montminy, 1997). Strikingly, the top hits among the DEGs, which are up‐regulated in cilia‐bPAC, were the direct CREB targets *Ptgs2*, encoding the prostaglandin‐endoperoxide synthase 2 (also termed COX‐2), and *Crem*, encoding for the transcription factor cAMP‐response element modulator. COX‐2 produces prostanoids, that is prostaglandin H2 from arachidonic acid, which is converted to PGE2 by the prostaglandin synthase 1 (PGES1). An increase in COX‐2 expression has also been observed in PKD models (Macianskiene *et al*, 2003; Warford‐Woolgar *et al*, 2006; Zhang *et al*, 2019). We verified the changes in *Ptgs2* gene expression by qPCR‐analysis: *Ptgs2* expression was increased upon photoactivation of cilia‐bPAC or stimulation with Forskolin, whereas photoactivation of bPAC in the cell body did not change *Ptgs2* expression (Fig 6E). To test whether cyst growth induced by ciliary cAMP signaling is dependent on COX‐2, we incubated cilia‐bPAC cells with the COX‐2 inhibitor celecoxib. Inhibition of COX‐2 abolished ciliary cAMP‐dependent and Forskolin‐ and PGE2‐dependent cyst growth (Figs 6F–I and EV4C and D), demonstrating that COX‐2 is located downstream of ciliary cAMP signaling.

**Table 3:** Enriched transcription factor binding‐sites in differentially expressed genes triggered by cilia‐bPAC vs. cyto‐bPAC stimulation.

Condition	Binding site	*P*‐value (cilia bPAC)	*P*‐value (cyto bPAC)
cilia‐bPAC only	E2F2_1	0.024135423	
	E2F3_1	0.031205588	
	Zfp740_1	0.022484632	
	Sp4_2	0.031205588	
	Zfp740_2	0.006719461	
	Zic1_2	0.031205588	
	CREB1	0.024135423	
	MZF1_1‐4	0.00568896	
	RREB1	0.022484632	
	Fos	0.010279422	
	Zfx	0.022484632	
	Glis2_1	0.024135423	
	Gcm1_1	0.041640318	
	Elf3_2	0.031205588	
	Rara_2	0.031205588	
	NF‐kappaB	0.022784748	
	TLX1::NFIC	0.041640318	
cilia‐bPAC and cyto‐bPAC	Egr1_1	0.02413542	0.02025613
	Mtf1_1	0.02413542	0.04509209
	Sp4_1	0.00671946	0.00620063
	Tcfap2b_1	0.01341626	0.01620158
	Zic1_1	0.02248463	0.01097939
	Zic2_1	0.00454803	0.03358469
	Zic3_1	0.00671946	0.01974174
	TFAP2A	0.03810775	0.02372213
	Arnt	0.02413542	0.0272856
	USF1	0.02413542	0.03829769
	Mycn	0.02413542	0.0272856
	Myc	0.02413542	0.04509209
	SP1	0.00454803	0.04630281
	Max_1	0.04164032	0.01974174
	Tcfap2a_1	0.02413542	0.00945648
	Tcfap2c_1	0.04164032	0.00945648
	Irf4_2	0.03120559	0.02372213
cyto‐bPAC only	Zfp161_1		0.01974174
	Zfp161_2		0.04509209
	Klf7_1		0.04509209
	Zfp128_1		0.00945648
	Ascl2_2		0.01974174
	E2F2_2		0.04509209
	Gmeb1_2		0.01974174
	IRC900814_2		0.02372213
	Klf7_2		0.00945648
	Sp100_2		0.00076369
	Tcfap2c_2		0.01620158
	Lhx6_2		0.04509209
	Nkx1‐1		0.03358469
	Arnt::Ahr		0.04630281
	E2F1		0.00977606
	ELK1		0.01672607
	NFYA		0.03358469
	GABPA		0.01672607
	SPI1		1.0641E‐05
	ETS1		2.5309E‐05
	Zfp423		0.01620158
	Mycn		0.04509209
	ELK4		0.04509209
	SRF		0.04509209
	Tcfcp2l1		0.04509209

Binding sites unraveled and *P*‐values reported using the pcaGoPromoter package (Hansen *et al*, 2012). No *P*‐value indicated for *P* > 0.05.

Thus, our data demonstrate that an increase in ciliary cAMP signaling regulates gene expression, inducing a specific gene expression program that is distinct from the program evoked in the cytoplasm and involves targets of the transcription factor CREB, for example, COX‐2, driving ciliary cAMP‐dependent cyst development.

### An increase in ciliary cAMP levels is transduced by PKA‐dependent CREB phosphorylation in the cilium

Next, we aimed to delineate how primary cilia transduce cAMP signaling via the transcription factor CREB. CREB is activated by cAMP/PKA‐dependent phosphorylation (pCREB) (Montminy, 1997). We hypothesized that CREB shuttles through the cilium and is phosphorylated by PKA in a cAMP‐dependent manner—similar to the principle that has been established for the Gli transcription factors in ciliary Hedgehog signaling (Huangfu *et al*, 2003; Mukhopadhyay *et al*, 2013; Niewiadomski *et al*, 2014; Bangs & Anderson, 2017; Jiang *et al*, 2019; Arveseth *et al*, 2021).

**Figure 7:**
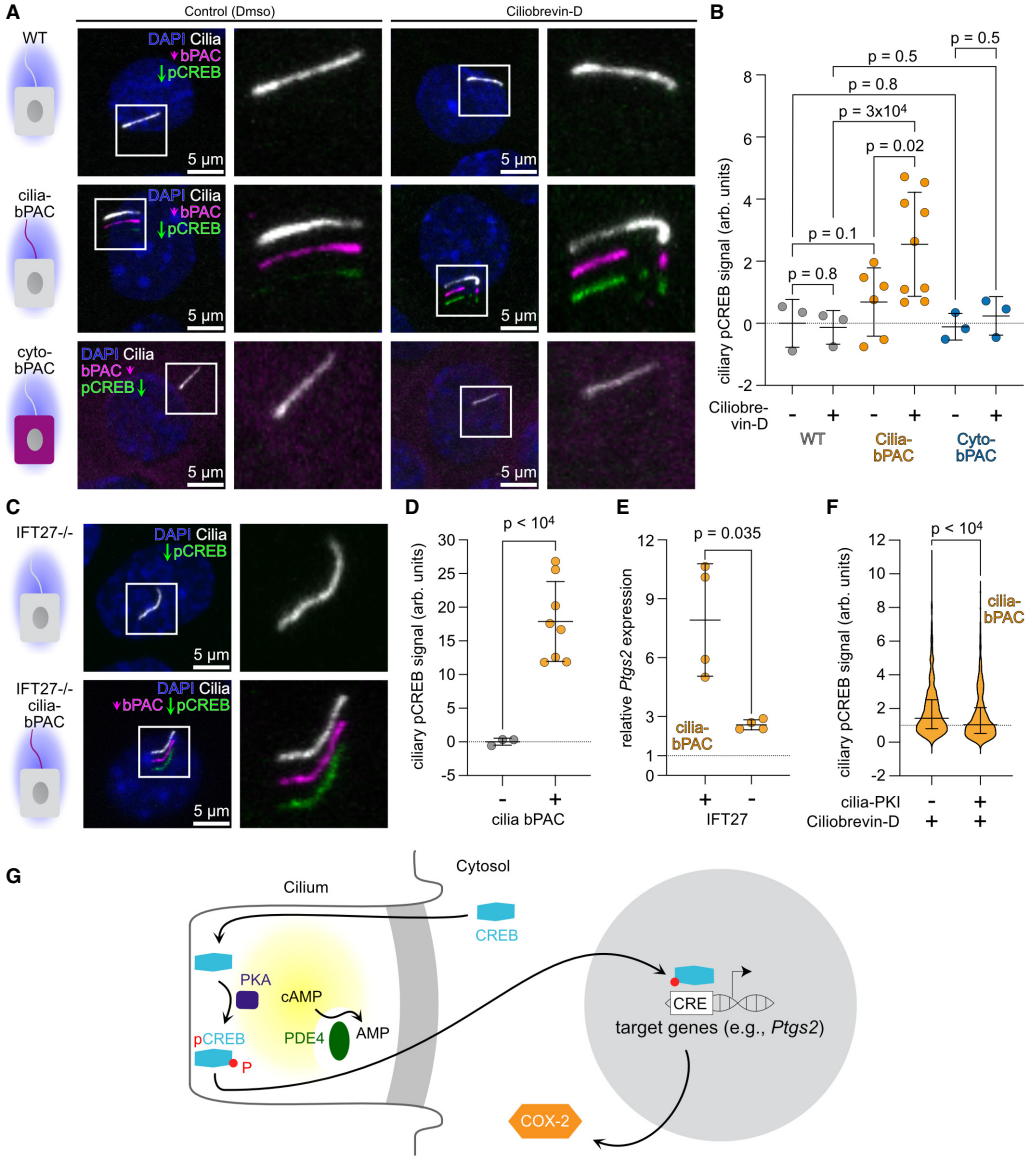
An increase in ciliary cAMP levels is transduced by PKA‐dependent phosphorylation of CREB in the cilium Wild‐type (WT), cilia‐bPAC, or cyto‐bPAC (magenta) mIMCD‐3 cells were stimulated by light (for 1 h prior to fixation, 465 nm, 38.8 µW/cm²), and treated with DMSO (control) or 50 µM Ciliobrevin‐D, a dynein‐inhibitor. Cells were labeled with DAPI (blue) to label the DNA, an ARL13B antibody (white) to label cilia, and a phospho‐specific (Ser133) CREB antibody (pCREB, green). Arrows indicate the direction and the length of the shift of the respective fluorescence channel. The box indicates the position of the magnified view shown on the right of each panel.Quantification of the ciliary pCREB signal in WT, cilia‐bPAC, and cyto‐bPAC cells. Data are shown as mean ± SD, *n* > 3. Each data point represents an independent experiment and corresponds to the median of > 73 cilia (biological replicates); *P*‐values for an unpaired, two‐sided Student’s *t*‐test with Welch’s correction are indicated.
*Ift27^−/−^* mIMCD‐3 cells with or without stable cilia‐bPAC expression (magenta) were stimulated with light (for 1 h prior to fixation, 465 nm, 38.8 µW/cm²). Cells were labeled with DAPI (blue) to label the DNA, an ARL13B antibody (white) to label cilia, and a phospho‐specific (Ser133) CREB antibody (pCREB, green). Arrows indicate the direction and the length of the shift of the respective fluorescence channel. The box indicates the position of the magnified view shown on the right of each panel.Quantification of the ciliary pCREB signal. Data are shown as mean ± SD, *n* > 3. Each data point represents the median of > 90 cilia from the same experiment; *P*‐values are indicated for an unpaired, two‐sided Student’s *t*‐test with Welch’s correction.Quantitative RT‐PCR analysis comparing *Ptgs2* expression in light‐stimulated control (+) or *Ift27^−/−^* (−) mIMCD‐3 cells, both expressing cilia‐bPAC. Data are shown as mean ± SD, *P*‐values were calculated by a two‐sided, unpaired Student’s *t*‐test. Data points show individual experiments.Quantification of the ciliary pCREB signal in mIMCD‐3 cells, expressing the PKA‐inhibiting peptide cilia‐PKI and cilia‐bPAC or cilia‐bPAC only, in the dark and after light stimulation (for 1 h prior to fixation, 465 nm, 38.8 µW/cm²). For each cell line, the ciliary pCREB signal of light‐stimulated cells was normalized to the median pCREB signal of cells in the dark. Data are shown as violin plots, bars indicate median and interquartile range, *n* = 7 (−: 1242 cilia (biological replicates), +: 482 cilia (biological replicates)); *P*‐value for a Kolmogorov Smirnov test is indicated.Scheme indicating the model for ciliary cAMP signaling controlling CREB‐dependent gene expression.

We analyzed CREB phosphorylation and localization using immunocytochemistry in cilia‐bPAC cells: pCREB levels were increased in primary cilia after light stimulation compared to wild‐type control cells (Fig 7A and B). As the increase of pCREB was rather small, we hypothesized that active transport of CREB out of the cilium masked the true extent of CREB activation. To prolong the residence time of proteins in the cilium, we inhibited retrograde ciliary transport by incubating the cells with the dynamin‐2 inhibitor Ciliobrevin‐D. This led to a significant increase of pCREB levels in primary cilia of cilia‐bPAC but not cyto‐bPAC cells after light stimulation (Fig 7A and B). We verified this result using a genetic approach to inhibit retrograde IFT by performing similar experiments in *Ift27^−/−^* mIMCD‐3 cells, which accumulate specific signaling components in the cilium (Eguether *et al*, 2014; Liew *et al*, 2014; Mick *et al*, 2015). Consistently, pCREB localization in the cilium was increased in IFT27‐deficient primary cilia expressing cilia‐bPAC after light stimulation (Fig 7C and D). To demonstrate whether pCREB retention in the cilium inhibited downstream signaling, we analyzed *Ptgs2* expression after photoactivation of cilia‐bPAC in IFT27‐deficient mIMCD‐3 cells and revealed that ciliary cAMP‐dependent *Ptgs2* expression was strongly reduced when receptor retrieval from cilia was impaired (Fig 7E). To investigate whether cAMP‐dependent phosphorylation of CREB occurs in the cilium, we specifically inhibited ciliary PKA activity using a PKA inhibiting peptide localized to cilia (cilia‐PKI) (Mick *et al*, 2015). In the presence of cilia‐PKI, the light‐stimulated increase of ciliary pCREB levels was significantly reduced (Fig 7F).

Thus, our results demonstrate that an increase in ciliary cAMP levels is transduced into a distinct gene expression program via PKA‐dependent phosphorylation of CREB in the primary cilium, which controls gene expression (Fig 7G).

## Discussion

Ciliopathies such as ADPKD are driven by defects in primary cilia signaling (McConnachie *et al*, 2021). Mutations in the PC1/PC2 protein complex result in reduced Ca^2+^ and elevated cAMP levels, which has been associated with cyst development and disease progression (Calvet, 2015). However, whether changes in ciliary cAMP signaling are sufficient to drive cystogenesis, and whether the spatial organization of cAMP signaling in the cilium vs. cytoplasm is important for disease development, remained, until now, unanswered. We have elucidated the contribution of ciliary cAMP signaling to renal cystogenesis and demonstrate, for the first time, that chronic stimulation of a novel ciliary cAMP signalosome transforms epithelial 3D morphology and drives cyst growth *in vitro*. We unravel the molecular mechanisms how a change in cAMP levels is locally transduced in the primary cilium and identify the downstream targets and cellular functions.

Prior to this study, the only ciliary signaling pathway that has been unambiguously accepted to control gene expression by local activation of a transcription factor in the cilium was the Hedgehog (Hh) signaling pathway (Gigante & Caspary, 2020). In the “off” state, the glioma‐associated (GLI) transcription factors GLI2/3 are processed into their repressor forms in a cAMP/PKA‐dependent manner, turning target gene expression off. Upon Hh binding to its receptor Patched 1 (PTCH1), the GLI transcription factors reside in their activate state, turning gene expression on (Arveseth *et al*, 2021). The sequence of the molecular events has been verified using a time‐resolved proteomics approach (preprint: May *et al*, 2021), and the differential interpretation of cAMP signaling in the cilium compared to the cell body for Hh signaling has been recently demonstrated using a combination of chemogenetics and optogenetics (Truong *et al*, 2021). Almost two decades after the discovery of Hh signaling in the cilium (Huangfu *et al*, 2003), we add a new chapter to cilia biology by demonstrating that the transcription factor CREB shuttles through the cilium, where it is phosphorylated by PKA (Fig 7). In contrast to the GLI transcription factors, phosphorylation leads to transcription factor activation, promoting gene expression. This shows that the cilium can differentially interpret high cAMP levels by either repressing or activating gene expression. Our findings introduce the transcription factor CREB as a new component in compartmentalized, ciliary cAMP signaling and underline the role of the primary cilium as a signaling hub that controls cellular functions by changing gene expression.

A recent optogenetic/chemogenetic study proposed that primary cilia geometry, that is, its length and diameter, provides the basis for interpreting signaling in the cilium differently to the cell body (Truong *et al*, 2021). The model illustrates that the greater surface to volume ratio of the cilium compared to the cell body can account for a more efficient activation of a ciliary cAMP effector protein by ciliary cAMP than by non‐ciliary cAMP, even if the transduced information is diffusible and the domains are not segregated by a physical barrier (Truong *et al*, 2021). Our present study extends this model by including PDE4‐mediated compartmentalization of cAMP signaling, which functionally separats the ciliary cAMP compartment from the cytoplasm and provides the specificity to control renal cystogenesis. This multi‐layered compartmentalization is in line with previous reports demonstrating that in renal epithelial cells, PDE4 regulates cAMP‐stimulated cystogenesis (Omar *et al*, 2019) and PDE4C co‐localizes with the PC2/AKAP150 ciliary protein complex, which is dysregulated in ADPKD (Choi *et al*, 2011). The compartmentalization of cAMP signaling is a well‐established paradigm for how multiple signaling events are independently sensed and interpreted by the cell (Houslay, 2010; Musheshe *et al*, 2018). The cAMP PDEs represent the only means, by which intracellular cAMP is degraded, and as such, the localization patterns of cAMP‐PDEs are poised to compartmentalize cAMP signaling into discreet nanodomains and underpin compartmentalized cAMP signaling dynamics in living cells (Zaccolo, 2006; Baillie, 2009; Houslay, 2010; Henderson *et al*, 2014; Bobin *et al*, 2016; Zaccolo *et al*, 2021). Our study proposes that this principle not only applies to subcellular compartments within the cell body, but also applies to the primary cilium. This might be just the starting point for the identification of novel components that shape the ciliary signalosome and allow to process information separately from the rest of the cell.

In addition to controlling specific gene expression programs, our results revealed that ciliary cAMP signaling activates downstream mTOR signaling to drive cell proliferation. The mTOR signaling cascade has been shown to play an important role during ADPKD disease progression downstream of PC‐1 (Carrillo *et al*, 1982; Tao *et al*, 2005; Shillingford *et al*, 2006). So far, a link between ciliary cAMP signaling and mTOR signaling has not been established. Other studies have shown that the liver kinase B1 (LKB1) is localized to primary cilia (Mick *et al*, 2015), in particular to the primary cilia base, where LKB1 transduces the sensory input of flow into a change in mTOR signaling, indicated by a change in pS6RP levels (Boehlke *et al*, 2010). In fact, this physiological stimulus, flow sensed by primary cilia, decreased pS6RP levels (Boehlke *et al*, 2010). Here, we demonstrate that chronic stimulation of ciliary cAMP signaling increases pS6RP levels and, thereby, mTOR signaling. Thus, ciliary cAMP signaling seems to counteract LKB1 regulation of mTOR signaling. This nicely fits into a model, in which flow sensing in the ADPKD background is disturbed, leading to a dysregulation of second messenger dynamics: due to mutations in PC1/PC2, the Ca^2+^ homeostasis is disturbed and Ca^2+^ influx reduced, which would result in chronic activation of the ciliary localized, Ca^2+^‐inhibited AC5 and AC6 and, in turn, an increase in ciliary cAMP levels (Choi *et al*, 2011; Sussman *et al*, 2020). According to our data, this increase activates mTOR signaling and contributes to the proliferative drive during cyst development.

Using the mIMCD‐3 *in vitro* cell model, we extend the knowledge about the role of ciliary cAMP signaling in renal cystogenesis, providing a relevant cellular model for cAMP‐dependent cyst growth. Future work will explore the impact of ciliary cAMP signaling on *in vivo* cystogenesis. This will be possible when a suitable *in vivo* model that mimics ADPKD development and progression has been developed. To date, there is considerable variability of disease presentation within *in vivo* experiments and between different *in vivo* models (Guay‐Woodford, 2003; Nagao *et al*, 2012; Menezes & Germino, 2013; Woo *et al*, 2016). Similarly, to introduce optogenetics in this system to manipulate or rescue cellular signaling pathways with spatial resolution relies on new technical developments in intravital imaging in the kidney. Although intravital imaging in the kidney is feasible (Revell & Yoder, 2019), two‐photon excitation for many optogenetic tools is limited and requires an indirect approach or one‐photon excitation using a light fiber. However, we are confident that novel technical developments in the next few years will allow to apply our optogenetic approach to study PKD development *in vivo*.

In summary, our work reveals a novel concept of how the primary cilium controls certain cellular functions and maintains tissue integrity in a specific and spatially distinct manner. We have uncovered potential molecular targets that function in a compartmentalized manner, which may support the development of new therapeutic approaches for the treatment of PKD.

## Material and Methods

### Plasmids

All primer sequences used for cloning and the corresponding plasmids are summarized in Appendix Table S1. In brief, the cloning for the new constructs was done as follows: The plasmid pc3.1‐mNPHP3(201)‐bPAC‐mCherry has been described elsewhere (Hansen *et al*, 2020). The pc3.1‐mNPHP3(201)_Pink‐Flamindo plasmid is based on the addgene plasmid #102356. The Pink Flamindo sequence has been amplified using C4389/90 and subcloned into the pcDNA3.1(+) vector together with the mNPHP3(201) sequence described before (Hansen *et al*, 2020). The pEGFP‐N1‐bPAC plasmid has been cloned by amplifying bPAC from pc3.1‐bPAC‐mCherry (Hansen *et al*, 2020) using C4443/44 and subclone into pEGFP‐N1 (Clontech). The pEGFP‐N1‐mNPHP3(201) plasmid has been cloned by replacing the NheI/AgeI fragment (0.54 kbp) in pEGFP‐N1‐bPAC with the NheI/AgeI fragment (1.2 kbp) from pc3.1‐mNPHP3(201)‐bPAC‐mCherry. The pRRL‐pUbC‐mNPHP3‐bPAC‐mCherry‐HA plasmid is based on the pRRL‐pUbC backbone, and the insert was amplified from pc3.1‐mNPHP3(201)‐bPAC‐mCherry (see above) using W0138/40 (1^st^ PCR) and W0138/39 (2^nd^ PCR) and then cloned into the pRRL‐pUbC backbone using AQUA cloning (Beyer *et al*, 2015). The pRRL‐pUbC backbone, a customized lentiviral vector on the basis of pRRL (Dull *et al*, 1998) for expression controlled by the Ubiquitin C promoter, was a kind gift of Florian I. Schmidt, University of Bonn.

### Cell lines and tissue culture

mIMCD‐3 (CRL‐2123) and HEK293T (CRL‐3216) cells were obtained and authenticated from American Type Culture Collection (ATCC). All cells have been tested and are free from mycoplasma and other microorganisms.

The following stable mIMCD‐3 cell lines have been used. The cyto‐bPAC cells have been already described (Hansen *et al*, 2020). Cells expressing cilia‐bPAC have been transfected with pc3.1‐mNPHP3(201)‐bPAC‐mCherry (Hansen *et al*, 2020) and selected with 1.2 mg/ml Geneticin™ (G418 Sulfate, Gibco, #11811031) until cell clones could be picked, analyzed, and maintained as monoclonal cell lines. *Ift27^−/−^* cells have been described previously (Liew *et al*, 2014). *Ift27^−/−^*/cilia‐bPAC cells were generated by transducing *Ift27^−/−^* with a lentivirus expressing cilia‐bPAC (pRRL‐pUbC‐mNPHP3‐bPAC‐mCherry‐HA) as described below. Cells were splitted thrice before FACS and positive cells were selected according to their mCherry fluorescence—the gate was defined prior to FACS by measuring the fluorescence of cilia‐bPAC cells described above. Cilia‐PKI cells have been generated by inserting the mNPHP3(201)‐GFP‐PKI transgene into the Flp‐In locus of a mIMCD‐3 Flp‐In line using FRT recombinase (Mick *et al*, 2015). Cilia‐PKI/cilia‐bPAC cells were generated as described for the *Ift27^−/−^*/cilia‐bPAC cells. Flp‐in/cilia‐bPAC cells, used as control cells for cilia‐PKI/cilia‐bPAC and *Ift27^−/−^*/cilia‐bPAC cells, were generated as described for the *Ift27^−/−^*/cilia‐bPAC cells.

mIMCD‐3 were maintained in DMEM/F12 (1:1) medium, supplemented with GlutaMax (Gibco, #31331‐028) and 10% FCS at 37°C and 5% CO2, HEK293 cells were maintained in MEM containing non‐essential amino acids (Gibco, #11140‐035) and 10% FCS. Additionally, for stable cell lines generated by selection with Geneticin™, the following antibiotics were added: 0.8 mg/ml Geneticin™ (G418 Sulfate, Gibco, #11811031).

Generation of *Ift20^−/−^* mIMCD‐3 cells: Guide RNAs were selected from Brie library (Doench *et al*, 2016) and corresponding oligonucleotides were cloned into BL245. BL245 is similar to lentiCRISPR v2 Puro (gift from Feng Zhang, Addgene plasmid # 52961) (Sanjana *et al*, 2014) except for a proline to serine mutation in the puromycin N‐acetyl‐transferase gene, which increases its resistance to puromycin. The vectors were packaged into lentiviral particles and transfected into mIMCD‐3 cells. After selection, the pools were single‐cell sorted into 96‐well plates. Single mutant clones were identified with Sanger sequencing, immunofluorescence, and immunoblotting.

For 3D culture, a 24‐well plate (Greiner CELLSTAR, #665180) was equipped with 400 µl/well of medium and a Millicell^®^ Cell Culture Insert (Merck Millipore Ltd., #PICM01250). Per condition, 1.6 × 10^5^ cells in 40 µl of medium were mixed 1:2 with a 1:1 mixture of collagen I from rat‐tail (Gibco, #A10438‐01) and Matrigel (Geltrex™, Gibco, #A14132‐02) or Cultrex Basement Membrane Extract, Type 2 (R&D Systems, lot 1625951) on ice. The resulting gel was transferred into the prepared inserts (120 µl/insert), and gels were solidified in the incubator (37°C, 5% CO2). Afterwards (> 30 min post gel seeding), the growth medium below the filter inserts was exchanged to fresh medium including the respective pharmacological substances.

### Transfection

mIMCD‐3 cells were transfected with Lipofectamine 2000 (Thermo Fisher Scientific) according to the manufacturer’s protocol. The transfection medium was replaced after 4–5 h with full medium.

### Generation of lentivirus and transduction

HEK293T cells were transfected with the plasmids encoding *gag‐pol* (psPAX2), VSV‐G(pMD2 G), and the plasmid containing the gene of interest (pRRL‐pUbC‐mNPHP3‐bPAC‐mCherry‐HA) using GeneJuice (Merck, #70967) according to the manufacturer’s instructions. The medium was exchanged to 30% FCS medium after 24 h. Two days post transfection, the supernatant was harvested and sterile‐filtered with a 0.45 μm filter (Merck, #SLHP033RS). Target cells were transduced by overnight exposure to a 1:2 mix of the harvested, filtered supernatant and growth medium, supplemented with 4 µg/ml of Polybrene (Sigma Aldrich, #H9268).

### Immunocytochemistry

Immunocytochemistry was performed according to standard protocols. Cells were seeded on poly‐L‐lysine (PLL, 0.1 mg/ml, Sigma Aldrich, #P1399‐100MG)‐coated 13 mm glass coverslips (VWR) in a 4‐well dish (VWR). The medium was replaced with starvation medium (0.5% FCS) on the next day to induce ciliogenesis. Cells were fixed 48 h after inducing ciliogenesis (mIMCD‐3) with 4% paraformaldehyde (Alfa Aesar, ThermoFisher Scientific, #43368) for 10 min at room temperature. After washing thrice with PBS, cells were blocked with CT (0.5% Triton X‐100 (Sigma Aldrich, #X100) and 5% ChemiBLOCKER (Merck Millipore, #2170) in 0.1 M NaP, pH 7.0) for 30 min at room temperature. Primary and secondary antibodies were diluted in CT and incubated for 60 min each at room temperature, respectively. Coverslips were mounted with one drop of Aqua‐Poly/Mount (Tebu‐Bio, #07918606‐20). The following primary antibodies were used as follows: mouse anti‐ARL13B (1:600, Abcam, ab136648), rabbit anti‐ARL13B (1:600, ProteinTech, #17711‐1‐AP), rabbit anti‐phospho‐CREB (Ser133) (87G3, 1:800, Cell Signaling, #9198), mouse anti‐ZO‐1 (1A12 (1:300, Thermo Fisher Scientific, # 33‐9100)), rat anti‐Ki‐67 (1:500, Thermo Fisher Scientific, #14‐5698‐82), mouse‐anti‐γ Tubulin (1:2000, SigmaAldrich, #T6557), mouse‐anti‐EP4 (1:100, Santa Cruz, sc‐55596), goat‐anti‐PDE4 long isoform antibodies (1:500) (Omar *et al*, 2019). As a DNA counterstain, DAPI was used (4',6‐Diamidino‐2‐Phenylindole, Dihydrochloride, 1:10.000, Thermo Fisher Scientific, #D1306).

The following secondary antibodies were used as follows: goat‐anti‐mouse‐Alexa488 (1:400, Thermo Fisher Scientific, #A11029), donkey‐anti‐mouse‐Alexa647 (1:700 Thermo Fisher Scientific, #A31571), goat‐anti‐rabbit‐Alexa488 (1:500, Thermo Fisher Scientific, #A11034), goat‐anti‐rabbit‐Alexa647 (1:500, Thermo Fisher Scientific, #A21245), donkey‐anti‐rat‐Alexa488 (1:500, Dianova, #712‐545‐153), donkey‐anti‐goat‐Alexa647 (1:500, Life Technologies, #A21447), donkey‐anti‐mouse‐Alexa488 (1:400, Jackson, #715‐545‐150).

At the end of the experiment, 3D cultures were washed with PBS (all applied below the filter inserts), fixed with 4% paraformaldehyde in PBS for 30 min (Alfa Aesar, ThermoFisher Scientific, #43368), and washed thrice with PBS.

For staining, 3D gels were removed from the cell culture inserts with a small spatula and transferred to a 24‐well plates. All following steps were performed at RT if not stated otherwise. Gels were washed twice with PBS and incubated thrice in PBS/1% Triton X‐100 (Sigma Aldrich, #X100) for 10 min. Next, gels were blocked with PBS/1% Triton X‐100/10% FCS for 1 h and incubated over night with the primary antibodies in PBS/1% Triton X‐100/10% FCS at 4°C. Afterward, gels were washed thrice with PBS/1% Triton X‐100/10% FCS for 20 min and PBS/1% Triton X‐100 for 10 min. Gels were incubated with the secondary antibodies and DAPI in PBS/1% Triton X‐100/10% FCS for 5 h at RT. Afterward, gels were washed thrice with PBS/1% Triton X‐100 for 5 min, washed once with PBS, and mounted on cover slips with Aqua‐Poly/Mount (Tebu‐Bio, #07918606‐20) using a custom mounting procedure to avoid pressure on the gels during mounting. The cover slips were placed on a firm surface and equipped with custom‐build spacers on the sides (three layers of adhesive tape) and drops of PBS at the positions where gels were mounted. Gels were transferred to the PBS drops using a small spatula, PBS was removed from around the gels, and Aqua‐Poly/Mount was added onto and around the gels. The object slide was approached from the top until the cover slips with the gels slightly adhered to it. Object slides were then dried upside down in a hanging position avoiding pressure on the gels for at least 3 h at RT. Antibodies were applied as described under immunocytochemistry.

### Optogenetic stimulation

For optogenetic stimulation in 2D and 3D cell culture, an RGB LED panel was used (30 x 30cm, 18 W, Lichtblick GmbH, Germany, #P1327). Only the blue LEDs were switched on for all stimulations and measurements. A wavelength spectrum from 240–866 nm was measured (Ocean optics, S1024 DW) and showed for the blue LEDs only one peak at 465 nm. The power at the surface of the LED plate was 38.8 µW/cm², determined using a power sensor (Coherent, PS19 Q).

### Confocal microscopy and image analysis

Confocal z‐stacks were recorded with a confocal microscope at the Microscopy Core Facility of the Medical Faculty at the University of Bonn (Leica Sp8 or Sp5; imaging of labeled cells cultured in 2D: z‐step size 0.5 µm, 63× oil‐immersion objective; imaging of stained 3D cultures: z‐step size 0.88 µm, 20× multi‐immersion objective applied with glycerol immersion). All depicted images show a maximum projection of a z‐stack unless differently stated in the figure legend. For quantifying fluorescence signals, z‐stacks were recorded from at least two random positions per experiment and analyzed using “CiliaQ” (Hansen *et al*, 2021). We previously developed CiliaQ to fully automatically quantify the ciliary intensity levels in the different channels. CiliaQ detects individual 3D objects in the segmented channel and filters out 3D objects below a pre‐defined size threshold (30 voxel) to exclude noise. Each remaining 3D object is considered as a cilium. For each cilium, CiliaQ quantifies the pixels belonging to the 3D region in each channel. All results were scrutinized by a trained observer. In all plots, the parameter revealing the average intensity of the 10% of cilia pixels with highest intensity is shown as ciliary intensity level. From all values, the background intensity level was subtracted. All analyses have been performed blind to the condition.

### Proliferation assay

Cells were seeded on 96‐well plates (PerkinElmer, #6055302) and grown for 3 d in the dark or stimulated by light (1‐h light/1‐h dark, 465 nm, 38.8 µW/cm²). Afterward, cells were fixed, labeled, and images were acquired using the Zeiss Celldiscoverer 7 (20× objective, 0.5× magnification changer, resolution 0.9 µm/px, 6 images acquired per well, z‐stack acquired consisting of 7 planes with an inter‐plane distance of 3 µm). Images were quantified in ImageJ using the freely accessible ImageJ plugins ExtractSharpestPlane_JNH (available through GitHub at and archived on zenodo (https://doi.org/10.5281/zenodo.5646492), and AdipoQ Preparator and AdipoQ Analyzer (available on GitHub at https://github.com/hansenjn/AdipoQ) (Sieckmann *et al*, 2022) to automatize the workflow. Briefly, the sharpest plane of the z‐stack was determined by the highest pixel variance. A maximum projection of the sharpest plane and the plane above and below was created, which was used for quantification. Next, the DAPI channel of this image was segmented into fore‐ and background as follows: (i) The channel images was filtered with a Gaussian blur (sigma 2 px). (ii) A copy of the blurred channel image was created that was additionally filtered with a Gaussian blur (sigma 3 px). (ii) The copied blurred channel image created in (ii) was subtracted from the image created in (i). (iv) The resulting image was finally segmented based on an intensity threshold defined by the Triangle algorithm, implemented in ImageJ. Fused nuclei were separated using the Watershed algorithm implemented in ImageJ. The segmented, watershed image was then scrutinized by a trained, blinded observer and small segmentation errors (e.g., nucleus split into multiple parts or adjacent nuclei still fused after watershed) were manually corrected. Next, the segmented channel image was used to determine a region of interest (ROIs) for each nucleus using ImageJs wand tool. ROIs enclosing an area less than 20 px were excluded. For all remaining ROIs, the median pixel intensity was determined. If the median pixel intensity exceeded a certain threshold, cells were considered “high,” and otherwise, “low.” The threshold was determined for each experiment and cell line individually as the median of all median intensities across all respective images + 1200. All analyses have been performed blind to the condition.

### Cyst analysis

Images were taken using a Zeiss Stemi binocular microscope, equipped with an RGB camera at 1× and 5× magnification. Images from a 1× magnification reveal the entire 3D gel and were analyzed manually in ImageJ by a blinded, trained observer (Appendix Fig S1). A region‐of‐interest (ROI) was drawn for each cyst in the image to obtain the area. The cyst number represents the number of ROIs per image. The cyst area was obtained by summing up the areas of all drawn ROIs. For images with very high cyst count (e.g., Forskolin treatment), only one‐quarter of the gel was analyzed, and final values were inferred by multiplying the results for the quarter by four. All analyses have been analyzed blind to the condition.

### Live cell imaging

#### Primary cilia

For imaging the PGE2‐induced ciliary cAMP increase, cells were seeded on CellCarrier‐96 Ultra Microplates (3 × 10^4^ cells per well, PerkinElmer, #6055302). After 24 h, cells were transduced with the ratiometric cilia‐targeted cADDis cAMP assay kit (5‐HT6‐mCherry‐cADDis, Montana Molecular, #DG0211G). 2.5 µl of the BacMAM concentrated stock was mixed with 0.4 µl sodium butyrate (500 mM, Sigma Aldrich, #B5887), and 97.1 µl OptiMEM (ThermoFischer Scientific, #11058‐021). The growth medium was exchanged with the 100 µl transduction mix and incubated for 30 min at RT, before further incubation for 6–8 h at 37°C, 5% CO_2_. Subsequently, the transduction mix was exchanged to starvation medium and further incubated at 37°C overnight. For imaging ciliary cAMP levels, cilia‐bPAC or cyto‐bPAC cells were seeded as described above. After 24 h, cells were transfected with pc3.1‐mNPHP3(201)_pink‐Flamindo in combination with cilia‐bPAC (pEGFP‐N1‐mNPHP3(201)) or cyto‐bPAC (pEGFP‐N1‐bPAC) and handled under dim red light. 24 h post transfection, the growth medium was replaced with 100 µl starvation medium per well. 3–4 h before imaging, cells were treated with 500 nM SiR‐tubulin (SPIROCHROME, #SC002) according to the manufacture’s protocol. Cells were either imaged as described before (Forskolin) (Hansen *et al*, 2020) or imaged at the Microscopy Core Facility of the Medical Faculty at the University of Bonn using the Visitron VisiScope Spinning Disk Setup (Build on a Zeiss Axio Observer, Zeiss) at 37˚C. The mNPHP3(201)‐mCherry‐cADDis and mNPHP3(201)_Pink‐Flamindo were measured with the same settings, except for individual laser intensities. The mNPHP3(201)‐mCherry‐cADDis was excited with the 448 nm (5%) and 561 nm (5%) laser, while mNPHP3(201)_Pink‐Flamindo together with cilia‐bPAC or cyto‐bPAC was excited with the 448 nm (5%) and 561 nm (20%) laser. Both biosensors were measured in combination with the 405‐488‐560‐640bs dichroic mirror in the Yokogawa CSU‐W1 unit and the 50 nm pinhole disk at 4,000 rpm disk speed. A 60× C‐Apochromat water objective (NA = 1.2) was used. Images were acquired on two pco.edge sCMOS cameras simultaneously (200 ms exposure time, 2× binning) with a GFP/RFP emission filter cube for measuring fluorescence in the GFP and RFP channel, respectively, at 500 nm step size. Before measuring, cells were washed once, and the medium replaced with extracellular solution (ES buffer: 120 mM NaCl, 5 mM KCl, 2 mM CaCl_2_, 10 mM Glucose, 10 mM HEPES, pH 7.4) and cilia were identified using the SiR‐tubulin fluorescence in the Cy5 channel (640 nm laser at 5% intensity, 700‐75m emission filter in combination with 405‐488‐560‐640bs dichroic mirror in the Yokogawa CSU‐W1 unit and the 50 nm pinhole disk at 4,000 RPM disk speed). The experimental procedure for measuring mNPHP3(201)‐mCherry‐cADDis was as follows: after a stable baseline was obtained, cells were stimulated with 50 nM PGE2. The experimental procedure for measuring mNPHP3(201)_Pink‐Flamindo was as follows: For three time steps (10 s per step), cells were imagined in the RFP and Cy5 channel to reveal cilia based on the SiR tubulin dye and then, cells were measured in the GFP and RFP channel for six additional time steps (10 s per step). Images were analyzed using CiliaQ (Hansen *et al*, 2021). In all plots, the parameter revealing the average intensity of the 10% of cilia pixels with highest intensity is shown as ciliary intensity level. All analyses have been performed blind to the condition.

### Cytoplasmic cAMP imaging

For measurement of somatic cAMP levels in wild‐type (WT) mIMCD‐3 cells, cells were seeded on CellCarrier‐96 Ultra Microplates (3 × 10^4^ cells per well) PerkinElmer, #6055302. After 24 h, cells were transduced with Green Down cADDis cAMP assay kit (Montana Molecular, #D0200G). 12.5 µl of the BacMAM was mixed with 0.4 µl sodium butyrate (500 mM, Sigma Aldrich, #B5887), and 87.1 µl OptiMEM (ThermoFischer Scientific, #11058‐021) and incubated for 30 min at RT. The growth medium was exchanged for the 100 µl transduction mix and cells were incubated with the mix for 6‐8 h at 37°C, 5% CO_2_. Subsequently, the transduction mix was exchanged to starvation medium containing 0.4 µl sodium butyrate and further incubated at 37°C overnight. The next day, cells were imaged at the Microscopy Core Facility of the Medical Faculty at the University of Bonn using the Zeiss Observer Z1 (Zeiss) at 37˚C. For that, cells were washed once and then imaged in ES buffer using the Filter Set 38 HE and 250 ms exposure time. Images were taken every 10 s for 5.5 min. After 1 min, cells were stimulated with 50 nM PGE2 and measurement was continued for 3 min. For the measurement of somatic cAMP levels in mIMCD‐3 cilia‐bPAC cells, cells were prepared as described for WT cells but imaged at the setup described for imaging of primary cilia (see above).

### ELISA‐based cAMP measurements

Total cAMP levels were determined using a CatchPoint™ assay (Molecular Devices, #R8089) according to the manufacturer’s instructions. mIMCD‐3 cells were seeded on a PLL (0.1 mg/ml, Sigma Aldrich, #P1399‐100MG)‐coated 96‐well plates (F‐Bottom, CELLSTAR, Greiner) at 1.8 × 10^4^ cells/ well and incubated over night at 37°C and 5% CO_2_ in the dark. During all further experimental procedures, cells or cell lysates were kept in the dark and handled only under dim red light, preventing bPAC activation. After 24 h, the medium was changed to starvation medium to induce ciliogenesis. Another 47 h later, the medium was replaced with ES and cells were either subjected to a 1 h light pulse (465 nm, 38.8 µW/cm²) or kept in the dark. Alternatively, WT cells were exposed to Forskolin 10 µM or DMSO for 1 h. Directly after stimulation, cells were lysed and cAMP amounts per well were determined. The protein concentration was determined with a Pierce™ BCA Protein Assay Kit (Thermo Fisher Scientific, # 23227) according to the manufacturer’s instructions.

### Protein preparation and Western blot analysis

All steps were performed at 4°C and in the presence of a mammalian protease inhibitor cocktail (mPIC, Sigma Aldrich, #P8340‐1ML) and phosphatase inhibitor cocktail (PhosSTOP™, Roche, #04 906 845 001). Cells were washed with PBS and lysed in the plates using RIPA buffer (20 mM Tris‐HCl pH 7.4, 150 mM NaCl, 1 mM EDTA, 1% Triton X‐100, 10% glycerol, 0.1% SDS, and 0.5% deoxycholate). The protein concentration was determined with a Pierce™ BCA Protein Assay Kit (Thermo Fisher Scientific, #23227) according to manufacturer’s instructions.

Samples were suspended in SDS sample buffer (200 mM Tris/HCl pH 6.8, 8% SDS, 4% ß‐mercapto‐ethanol, 50% Glycerin, 0.04% bromphenol blue) and separated using gradient gels (TruPAGE™, Sigma Aldrich, #PCG2003‐10EA). Electrophoresis was performed in MOPS running buffer (Sigma Aldrich, #PCG3003‐500 ml) in an XCell SureLock Mini‐Cell (Thermo Fisher Scientific, #EI0001) at 120 mA and 180 V. As protein standard, Protein marker VI (10‐245 kDa, AppliChem, #A8889) was used. Gels were blotted to an Immobilon FL membrane (Merck, #IPFL00010) for 50 min at 172 mA per gel in a semi‐Dry‐Blotter MAXI (Carl Roth #T788.1). Before and after blotting, the membrane was shortly activated in methanol. Membranes were blocked with a 1:1 mixture of Intercept™ Blocking Buffer (in PBS, LI‐COR Biosciences, #927‐70001) and PBS supplemented with Tween^®^ 20 (Sigma, #P9416) or Intercept™ Blocking Buffer (in TBS, LI‐COR Biosciences, #927‐60001) and TBS supplemented with Tween^®^ 20 (Sigma, #P9416). Antibodies were diluted in the same mixture for incubation. The following primary antibodies were used as follows: mouse anti‐S6RP (54D2, 1:1,500, Cell Signaling, #2317), rabbit anti‐phospho‐S6RP (Ser^235/236^, 1:3,000, Cell Signaling, #4858), mouse anti‐beta‐Tubulin, (1:5,000, Sigma Aldrich, #T4026), goat‐anti‐PDE4 long isoform antibodies (1:2,000) (Omar *et al*, 2019). The following secondary antibodies were used: donkey anti‐rabbit IRDye 680RD (LI‐COR Biosciences, #926‐68073), donkey anti‐mouse IRDye 800CW (LI‐COR Biosciences, #926‐32212), donkey anti‐goat HRP (Dianova, 705‐035‐147).

Images were taken on LI‐COR Odyssey imaging system (for IRDye detection) or a Sapphire imaging system (for HRP detection). Protein expression was quantified using ImageJ (version 1.48, Wayne Rasband, National Institutes of Health, USA).

### RNA‐sequencing and data analysis

Cells were seeded in triplicates in 6‐well plates (2.46 × 10^5^ cells/well, Greiner CELLSTAR, #657160) and starved after 24 h for 47 h. Cells were stimulated by light (1 h, 465 nm, 38.8 µW/cm²) or kept in the dark. Control mIMCD‐3 cells were stimulated with DMSO or 10 μM Forskolin for 1 h.

Cells were washed ones with PBS and next directly lysed from the plates by addition of 400 µl/well of Trizol (Thermo Fisher Scientific, #15596026). Triplicates were pooled into one tube. RNA was extracted using the miRNeasy micro kit (Qiagen). Total RNA quantity and quality were determined via RNA assay on a Tapestation 4200 system (Agilent). 5 ng total RNA were used as an input for NGS library preparation following the SMART‐Seq2 protocol (Picelli *et al*, 2014). Final libraries were quantified by HS dsDNA assay on a Qubit (Invitrogen) and fragment size distribution was checked via D5000 assay on a Tapestation 4200 system (Agilent). Libraries were equimolarly pooled and clustered at 1.4 pM and sequenced SR 75 cycles on a NextSeq500 instrument with High Output v2 chemistry (Illumina) to ~ 10 million raw reads per sample. Sequencing data was demultiplexed and converted into fastq format using bcl2fastq2 v2.20. For each condition, four independent experiments were performed and included into the data analysis. Kallisto (Bray *et al*, 2016) was used to quantify abundances of transcripts from RNA‐seq data. Raw kallisto files were imported using tximport (Soneson *et al*, 2015) and the function DESeqDataSetFromTximport from DESeq2 (Love *et al*, 2014) and transformed in DESeq2 using variance stabilizing transformation. DESeq2 was used for the calculation of normalized counts for each transcript using default parameters. To exclude transcripts with low abundance, transcripts with less than 40 counts in all samples were excluded. This filtering revealed 22444 present transcripts. The model matrix for Deseq2 was generated based on the design ~Experimentday+Celltype+Celltype:Treatment and coefficients for comparisons that did not exist in the study design were excluded from the matrix (“cyto‐bPAC:DMSO,” “cilia‐bPAC:DMSO,” “cyto‐bPAC:Forskolin,” and “cilia‐bPAC:Forskolin”). Differentially expressed genes were calculated separately for each cell line and each contrast (dark vs. light for all cells, and DMSO vs. Forskolin in WT cells) using an adjusted *P*‐value cutoff of 0.05 and are listed in Dataset EV1. Log‐fold change shrinkage was performed using the apeglm package (Zhu *et al*, 2019). Reactomes were revealed using the online tool reactome.org (Jassal *et al*, 2020). Transcription factor enrichment analysis was performed using the package pcaGoPromoter (Hansen *et al*, 2012).

### Quantitative real‐time PCR

Cells were seeded and treated as described for the RNA sequencing. Cell lysis and RNA extraction was performed using the RNA preparation kit (PureLink™ RNA Mini Kit, Thermo Fisher Scientific, #12183018A) according to the manufacturer’s protocol. The RNA concentration was determined using the NanoDrop (Thermo Fisher Scientific) and the RNA quality was verified by gel electrophoresis. The reverse transcription was performed using 1 μg RNA (iScript™ cDNA Synthesis Kit, BIO‐RAD, #1708891) and qPCR was performed using the IQ™ SYBR^®^ Green Supermix (BIO‐RAD, #170‐8885) or PowerTrack™ SYBR Green Mastermix (Applied Biosystems, #A46109). The qPCR protocol was as follows: 1. Denaturation at 95°C for 180 s, 2. Denaturation at 95°C for 10 s, annealing at 59°C for 30 s, elongation at 72°C for 30 s, 45 cycles, melt curve. The primer sequences are listed in Appendix Table S2.

### Pharmacology

All compounds are listed in Appendix Table S3. MR‐L8, a PDE4 long isoform activator, is part of Mironid’s published patent application WO2019193342, Example 34.

### Software

Data analysis and statistical analysis was performed in Excel (Microsoft Office Professional Plus 2013, Microsoft), R (Version 3.6.2 (2019‐12‐12)), R Studio (RStudio, Inc., Version 1.2.5033), and GraphPad Prism (Version 8.1.2, GraphPad Software, Inc.). All image processing and analysis was performed in ImageJ (Version v1.52i, U.S. National Institutes of Health, Bethesda, Maryland, USA). Plots and Figures were generated using GraphPad Prism (Version 8.1.2, GraphPad Software, Inc.), R (Version 3.6.2 (2019‐12‐12)), R Studio (RStudio, Inc., Version 1.2.5033), and Affinity Designer (Serif (Europe) Ltd., 2020, Version 1.9.3). The CiliaQ software has been described elsewhere (Hansen *et al*, 2021) – the following versions were applied: CiliaQ Preparator v0.1.1, CiliaQ Editor v0.0.2, CiliaQ v0.1.4.

## Author contributions

**Jan N Hansen:** Conceptualization; Data curation; Formal analysis; Supervision; Validation; Visualization; Methodology; Writing—review & editing; JNH and DW conceived and designed the study and wrote the manuscript; performed and supervised experiments and analyzed the data. **Fabian Kaiser:** Data curation; Formal analysis; Writing—review & editing; performed live‐cell cilia imaging, analyzed the data, and edited the manuscript. **Philipp Leyendecker:** Data curation; Formal analysis. **Birthe Stüven:** Data curation; Formal analysis. **Jens‐Henning Krause:** Data curation; Formal analysis. **Fatemeh Derakhshandeh:** Data curation; Formal analysis. **Jaazba Irfan:** Data curation. **Tommy J Sroka:** Data curation; Formal analysis. **Kenley M Preval:** Data curation; Formal analysis. **Paurav B Desai:** Data curation; Formal analysis. **Michael Kraut:** Data curation. **Heidi Theis:** Data curation. **Anna‐Dorothea Drews:** Formal analysis; Methodology. **Elena De‐Domenico:** Formal analysis; Methodology. **Kristian Händler:** Formal analysis; Methodology. **Gregory J Pazour:** Conceptualization; Resources; Formal analysis. **David JP Henderson:** Conceptualization; Resources; Formal analysis; Validation; Writing—review & editing. **David U Mick:** Conceptualization; Data curation; Formal analysis; Supervision; Methodology; Writing—review & editing. **Dagmar Wachten:** Conceptualization; Formal analysis; Supervision; Funding acquisition; Validation; Investigation; Visualization; Writing—original draft; Project administration; Writing—review & editing.

## Disclosure and competing interests statement

DJPH and JI are employees of Mironid Limited.

## Supporting information



Appendix

Expanded View Figures PDF

Dataset EV1

## Data Availability

RNA‐Sequencing data were deposited in GEO under the accession Number GSE182339 (https://www.ncbi.nlm.nih.gov/geo/query/acc.cgi?acc=GSE182339). To obtain new materials and reagents generated in this paper, please contact the corresponding author.
