# Supplementary material for: A cAMP signalosome in primary cilia drives gene expression and kidney cyst formation
Source: EMBO Rep. 2022 Jun 13;23(8):e54315. doi: 10.15252/embr.202154315 (PMC9346484; doi:10.15252/embr.202154315)
Supplement: Supplementary file 1 — Appendix [file EMBR-23-e54315-s001.pdf]

Appendix

Table of Content:

- Appendix Table S1-3
- Appendix Figure S1-2

Appendix Table S1

Plasmids and cloning information. Plasmids are listed and the ID and sequence of the primers that have been used for cloning are indicated.

**pc3.1-mNPHP3(201)_pink-Flamindo**		
**ID**	**Sequence 5'-3'**	**Primer Info**
C4389	TAGTGGATCCATGCTGGTGAGCAAG	5' primer for PCR Pink Flamindo
C4390	CTTATCTAGATTACTTGTACAGCTCG TCCATGCCG	3' primer for PCR Pink Flamindo
**pEGFP-N1-bPAC**		
**ID**	**Sequence**	**Primer Info**
C4443	AATCGCTAGCCACCATGAAGCGGCT GGTGTACATC	5' primer for PCR bPAC
C4444	GCCGAATTCTGTTCTTGTCGTTTTCC AGGGTCTGC	3' primer for PCR bPAC
**pRRL-pUbC-mNPHP3-bPAC-mCherry-HA**		
**ID**	**Sequence 5'-3'**	**Primer Info**
W0138	CCGTTTTTGGCTTTTTTGTTAGACGC GCCGGCGCCACCATGGGTACCGCCT	5' primer for PCR of mNPHP3-bPAC-mCherry-HA, 1st PCR
W0139	CGACGGCCAGTGAATTATGGATCCT TACGCATAATCCGGCACATC	3' primer for PCR mNPHP3-bPAC-mCherry-HA, 2nd PCR
W0140	CGACGGCCAGTGAATTATGGATCCT TAGGCGTAGTCGGGCAC	3' primer for PCR mCherry, adds HA, XbaI

Appendix Table S2

Primers used for qPCR. The sequence of the primers that have been used for qPCR are indicated.

ID	Sequence 5'-3'	Primer Info
C3206	AGGTCGGTGTGAACGGATTTG	Forward primer GAPDH, housekeeper 1
C3207	TGTAGACCATGTAGTTGAGGTCA	Reverse primer GAPDH, housekeeper 1
C2844	GCGTCTCCTTCGAGCTGTT	Forward primer PPIA, housekeeper 2
C2845	AAAGTCACCACCCTGGCA	Reverse primer PPIA, housekeeper 2
C3849	TGTGACTGTACCCGGACTGG	Forward primer PTGS2, gene of interest
C3850	TGCACATTGTAAGTAGGTGGAC	Reverse primer PTGS2, gene of interest

Appendix Table S3

Pharmacology and compounds. The table contains the pharmacology and compounds that have been used.

**Name**	**Stock concentration**	**Company, catalog number**
MR-L8	20 mM in DMSO	Mironid
Forskolin	20 mM in DMSO	Sigma-Aldrich, F6886-10MG
Ciliobrevin-D	50 mM in DMSO	Merck/Sigma-Aldrich, 250401
IBMX	250 mM in DMSO	AppliChem, A0695,0001
Rolipram	20 mM in DMSO	Sigma-Aldrich, R6520
PGE2	1 mM in Acetone	Sigma-Aldrich, P0409
Celecoxib	30 mM in DMSO	Sigma-Aldrich, PZ0008
AH6809	10 mM in DMSO	Sigma-Aldrich, A1221
L161,982	10 mM in DMSO	Sigma-Aldrich, SML0690
Rapamycin	10 μM in DMSO	Sigma-Aldrich, R0395

Appendix Figure S1

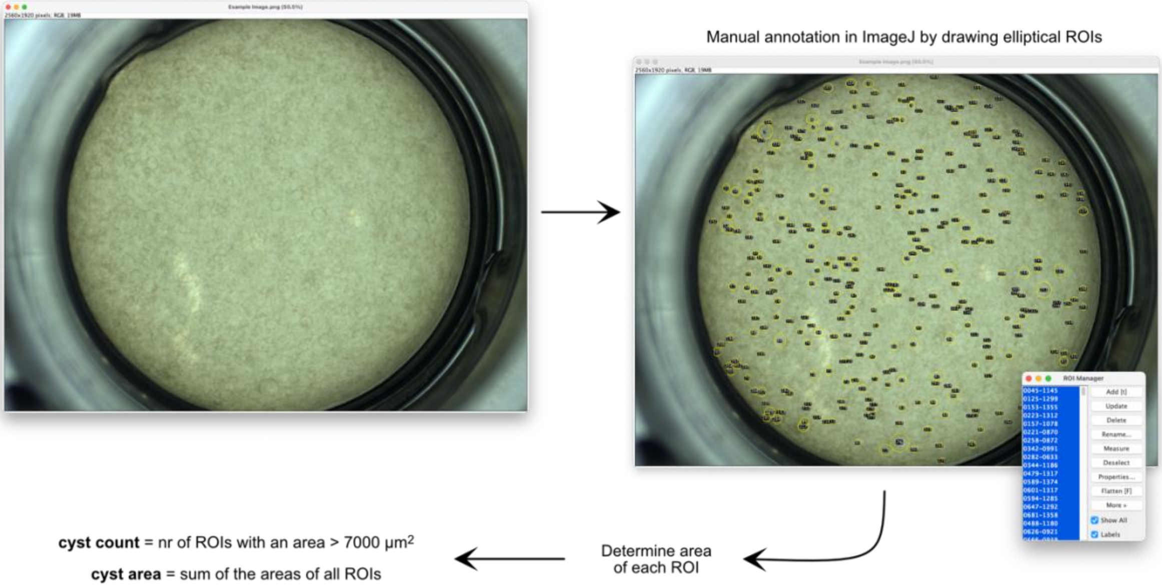

Appendix Figure S1: Work flow to identify and quantify cyst formation. Images were recorded with a Binocular microscope and then opened in FIJI / ImageJ. Using the elliptical ROI (region of interest) tool, a trained observer, blind to the condition, annotated all cysts and stored the annotations in the ROI Manager. Next, the area for each ROI in the ROI Manager was automatically determined using ImageJ's measure function. The cyst count was determined as the number of ROIs with an area > 7000 µm^2 (this threshold was introduced because we observed that annotation of very small cysts is difficult and those very small cysts readily slip out the attention of the observer). The area covered by cysts was determined as the sum of the areas of all ROIs. Only cysts with a clearly visible lumen were considered in quantifications and tiny cysts, which were difficult to distinguish from cellular agglomerates were not included in the quantifications.

Appendix Figure S2

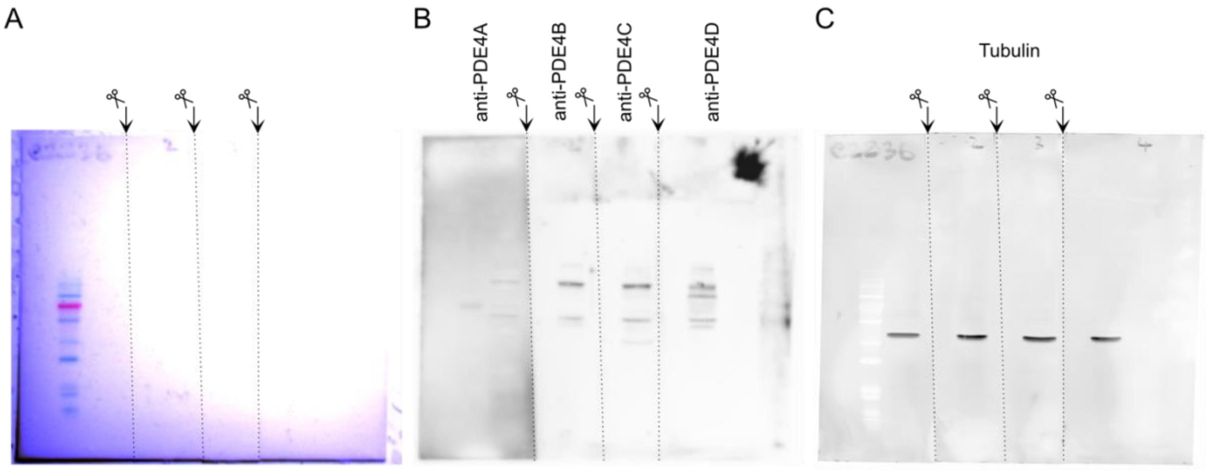

Appendix Figure S2: Original immunoblots. (A) Image of the membrane. Scissors indicate where the blot has been cut. **(B)** Analysis of Pde4 isoform expression (long and short isoforms) in wild-type mIMCD-3 cells, revealed by immunoblotting against Pde4A, Pde4B, Pde4D, and Pde4D. **(C)** Tubulin loading control.
