# Supplementary material for: A cAMP signalosome in primary cilia drives gene expression and kidney cyst formation
Source: EMBO Rep. 2022 Jun 13;23(8):e54315. doi: 10.15252/embr.202154315 (PMC9346484; doi:10.15252/embr.202154315)
Supplement: Supplementary file 2 — Expanded View Figures PDF [file EMBR-23-e54315-s003.pdf]

Expanded View Figures

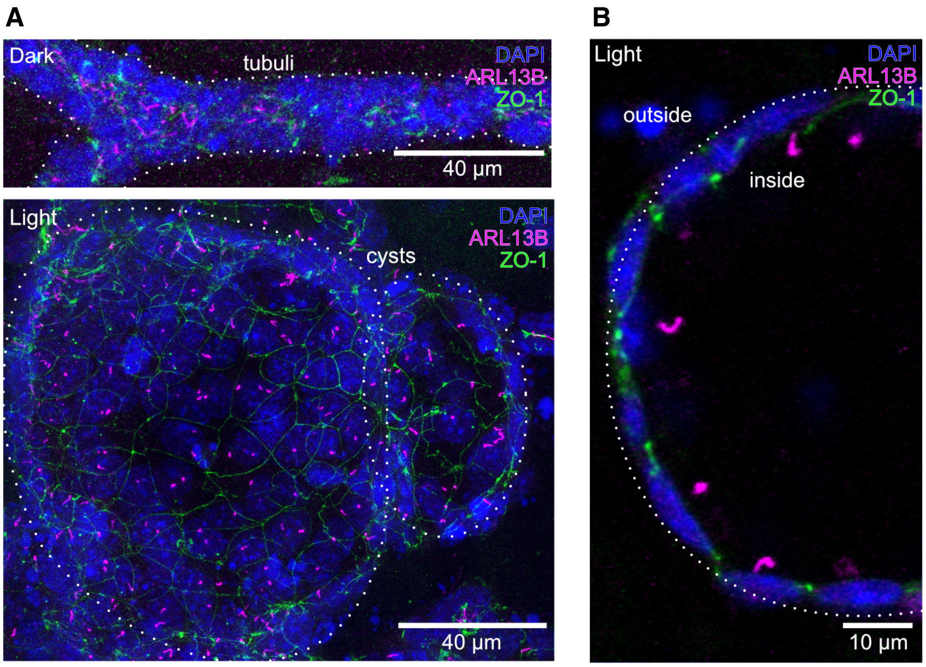

Figure EV1. Ciliary cAMP signaling drives cyst growth.

A, B Immunocytochemistry of mIMCD-3 cells stably expressing cilia-bPAC, cultured in a 3D matrix in the dark or during light exposure (1 h light/1 h dark, 9 days, 465 nm, 38.8 µW/cm²). Cells were labeled with DAPI (blue) to label the DNA, with an ARL13B antibody (magenta, ciliary marker) to label cilia, and a ZO-1 antibody (green) to label tight junctions of the epithelium. Scale bars are indicated. (A) Maximum intensity projection of a z-stack through the entire tubule (top) or cyst (bottom). (B) Projection of confocal slices acquired from a cyst. Scale bars are indicated.

Figure EV2. The role of PDE4 isoforms in ciliary cAMP-dependent cyst formation.

- A Analysis of PDE expression in wild-type mIMCD-3 cells, determined by unbiased RNA-Sequencing. PDEs specific for cAMP are indicated. Data are shown as mean ± SD. Data points show individual experiments.
- B mIMCD-3 cells stably expressing cyto-bPAC, cultured in a 3D matrix during light exposure (1 h light/1 h dark, 9 days, 465 nm, 38.8 µW/cm², started 1 day after pharmacological stimulus) and incubated with 250 µM IBMX. Exemplary image shown (*n* = 3 experiments). Scale bars are indicated. Matching 3D cultures, incubated with DMSO or 10 µM rolipram, are shown in Fig 4C.
- C Wild-type (WT) mIMCD-3 cells, cultured in a 3D matrix and incubated with DMSO (control), 250 µM IBMX, or 10 µM rolipram. Exemplary images are shown (*n* = 3-4 experiments). Scale bars are indicated.
- D Quantification of images exemplified in (C). Data are shown as mean ± SD, data points show individual experiments. *P*-values were calculated using a ratio-paired, two-sided Student's *t*-test.
- E 3D culture of mIMCD-3 cells stably expressing cilia-bPAC in the dark and incubated with increasing concentrations of the PDE4 long-isoform activator MR-L8. Exemplary images are shown (*n* = 3 independent experiments). Scale bars are indicated.
- F Quantification of images exemplified in (E). Data are shown as mean ± SD, data points show individual experiments.
- G 3D culture of wild-type mIMCD-3 cells incubated with 10 µM Forskolin and increasing concentrations of MR-L8. Exemplary images are shown (*n* = 3). Scale bars are indicated.
- H Quantification of images exemplified in (G). Data were normalized to 10 µM Forskolin/0 µM MR-L8 (set to 100%) and are shown as mean ± SD. Data points show individual experiments. *P*-values were calculated using a paired, two-sided Student's *t*-test.

 © 2022 The Authors

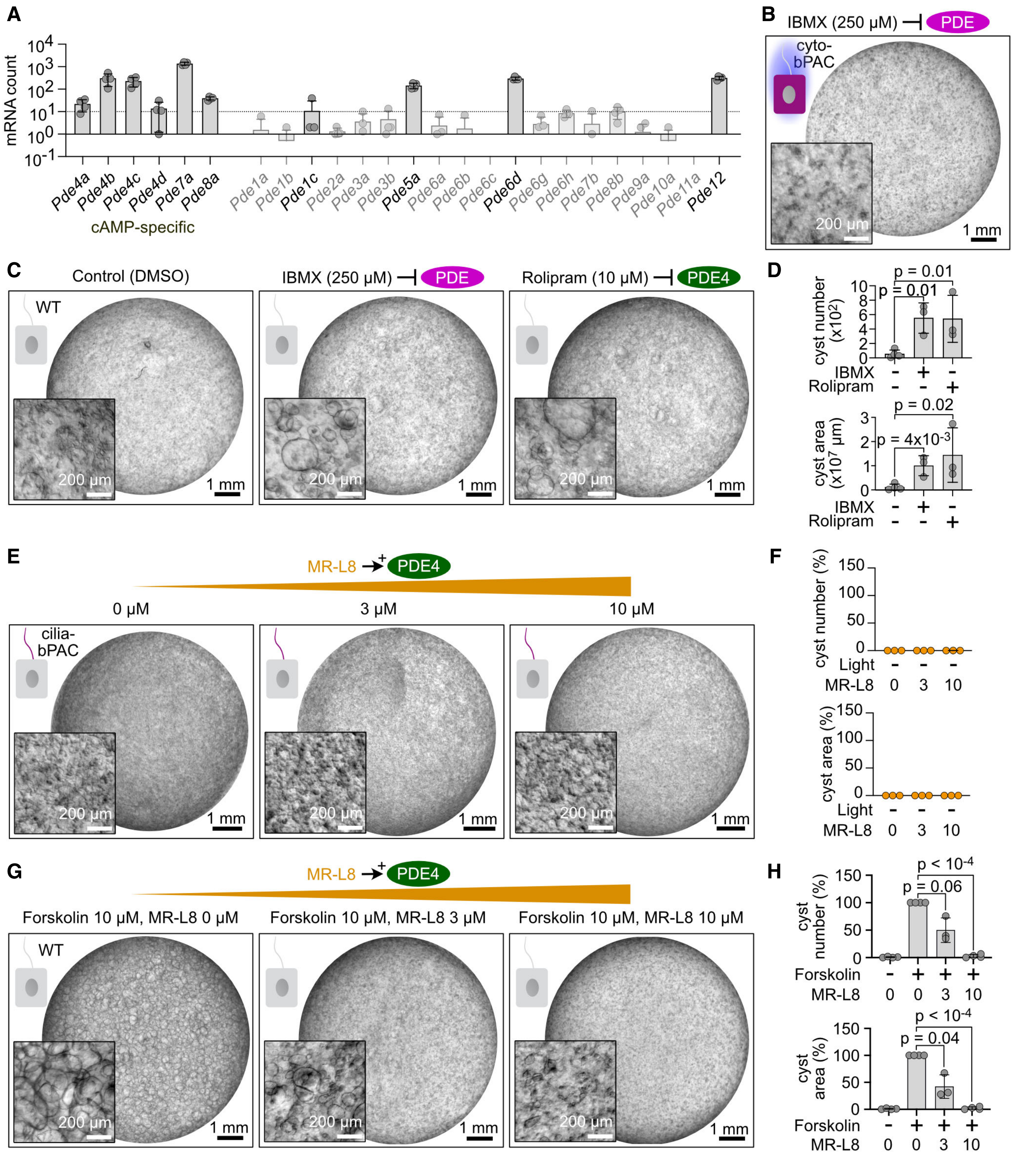
A
mRNA count
cAMP-specific
Pde4a Pde4b Pde4c Pde4d Pde7a Pde8a Pde1a Pde1b Pde1c Pde2a Pde3a Pde3b Pde5a Pde6a Pde6b Pde6c Pde6d Pde6g Pde6h Pde7b Pde8b Pde9a Pde10a Pde11a Pde12
B
IBMX (250 μM) PDE
cyto-bPAC
200 μm
1 mm
C
Control (DMSO)
IBMX (250 μM) PDE
Rolipram (10 μM) PDE4
WT
D
cyst number (x10²)
p = 0.01
p = 0.01
IBMX
Rolipram
cyst area (x10⁷ μm)
p = 0.02
p = 4x10⁻³
E
MR-L8 PDE4
0 μM
3 μM
10 μM
cilia-bPAC
F
cyst number (%)
cyst area (%)
Light
MR-L8
G
Forskolin 10 μM, MR-L8 0 μM
Forskolin 10 μM, MR-L8 3 μM
Forskolin 10 μM, MR-L8 10 μM
H
cyst number (%)
p < 10⁻⁴
p = 0.06
cyst area (%)
p < 10⁻⁴
p = 0.04
Forskolin
MR-L8

Figure EV2.

Figure EV3. Forskolin increases ciliary cAMP levels.

A Ciliary cAMP dynamics in wild-type (WT) mIMCD-3 cells measured using 5-HT6-mCherry-cADDis. After baseline measurements, cells were stimulated with 40 µM of Forskolin. The ratio of ciliary mCherry/cpEGFP fluorescence, normalized to the first 120 s of the recording, is shown as median (points, solid line) ± interquartile range (dotted lines) of 12 cilia (biological replicates) from $n = 3$ independent experiments.

B Ciliary cAMP increase after Forskolin stimulation. Each data-point represents one cilium; data is shown as median normalized ratio ± interquartile range of the time points indicated in light green in (A); *P*-value is indicated for Wilcoxon Singed Rank Test compared to 1.0.

C 3D culture of wild-type mIMCD-3 cells incubated with 100 nM PGE2 and DMSO (control) or 3 µM AH6809, an EP2-inhibitor. The PGE2 stimulus started 2 days later than AH6809 incubation. Exemplary images are shown ($n = 3$). Scale bars are indicated.

D 3D culture of *Ift20*$^{-/-}$ mIMCD-3 cells incubated with 100 nM PGE2 and acetone (control). Exemplary images are shown ($n = 3$). Scale bars are indicated.

E Quantification of images exemplified in (D). Data were normalized to matching wild-type controls (set to 100%) and are shown as mean ± SD. Data points show individual experiments.

F 3D culture of *Ift20*$^{-/-}$ mIMCD-3 cells incubated with 10 µM Forskolin and DMSO (control). Exemplary images are shown ($n = 3$). Scale bars are indicated.

G Quantification of images exemplified in (F). Data were normalized to matching wild-type controls (set to 100%) and are shown as mean ± SD. Data points show individual experiments.

H ELISA-based measurements of total cAMP levels from wild-type (WT) mIMCD-3 stimulated with DMSO or acetone (as control) and 10 µM Forskolin (1 h), or 100 nM PGE2 (1 h). Data are shown as mean ± SD, data points show individual experiments ($n = 3$–4), *P*-values calculated using a paired, two-sided Student's *t*-test are indicated.

I Cytoplasmic cAMP levels in wild-type mIMCD-3 cells were measured before and after stimulation with 100 nM PGE2 using the cytosolic, non-ratiometric cADDis cAMP biosensor. Data are shown as ratio 1/cADDis fluorescence and normalized to $t = 0$-60 s. Data are shown as median (points, solid line) ± interquartile range (dotted lines) of 28 cells (biological replicates) from $n = 4$ individual experiments.

J Individual values for region highlighted in green in (I). Data are shown as median ± interquartile range; *P*-value is indicated for Wilcoxon Singed Rank Test compared to 1.0. Data represents 28 cells (biological replicates) from $n = 4$ individual experiments.

© 2022 The Authors

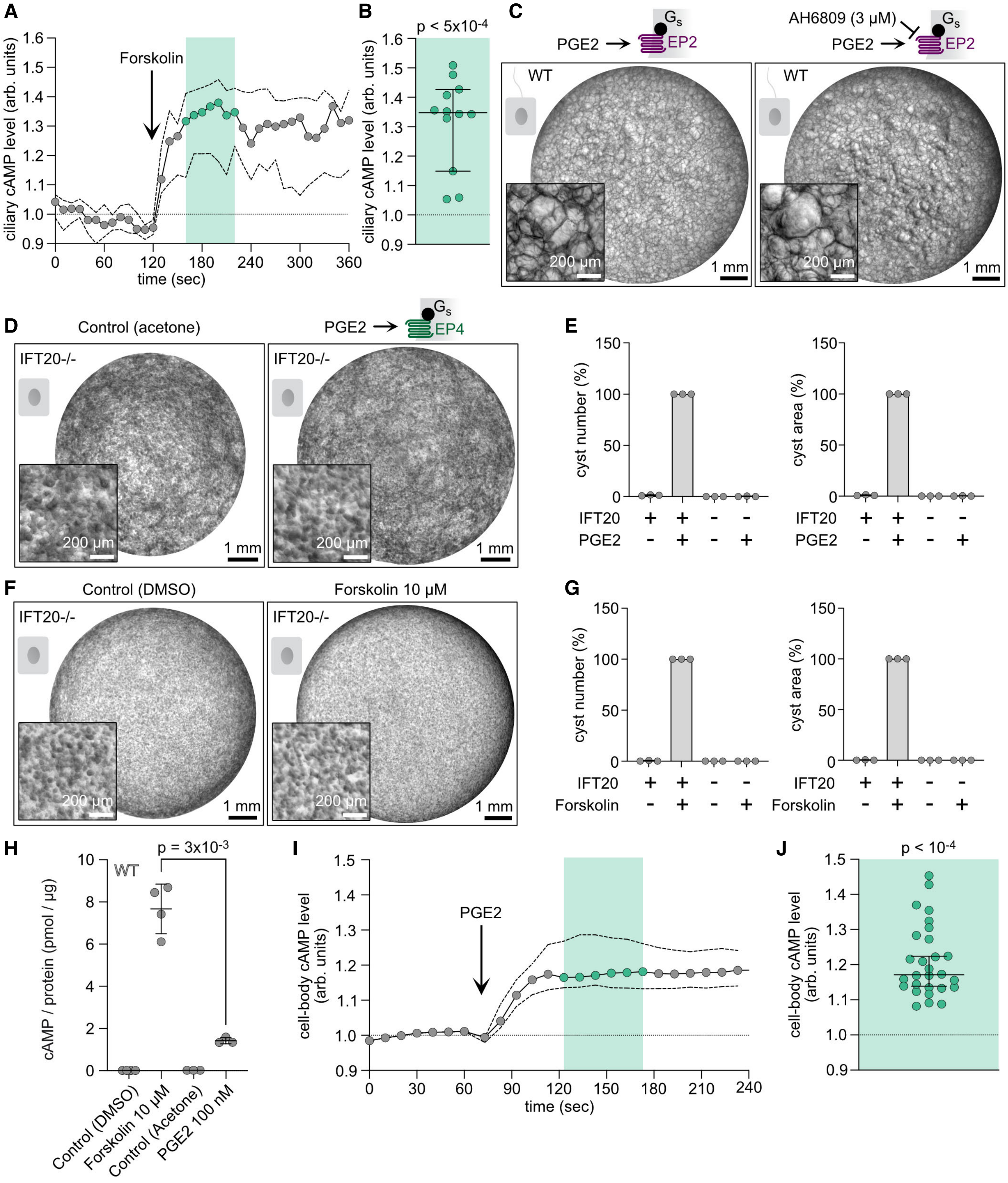
A
Forskolin
ciliary cAMP level (arb. units)
time (sec)
B
p < 5x10-4
ciliary cAMP level (arb. units)
C
PGE2 → Gs EP2
AH6809 (3 µM)
PGE2 → Gs EP2
WT
200 µm
1 mm
D
Control (acetone)
PGE2 → Gs EP4
IFT20-/-
200 µm
1 mm
E
cyst number (%)
cyst area (%)
IFT20 + + - -
PGE2 - + - +
F
Control (DMSO)
Forskolin 10 µM
IFT20-/-
200 µm
1 mm
G
cyst number (%)
cyst area (%)
IFT20 + + - -
Forskolin - + - +
H
p = 3x10-3
WT
cAMP / protein (pmol / µg)
Control (DMSO)
Forskolin 10 µM
Control (Acetone)
PGE2 100 nM
I
PGE2
cell-body cAMP level (arb. units)
time (sec)
J
p < 10-4
cell-body cAMP level (arb. units)

Figure EV3.

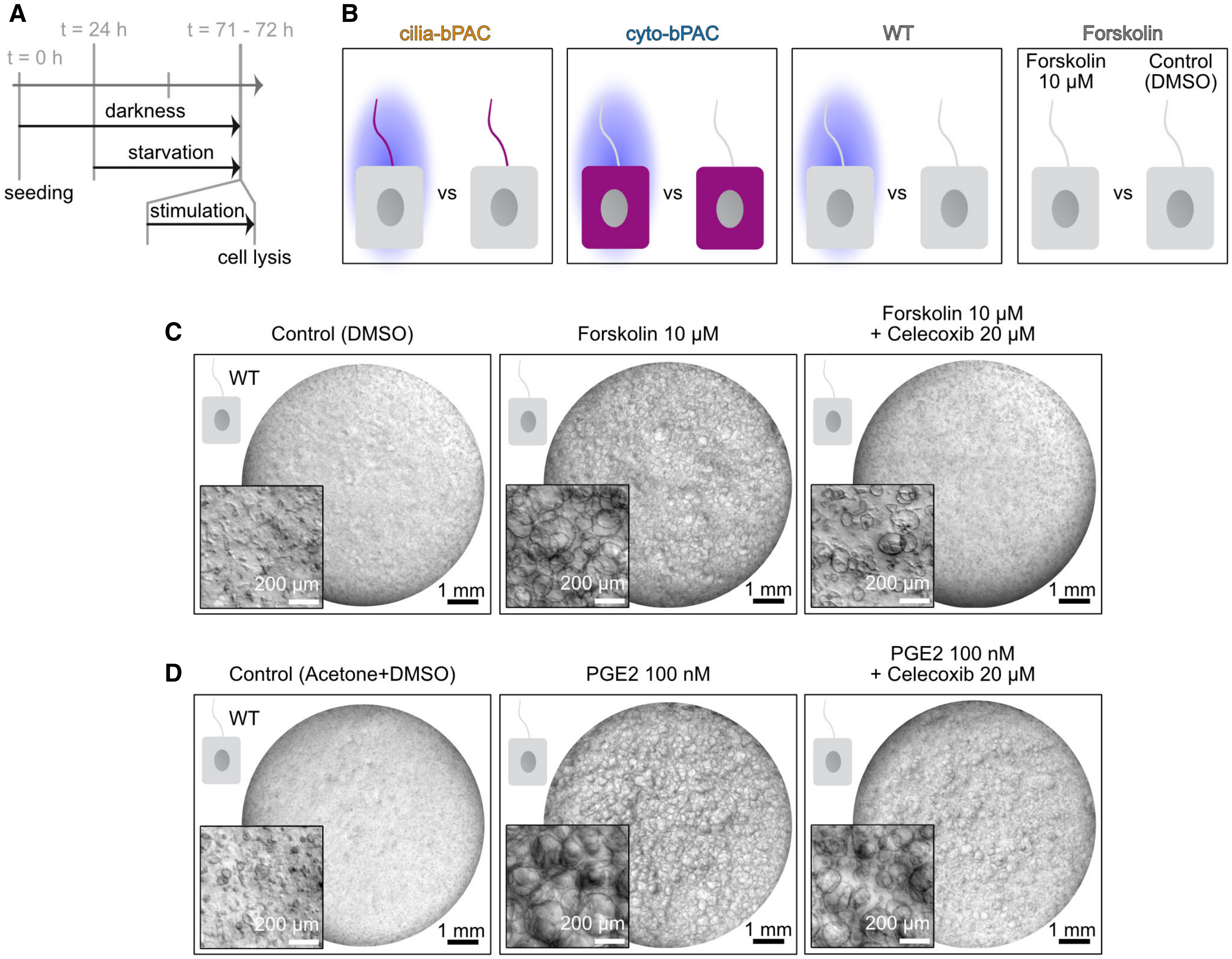

Figure EV4. RNA sequencing and signaling downstream of ciliary cAMP.

A Experimental approach. mIMCD-3 cells were in the dark, starved 24 h post seeding, and stimulated 71 h post seeding for 1 h with the conditions shown in (B), followed by immediate cell lysis.

B Conditions compared in the transcriptomics experiment: wild-type (WT) mIMCD-3 cells and mIMCD-3 cells stably expressing cilia-bPAC or cyto-bPAC were stimulated for 1 h by light (465 nm, 38.8 µW/cm²) or kept in the dark (as control). WT cells were stimulated with DMSO (control) or 10 µM of Forskolin (1 h).

C Wild-type (WT) mIMCD-3 cells cultured in a 3D matrix during continuous exposure to DMSO (control), 10 µM Forskolin, or Forskolin plus 20 µM celecoxib. Exemplary images are shown (n = 3).

D Wild-type (WT) mIMCD-3 cells cultured in a 3D matrix during continuous exposure to acetone (control), 10 nM PGE2, or PGE2 plus 20 µM celecoxib. Exemplary images are shown (n = 3).

 © 2022 The Authors
