# Supplementary material for: A cAMP signalosome in primary cilia drives gene expression and kidney cyst formation
Source: EMBO Rep. 2022 Jun 13;23(8):e54315. doi: 10.15252/embr.202154315 (PMC9346484; doi:10.15252/embr.202154315)
Supplement: Supplementary file 4 [file EMBR-23-e54315-s002.pdf]

EMBO reports

A cAMP signalosome in primary cilia drives gene expression and kidney cyst formation

Jan Hansen, Fabian Kaiser, Philipp Leyendecker, Birthe Stüven, Jens-Henning Krause, Fatemeh Derakhshandeh, Jaazba Irfan, Tommy Sroka, Kenley Preval, Paurav Desai, Michael Kraut, Heidi Theis, Anna-Dorothee Drews, Elena De-Domenico, Kristian Händler, Gregory J. Pazour, David Henderson, David Mick, and Dagmar Wachten
DOI: 10.15252/embr.202154315

Corresponding author(s): Dagmar Wachten (dwachten@uni-bonn.de)

Review Timeline:

Submission Date:	11th Nov 21
Editorial Decision:	15th Dec 21
Revision Received:	11th Apr 22
Editorial Decision:	9th May 22
Revision Received:	11th May 22
Accepted:	19th May 22

Editor: Deniz Senyilmaz Tiebe

Transaction Report:

(Note: With the exception of the correction of typographical or spelling errors that could be a source of ambiguity, letters and reports are not edited. Depending on transfer agreements, referee reports obtained elsewhere may or may not be included in this compilation. Referee reports are anonymous unless the Referee chooses to sign their reports.)

1st Editorial Decision 15th Dec 2021

Dear Dr. Wachten,

Thank you for the submission of your research manuscript to our journal, which was now seen by three referees, whose reports are copied below.

We concur with the referees that the proposed ciliary cAMP signalosome in principle very interesting. However, referees raise significant concerns that need to be addressed to consider publication here.

I find the reports informed and constructive, and believe that addressing the concerns raised will significantly strengthen the manuscript. As the reports are below, and I think all points need to be addressed, I will not detail them here.

Given these positive recommendations, we would like to invite you to revise your manuscript with the understanding that the referee concerns (as in their reports) must be fully addressed and their suggestions taken on board. Please address all referee concerns in a complete point-by-point response. Acceptance of the manuscript will depend on a positive outcome of a second round of review. It is EMBO reports policy to allow a single round of revision only and acceptance or rejection of the manuscript will therefore depend on the completeness of your responses included in the next, final version of the manuscript.

We generally allow three months as standard revision time. As a matter of policy, competing manuscripts published during this period will not negatively impact on our assessment of the conceptual advance presented by your study. However, we request that you contact the editor as soon as possible upon publication of any related work, to discuss how to proceed. Should you foresee a problem in meeting this three-month deadline, please let us know in advance and we may be able to grant an extension.

*** Temporary update to EMBO Press scooping protection policy:
We are aware that many laboratories cannot function at full efficiency during the current COVID-19/SARS-CoV-2 pandemic and have therefore extended our 'scooping protection policy' to cover the period required for a full revision to address the experimental issues highlighted in the editorial decision letter. Please contact the scientific editor handling your manuscript to discuss a revision plan should you need additional time, and also if you see a paper with related content published elsewhere.***

IMPORTANT NOTE: we perform an initial quality control of all revised manuscripts before re-review. Your manuscript will FAIL this control and the handling will be DELAYED if the following APPLIES:
1. A data availability section providing access to data deposited in public databases is missing (where applicable).
2. Your manuscript contains statistics and error bars based on n=2. Please use scatter plots in these cases.

You can submit the revision either as a Scientific Report or as a Research Article. For Scientific Reports, the revised manuscript can contain up to 5 main figures and 5 Expanded View figures. If the revision leads to a manuscript with more than 5 main figures it will be published as a Research Article. In this case the Results and Discussion section should be separate. If a Scientific Report is submitted, these sections have to be combined. This will help to shorten the manuscript text by eliminating some redundancy that is inevitable when discussing the same experiments twice. In either case, all materials and methods should be included in the main manuscript file

Supplementary/additional data: The Expanded View format, which will be displayed in the main HTML of the paper in a collapsible format, has replaced the Supplementary information. You can submit up to 5 images as Expanded View. Please follow the nomenclature Figure EV1, Figure EV2 etc. The figure legend for these should be included in the main manuscript document file in a section called Expanded View Figure Legends after the main Figure Legends section. Additional Supplementary material should be supplied as a single pdf labeled Appendix. The Appendix includes a table of content on the first page with page numbers, all figures and their legends. Please follow the nomenclature Appendix Figure Sx throughout the text and also label the figures according to this nomenclature. For more details please refer to our guide to authors.

Please note that for all articles published beginning 1 July 2020, the EMBO Reports reference style will change to the Harvard style for all article types. Details and examples are provided at
https://www.embopress.org/page/journal/14693178/authorguide#referencesformat

When submitting your revised manuscript, please carefully review the instructions that follow below. Failure to include requested items will delay the evaluation of your revision.

1) a .docx formatted version of the manuscript text (including legends for main figures, EV figures and tables). Please make sure that the changes are highlighted to be clearly visible.

2) individual production quality figure files as .eps, .tif, .jpg (one file per figure).

3) a .docx formatted letter INCLUDING the reviewers' reports and your detailed point-by-point responses to their comments. As

part of the EMBO Press transparent editorial process, the point-by-point response is part of the Review Process File (RPF), which will be published alongside your paper. For more details on our Transparent Editorial Process, please visit our website: https://www.embopress.org/page/journal/14693178/authorguide#transparentprocess
You are able to opt out of this by letting the editorial office know (emboreports@embo.org). If you do opt out, the Review Process File link will point to the following statement: "No Review Process File is available with this article, as the authors have chosen not to make the review process public in this case."

4) a complete author checklist, which you can download from our author guidelines (). Please insert information in the checklist that is also reflected in the manuscript. The completed author checklist will also be part of the RPF.

5) Please note that all corresponding authors are required to supply an ORCID ID for their name upon submission of a revised manuscript (). Please find instructions on how to link your ORCID ID to your account in our manuscript tracking system in our Author guidelines ().

6) We replaced Supplementary Information with Expanded View (EV) Figures and Tables that are collapsible/expandable online. A maximum of 5 EV Figures can be typeset. EV Figures should be cited as 'Figure EV1, Figure EV2" etc... in the text and their respective legends should be included in the main text after the legends of regular figures.

- For the figures that you do NOT wish to display as Expanded View figures, they should be bundled together with their legends in a single PDF file called *Appendix*, which should start with a short Table of Content. Appendix figures should be referred to in the main text as: "Appendix Figure S1, Appendix Figure S2" etc. See detailed instructions regarding expanded view here: .

- Additional Tables/Datasets should be labeled and referred to as Table EV1, Dataset EV1, etc. Legends have to be provided in a separate tab in case of .xls files. Alternatively, the legend can be supplied as a separate text file (README) and zipped together with the Table/Dataset file.

7) We would also encourage you to include the source data for figure panels that show essential data.

Numerical data should be provided as individual .xls or .csv files (including a tab describing the data). For blots or microscopy, uncropped images should be submitted (using a zip archive if multiple images need to be supplied for one panel). Additional information on source data and instruction on how to label the files are available .

8) Our journal encourages inclusion of *data citations in the reference list* to directly cite datasets that were re-used and obtained from public databases. Data citations in the article text are distinct from normal bibliographical citations and should directly link to the database records from which the data can be accessed. In the main text, data citations are formatted as follows: "Data ref: Smith et al, 2001" or "Data ref: NCBI Sequence Read Archive PRJNA342805, 2017". In the Reference list, data citations must be labeled with "[DATASET]". A data reference must provide the database name, accession number/identifiers and a resolvable link to the landing page from which the data can be accessed at the end of the reference. Further instructions are available at .

9) Please make sure to include a Data Availability Section before submitting your revision - if it is not applicable, make a statement that no data were deposited in a public database. Primary datasets (and computer code, where appropriate) produced in this study need to be deposited in an appropriate public database (see).

Please remember to provide a reviewer password if the datasets are not yet public.

The accession numbers and database should be listed in a formal "Data Availability " section (placed after Materials & Method) that follows the model below. Please note that the Data Availability Section is restricted to new primary data that are part of this study.

Data availability

The datasets (and computer code) produced in this study are available in the following databases:

- RNA-Seq data: Gene Expression Omnibus GSE46843 (https://www.ncbi.nlm.nih.gov/geo/query/acc.cgi?acc=GSE46843)
- [data type]: [name of the resource] [accession number/identifier/doi] ([URL or identifiers.org/DATABASE:ACCESSION])

*** Note - All links should resolve to a page where the data can be accessed. ***

10) Regarding data quantification, please ensure to specify the name of the statistical test used to generate error bars and P values, the number (n) of independent experiments underlying each data point (not replicate measures of one sample), and the

test used to calculate p-values in each figure legend. Discussion of statistical methodology can be reported in the materials and methods section, but figure legends should contain a basic description of n, P and the test applied.
Please note that error bars and statistical comparisons may only be applied to data obtained from at least three independent biological replicates.
Please also include scale bars in all microscopy images.

We would also welcome the submission of cover suggestions, or motifs to be used by our Graphics Illustrator in designing a cover.

I look forward to seeing a revised version of your manuscript when it is ready. Please let me know if you have questions or comments regarding the revision.

Kind regards,

Deniz Senyilmaz Tiebe

Deniz Senyilmaz Tiebe, PhD
Editor
EMBO Reports

Referee #1:

The manuscript by Hansen et al postulates the function of a "novel cAMP signalosome" in primary cilia in kidney cyst formation. Although cellular cAMP and nuclear pCREB levels are elevated in renal cysts from multiple pathologies and reduction of cAMP production have been shown to reduce polycystic kidney disease, the role of cilia in these processes are largely unknown. The authors use an optogenetic strategy to elevate cAMP at distinct subcellular locations and show that such perturbations over days cause cysts in 3D cultures of renal epithelial cells. Counteracting this chronic cAMP elevation in the cilium by phosphodiesterase inhibitors prevent cyst growth. Using RNA sequencing and cilia imaging, they propose the functioning of a novel cAMP signalosome that is functionally distinct from the cytoplasm.

The basic premise of the paper in demonstrating the role of ciliary cAMP levels in driving renal cystogenesis at least in 3D cultures is extremely novel, will be of broad interest to readers from multiple fields, and merits immediate publication.

However, I am a bit guarded on my enthusiasm for the proposed ciliary signalosome complex, which probably needs further validation and/or clarification before publication. Especially, the authors propose that cAMP levels in cilia are critical in cAMP induced signalling through ciliary localized PGE2 and pCREB signalling. At this point it is not clear if ciliary localization of PGE2 receptor, concurrent ciliary cAMP signaling from PGE2 administration, and pCREB formation in cilia are mechanisms that drive cyst formation or rather are just associated with this process. Of course, both cilia bPAC and Forskolin treated cells have increased Ptgs2 expression, but does PGE2 function in cyst formation through cilia? Especially, does Forskolin treatment also cause cyst formation by increased cAMP levels in cilia? Addressing or discussing some of these questions might provide a clearer model of the proposed cAMP signalosome in cilia in driving, rather than just accompanying, cyst pathogenesis.

Other comments related to individual figures:

1. Fig. 1. cAMP levels are shown to leak into cilia from the cytoplasm in cyto bPAC cells at levels comparable (only 20% or less difference between means and just barely significant) to that from direct stimulation of bPAC in cilia. Does further increase in cytoplasmic cAMP levels (say by light stimulus changes or increasing levels of bPAC in cells) cause cysts and are associated with more ciliary cAMP levels? It's difficult to believe that a subtle change of 20% or so of ciliary cAMP levels would drive cyst formation. Similarly, does cytoplasmic cAMP levels increase from ciliary bPAC stimulation?

2. Fig 2. Please define "cyst" in methods (e. g. size, shape, 3D volume etc). Normally cells form tubules and some cysts in similar 3D assays. From the provided insets it is very difficult to appreciate how the authors are quantifying cysts vs tubules. Cyst formation in cyto-bPAC cells seem to present in resting cells and light stimulation paradoxically reduces cyst formation. Please explain why Forskolin treatment in wild type cells in panels 2A vs 2E show large variability in both cyst number and size.

3. Fig. 3. Does pS6RP level increase precede cyst formation? Does rapamycin treatment reduce cyst formation?

4. Fig. 5. The authors use cADDIs instead of Pink Flamido in this figure. I am curious why so. Anyways, although ciliary cAMP levels increase upon PGE2 treatment, what happens to cytoplasmic levels, and/or total cellular cAMP levels. Does such increase only happen in cilia? Are these ciliary cAMP levels commensurate with the levels seen required for cyst formation (although measured using a different cAMP sensor in this case from Fig 1)?

5. Fig. 6. The authors show that both cilia bPAC and Forskolin treatment causes increased Ptgs2 expression. Celecoxib treatment prevents light-induced cyst formation in cilia-bPAC cells. Does similar treatment prevent Forskolin-induced cyst formation? Can the authors comment if PGE2 induced cAMP production function in driving or enabling cAMP-driven cyst formation?

6. Fig. 7. pCREB localization in cilia is very interesting but does such pCREB formation occur in cyto bPAC expressing +/- ciliobrevin treated cells as well? It would be good to see if nuclear pCREB is present in the cAMP-induced cysts, and whether there is any temporal correlation between such ciliary localization with respect to nuclear translocation. Does such pCREB formation in cilia occur in Forskolin +/- ciliobrevin treated cells? The connection between PKA and mTOR in the cartoon is not clear at least from the performed experiments.

Referee #2:

This manuscript addresses important, unaswered questions about the unique properties of the ciliary compartment that underlie the role of the organelle in multiple signaling pathways whose components are also present in the cytoplasm. The authors targeted a light-regulated adenylyl cyclase either the cilium or the cytoplasm. In compelling experiments, they showed that increases in ciliary cAMP promoted cystogenesis in their in vitro model system of kidney tubules, whereas increases in cytoplasmic cAMP did not. They went on to investigate the uniquely ciliary events required and showed that PKA-dependent phosphorylation of a key transcription factor involved in cystogenesis required IFT-dependent ciliary trafficking. Many current models suggest, with little evidence, that cAMP diffusing from cilia is critical for downstream responses to ciliary receptor engagement, and these findings provide evidence against this model. This work provides exciting new insights into mechanisms of cilium to cytoplasm signaling and demonstrates that the organelle has the capacity to support multiple features of a signaling pathway that are independent of cytoplasmic events. (What happens in cilia, stays in cilia.) Given that polycystic kidney disease is among the most common monogenic inherited diseases in human, these finding will also be relevant to the clinical community. Below I outline a few minor concerns.

Figure 1: The differences in cAMP between cilia vs cyto bPAC are very small, even though difference is statistically significant. Also, it was difficult to interpret the number of experiments and the number of cilia assessed? If understood correctly, the cilia-bPAC experiment was done 8 times, and from these 8 experiments the fluorescence was quantified from a total of 14 cilia? Given the centrality of this information to the thrust of this work, need to provide results from many more cilia in each group.

Figure 2: Authors need to comment on comparatively low cyst formation in the cyto-bPAC samples in Fig. 2E?

MR-L8 experiments: Need reference. Also, did agent decrease ciliary cAMP in the cilia-bPAC, light-treated cells?

Figure 5: In Jin, Ni 2014 paper, EP4 was present in cilia but also at the cell membrane. This was most obvious in the RPE1 cells (Jin fig7I, but also in the bottom-most IMCD3 cell in Fig7L). Need evidence that cytoplasmic cAMP did not increase in these experiments. Unless the authors document that EP4 is uniquely localized to cilia in these experiments, this conclusion is not warranted by the results.

Figure 6: Need to comment on possibility that Celecoxib speciically effects the cystogenic pathway as opposed to non-specific inhibition of cell proliferation.

Referee #3:

The study by Hansen et al. investigates the role of ciliary cAMP in the regulation of cyst growth. The authors use the established murine inner medullary collecting duct cell line IMCD3, which can be used to study 3D cyst formation. By using an elegant optogenetic approach, they are able to induce cAMP formation inside the primary cilium by light. Interestingly, this affects cyst growth and proliferation, while cytoplasmic expression of the same p.a. enzyme does not. By RNAseq, the authors analyze transcriptional changes upon the ciliary cAMP increase and identify the CREB TF. Phospho CREB occurs in the cilium upon cAMP induction.

This is a fascinating manuscript and an important study on a very relevant topic of cilia biology. It is very well written. I really like the story, but I have a number of concerns that should be addressed by the authors.

Major points:

1) The authors surprisingly show that ciliary bPAC but not cyto-bPAC results in cyst formation/cyst growth. This is exciting and, for me, the key finding of this study! However, I am not entirely convinced that cytobPAC, with its diffuse and non-targeted expression pattern, is the appropriate control here. Given the importance of this result for the entire study, I would suggest using additional controls with bPAC.mCherry targeted to the plasma membrane, the ER, or the outer mitochondrial membrane. Perhaps high local concentrations at one specific spot are necessary to exert an effect.
2) The authors suggest that the Forskolin effect in Fig. 1 primarily results from ciliary cAMP increase. It would be good to demonstrate that Forskolin indeed enhances ciliary cAMP levels (e.g., by a cilia-Flamindo assay).
3) Where does cilia-bPAC localize in non-ciliated, mitotic cells? Is it active? This could be relevant since the cystformation-assay takes several days.
4) With respect to the PDEs and EB4, it is unclear if these proteins are localized exclusively to primary cilia. They might occur at the apical or basolateral membrane and exert cilia-independent effects.
5) While it is striking to find CREB in the cilium and to speculate that CREB might shuttle back and forth from the cilium to the nucleus, this is actually not demonstrated by this study.

Minor issues:

1) The exact timing of the experiments remains unclear. E.g., in Fig 2 C/D: How long were cell treated with Forskolin? Is the 1h on/off cycling required for the observed effects? What is the reason for the cycling?
2) Why did the authors switch to wt control cells in Fig 3? It might be important to know whether cytoplasmic cAMP would also affect proliferation and mTOR (even without promoting cyst formation/growth).
3) While it is not surprising that rapamycin reduces pS6RP, it would be essential to see whether rapamycin affects proliferation induced by cilia bPAC (as shown by the KI67 increase in Fig 3 A_C). If the authors aim to conclude that "ciliary cAMP engages mTOR signaling to promote cell proliferation", this should be demonstrated by rapamycin treatment of the cultures presented in Fig. 2.
4) Full blots should be provided for Fig 4D.
5) The concentration of the COX2 inhibitor with 20 µM is quite high. The EC50 is about 40nM (?). It might not be a COX2 specific effect. Can the authors reduce the concentration?
6) I would suggest being more cautious when linking this story to ADPKD, in particular at the end of the abstract or in the middle of the introduction. Btw: the abstract and introduction share the very same last sentence.
The result section starts with "To investigate the role of ciliary cAMP signaling in kidney cyst growth, we used mIMCD-3 ....". It should become clearer throughout the MS, that's this IMCD assay is a somehow accepted 3D cell model but also quite limited in terms of "kidney cyst growth." The latter can only be studied in vivo.

Referee #1:

The manuscript by Hansen et al postulates the function of a "novel cAMP signalosome" in primary cilia in kidney cyst formation. Although cellular cAMP and nuclear pCREB levels are elevated in renal cysts from multiple pathologies and reduction of cAMP production have been shown to reduce polycystic kidney disease, the role of cilia in these processes are largely unknown. The authors use an optogenetic strategy to elevate cAMP at distinct subcellular locations and show that such perturbations over days cause cysts in 3D cultures of renal epithelial cells. Counteracting this chronic cAMP elevation in the cilium by phosphodiesterase inhibitors prevent cyst growth. Using RNA sequencing and cilia imaging, they propose the functioning of a novel cAMP signalosome that is functionally distinct from the cytoplasm.
The basic premise of the paper in demonstrating the role of ciliary cAMP levels in driving renal cystogenesis at least in 3D cultures is extremely novel, will be of broad interest to readers from multiple fields, and merits immediate publication.
However, I am a bit guarded on my enthusiasm for the proposed ciliary signalosome complex, which probably needs further validation and/or clarification before publication.

Especially, the authors propose that cAMP levels in cilia are critical in cAMP induced signalling through ciliary localized PGE2 and pCREB signalling. At this point it is not clear if ciliary localization of PGE2 receptor, concurrent ciliary cAMP signaling from PGE2 administration, and pCREB formation in cilia are mechanisms that drive cyst formation or rather are just associated with this process. Of course, both cilia bPAC and Forskolin treated cells have increased Ptgs2 expression, but does PGE2 function in cyst formation through cilia?

We would like to thank the reviewer for pointing out the importance of our manuscript and its interest to a broad readership. We also appreciate the important questions that the reviewer brought up and we are happy to address and discuss them in the following.
It has been shown before that PGE2 induces cyst formation in epithelial cells (e.g., Elberg et al., Am J. Physiol. Renal Physiol. 2007, Omar et al., PNAS 2019). The action of PGE2 relies on activation of EP receptors. Here, we show that PGE2 action in IMCD-3 cells relies on EP4 activation (Fig. 5G-H), which has also been demonstrated by others (Elberg et al., Prost. Lipid Med. 2012). PGE2 also evokes an increase in ciliary cAMP levels (Fig. 5B-C), indicating that it evokes a response via the primary cilium. In fact, the EP4 receptor has been shown to localize to primary cilia in hRPE1 and mIMCD-3 cells (Jin et al., Nat. Cell Biol. 2014), and we now also provide experimental evidence that also in our mIMCD-3 cells, the EP4 receptor is enriched in primary cilia (Fig. 5A). Furthermore, MR-L8, which activates PDE4 long isoforms and blocks cyst formation induced by ciliary cAMP signaling (Fig. 4H, I), blocks PGE2-induced cyst formation (Fig. 5F). In addition, we also tested whether PGE2-evoked cyst development is cilia-dependent: in fact, PGE2 did not induce cyst formation in *Ift20*$^{-/-}$ mIMCD-3 cells (Fig. EV3D, E). PGE2-dependent cystogenesis, as ciliary cAMP-induced cystogenesis, relies on downstream COX-2 action, as we now demonstrate that celecoxib blocks PGE2-dependent cyst formation (Fig. 6I). Thus, there is compelling experimental evidence collected by us and others, demonstrating that PGE2 induces cyst formation via primary cilia signaling. However, as it is still difficult to demonstrate that PGE2 exclusively functions via the primary cilium, we have changed the text in the manuscript to account for this.
Whether PGE2/chronic ciliary cAMP signaling is also sufficient to drive cyst development i*n vivo*, has to be revealed in future studies.

Especially, does Forskolin treatment also cause cyst formation by increased cAMP levels in cilia?

We now provide data, demonstrating that indeed Forskolin increases ciliary cAMP levels (Fig. EV3A-B). We also analyzed whether the Forskolin-dependent cyst formation is

controlled by PDE4 long isoform activity, which we have shown to control the ciliary cAMP-dependent cyst formation. Indeed, MR-L8, the PDE4 long isoform activator, blocks Forskolin-dependent cyst formation (Fig. EV2D, E).

Addressing or discussing some of these questions might provide a clearer model of the proposed cAMP signalosome in cilia in driving, rather than just accompanying, cyst pathogenesis.

We have incorporated all the new data into the revised version of the manuscript.

Other comments related to individual figures:

1. *Fig. 1. cAMP levels are shown to leak into cilia from the cytoplasm in cyto bPAC cells at levels comparable (only 20% or less difference between means and just barely significant) to that from direct stimulation of bPAC in cilia. Does further increase in cytoplasmic cAMP levels (say by light stimulus changes or increasing levels of bPAC in cells) cause cysts and are associated with more ciliary cAMP levels? It's difficult to believe that a subtle change of 20% or so of ciliary cAMP levels would drive cyst formation. Similarly, does cytoplasmic cAMP levels increase from ciliary bPAC stimulation?*

We thank the reviewer for raising this important point. The whole system relies on a tight balance between cAMP levels in the cilium and cytoplasm. We have carefully evaluated the changes in cAMP levels that we evoke using optogenetics in both compartments and intentionally used cell lines expressing low levels of the respective optogenetic tools that provide the necessary specificity, i.e., increasing cAMP levels in the respective compartment without dramatically altering cAMP levels in the other compartment. To demonstrate the specificity of this approach, we have performed further experiments, demonstrating that an increase in ciliary cAMP levels using photoactivation of cilia-bPAC does not significantly change cAMP levels in the cytoplasm, as determined by biosensor expression in the cytoplasm (see Figure 1 below). This data is supported by the measurement of total cAMP levels, which are almost exclusively determined by cytoplasmic cAMP levels and did not show an increase in cAMP levels upon cilia-bPAC activation (Fig. 1C). Our results further indicated that the activity of PDE4 long isoforms seems to be important to maintain the ciliary cAMP compartment, blocking entry of cytosolic cAMP into the cilium. To verify this hypothesis, we blocked PDE4 activity using rolipram during photoactivation of cyto-bPAC, and analyzed ciliary cAMP levels and cyst formation. In the presence of rolipram, activation of cyto-bPAC induced some cyst formation (Fig. 4C, D) and also increased ciliary cAMP levels (Fig. 4E), demonstrating that PDE4 activity is crucial to functionally separate the ciliary cAMP compartment from the cytoplasm. If PDE4 activity is inhibited, cytosolic cAMP can accumulate in the cilium and evokes a ciliary cAMP response.

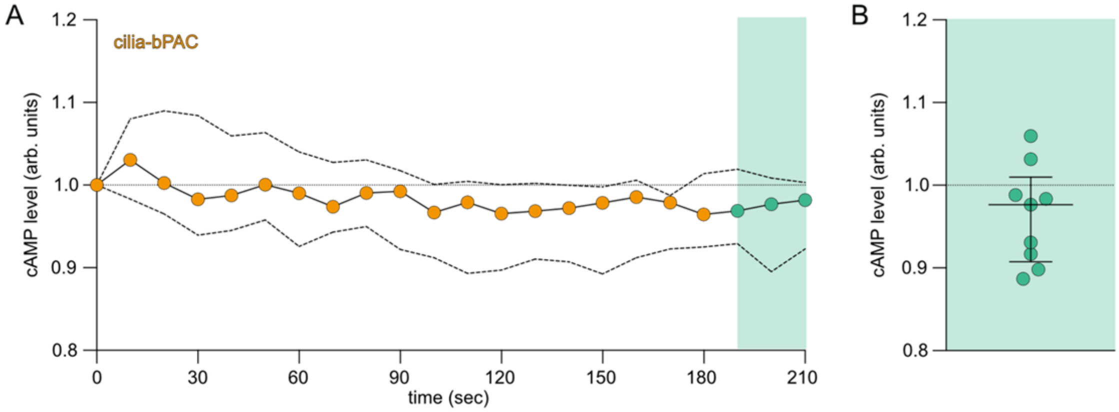

Figure 1: Cytoplasmic cAMP levels. Cytoplasmic cAMP levels in cilia-bPAC mIMCD-3 cells were measured using the cytosolic, non-ratiometric cADDis cAMP biosensor on a spinning disk microscope. **(A)** Time course. Data is shown as ratio 1/cADDis fluorescence and normalized to t = 0. Data are shown as mean (points, solid line) ± interquartile range (dotted lines) of n = 4 (9 cells). **(B)** Individual values for region highlighted in green in (A). Data are shown as median ± interquartile range.

2. *Fig 2. Please define "cyst" in methods (e. g. size, shape, 3D volume etc). Normally cells form tubules and somea cysts in similar 3D assays. From the provided insets it is very difficult to appreciate how the authors are quantifying cysts vs tubules. Cyst formation in cyto-bPAC cells seem to present in resting cells and light stimulation paradoxically reduces cyst formation. Please explain why Forskolin treatment in wild type cells in panels 2A vs 2E show large variability in both cyst number and size.*

This is an important point. We have added a detailed description and graphical presentation how we analyze the cysts in our images in the revised version of the manuscript (Appendix Fig. S1). Only cysts with a clearly visible lumen were considered in quantifications and tiny cysts, which were difficult to distinguish from cellular agglomerates were not included in the quantifications.

In general, cAMP in the cilium and the cytoplasm seem to have opposing effects. We have already demonstrated this for the ciliary length control (Hansen et al., eLife 2020). Here, we show that this concept also applies to cyst development when activating bPAC and increasing cAMP levels in the different compartments by light. However, bPAC also displays a dark activity, which might influence cellular signaling to some extent, even in the dark. This might also explain the mild cyst formation seen in cyto-bPAC cells in the dark. In the dark, basal cAMP levels in cyto-bPAC cells are slightly increased compared to control cells (Fig. 1C). This might also influence ciliary cAMP signaling, leading to cyst formation. To test this hypothesis, we analyzed cyst formation in cyto-bPAC cells in the presence of MR-L8 in the dark to activate PDE4 long isoforms and suppress any residual bPAC activity. Indeed, cyst formation in the dark was reduced when incubating the cells with MR-L8 (see Figure 2 below).

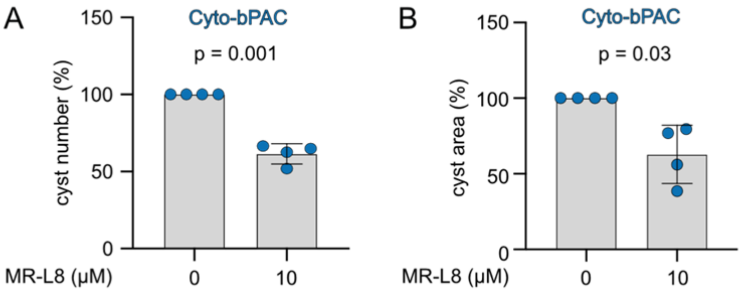

Figure 2: Cyst formation in cyto-bPAC mIMCD-3 cells in the dark. mIMCD-3 cells stably expressing cyto-bPAC were cultured in a 3D matrix in the dark and incubated with 10 µM MR-L8. **(A)** Quantification of the cyst number. **(B)** Quantification of the cyst area. Data are shown as mean ± S.D., each datapoint corresponds to an independent experiment; p values calculated using a paired, two-sided Student's t-test are indicated.

We also had a closer look at the forskolin data to understand where the variability is coming from. We noticed that for the experiments shown in the manuscript, different batches of the gel matrix for the 3D culture were used (different batch but same product number). This could explain the variability that we see in the forskolin data. Nevertheless, forskolin always robustly induced cyst formation.

3. *Fig. 3. Does pS6RP level increase precede cyst formation? Does rapamycin treatment reduce cyst formation?*

This is a good question and we performed the required experiments to address this question. Indeed, treatment with rapamycin, which reduced pS6RP levels in cilia-bPAC cells upon light stimulation (Fig. 3D-E), also fully abolished ciliary cAMP-dependent cyst formation (Fig. 3G-H).

4. *Fig. 5. The authors use cADDIs instead of Pink Flamido in this figure. I am curious why so. Anyways, although ciliary cAMP levels increase upon PGE2 treatment, what happens to cytoplasmic levels, and/or total cellular cAMP levels. Does such increase only happen in cilia? Are these ciliary cAMP levels commensurate with the levels seen required for cyst formation (although measured using a different cAMP sensor in this case from Fig 1)?*

We have used different genetically encoded biosensors depending on the assay. When using bPAC together with a biosensor in the same compartment, we have used Pink Flamindo, as it allows to image in a spectral range that does not activate bPAC. However, Pink Flamindo is very dim, which is why we prefer to use the cADDis sensor for the other applications, as it is brighter and shows a better signal-to-noise ratio.
We have analyzed cytoplasmic as well as total cAMP levels in response to PGE2 stimulation. PGE2 increased total cAMP levels, however, the response was reduced compared to Forskolin (Fig. EV3F). To determine the changes in cytoplasmic cAMP levels, we used mIMCD-3 cells expressing the cADDis cAMP sensor only in the cytoplasm and stimulated them with 100 nM PGE2. Indeed, PGE2 also evoked an increase in the cytoplasmic cAMP concentration (Fig. EV3G-H), demonstrating that PGE2 targets both, the ciliary and the cytoplasmic cAMP compartment. However, based on the evidence presented in response to the first question of the reviewer, we present experimental evidence that PGE2-dependent cyst formation predominantly relies on ciliary cAMP signaling.
To compare whether the ciliary cAMP levels generated by PGE2 stimulation are similar to the cAMP levels evoked after photoactivation of cilia-bPAC, we compared the relative changes in the two conditions. Stimulation with PGE2 evoked a median change of 6.2 % in the relative fluorescence whereas cilia-bPAC evoked a medium change of 7.7 %. Thus, the different stimuli seem to evoke similar responses.

5. *Fig. 6. The authors show that both cilia bPAC and Forskolin treatment causes increased Ptgs2 expression. Celecoxib treatment prevents light-induced cyst formation in cilia-bPAC cells. Does similar treatment prevent Forskolin-induced cyst formation? Can the authors comment if PGE2 induced cAMP production function in driving or enabling cAMP-driven cyst formation?*

In the original manuscript, we have shown that photoactivation cilia-bPAC and addition of Forskolin increases *Ptgs2* expression in mIMCD-3 cells (Fig. 6E). We have now performed additional experiments demonstrating that the ciliary cAMP-dependent increase in *Ptgs2* expression relies on ciliary export of proteins, as the upregulation of *Ptgs2* expression after photoactivation of cilia-bPAC was largely dampened in *Ift27*$^{-/-}$ compared to control mIMCD-3 cells (Fig. 7E). We have further performed experiments to analyze Forskolin-dependent cyst formation in the presence of celecoxib to block COX-2 activity. Indeed, celecoxib abolished

the Forskolin-dependent cyst formation (Fig. 6H) as it also blocks the PGE2- and ciliary cAMP-dependent response (Fig. 6G, I).
Regarding the question whether cAMP production is sufficient or only necessary but not sufficient for cyst formation: It has been shown by others and us that activating PDE4 long isoforms (MR-L2 Omar et al., PNAS 2019, MR-L8 our data) blocks PGE2-dependent cyst formation *in vitro*. As PGE2 increases ciliary cAMP levels and MR-L8 blocks ciliary cAMP-induced cyst formation, our conclusion would be that PGE2-induced cAMP production indeed is sufficient to induce cyst formation *in vitro*. However, we cannot rule out that *in vivo*, other signaling pathways contribute to PGE2-induced cyst formation.

6. *Fig. 7. pCREB localization in cilia is very interesting but does such pCREB formation occur in cyto bPAC expressing +/- ciliobrevin treated cells as well? It would be good to see if nuclear pCREB is present in the cAMP-induced cysts, and whether there is any temporal correlation between such ciliary localization with respect to nuclear translocation. Does such pCREB formation in cilia occur in Forskolin +/- ciliobrevin treated cells? The connection between PKA and mTOR in the cartoon is not clear at least from the performed experiments.*

We followed the reviewer's suggestion and investigated the localization of pCREB in cyto-bPAC after light stimulation in the presence of Ciliobrevin-D to block retrograde IFT. We could not observe pCREB localization in the cilium (Fig. 7A-B).
Furthermore, we also analyzed pCREB localization in 3D culture after 9 days of light-induced cyst formation in cilia-bPAC cells and could indeed reveal pCREB localization in the nucleus (see Figure 3 below). To analyze the temporal correlation between pCREB localization and the increase in ciliary cAMP levels, we also analyzed an earlier time point (15 min) after light stimulation in control (wild-type mIMCD-3) and in cilia-bPAC cells. Indeed, nuclear pCREB localization was already induced by an increase of ciliary cAMP levels at this earlier time point (see Figure 3 below).

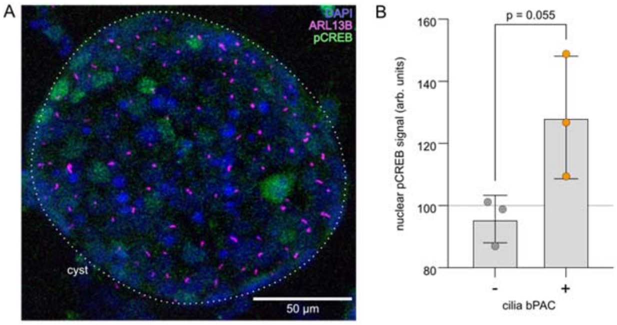

Figure 3: pCREB localization in the nucleus. (A) 3D culture of mIMCD-3 cells stably expressing cilia-bPAC were exposed to light (1 h dark, 465 nm, 38.8 µW/cm²) for 9 days. Cells were fixed and labeled with DAPI (blue) to label the DNA, an ARL13B antibody (pink) to label cilia, and a phospho-specific (Ser133) CREB antibody (pCREB, green). Scale bar is indicated. Dotted line indicates the cyst. **(B)** Quantification of nuclear pCREB localization in 2D cell culture. Wild-type or cilia-bPAC mIMCD-3 cells were exposed to light (15 min, 465 nm, 38.8 µW/cm²), fixed, and pCREB localization was analyzed using an anti-pCREB antibody together with DAPI as a counterstain. Data have been normalized to wild-type cells in the dark and are shown as mean ± S.D., each datapoint corresponds to an independent experiment; p values calculated using a paired, two-sided Student's t-test are indicated.

We also tried to analyze the Forskolin-dependent localization of pCREB but observed high data variability, possibly due to the dual action of Forskolin in both, the cilium and cytoplasm. Thus, we did not pursue this further.
We have also modified the cartoon according to the reviewer's suggestion.

Referee #2:

This manuscript addresses important, unanswered questions about the unique properties of the ciliary compartment that underlie the role of the organelle in multiple signaling pathways whose components are also present in the cytoplasm. The authors targeted a light-regulated adenylyl cyclase either the cilium or the cytoplasm. In compelling experiments, they showed that increases in ciliary cAMP promoted cystogenesis in their in vitro model system of kidney tubules, whereas increases in cytoplasmic cAMP did not. They went on to investigate the uniquely ciliary events required and showed that PKA-dependent phosphorylation of a key transcription factor involved in cystogenesis required IFT-dependent ciliary trafficking. Many current models suggest, with little evidence, that cAMP diffusing from cilia is critical for downstream responses to ciliary receptor engagement, and these findings provide evidence against this model.

This work provides exciting new insights into mechanisms of cilium to cytoplasm signaling and demonstrates that the organelle has the capacity to support multiple features of a signaling pathway that are independent of cytoplasmic events. (What happens in cilia, stays in cilia.) Given that polycystic kidney disease is among the most common monogenic inherited diseases in human, these finding will also be relevant to the clinical community. Below I outline a few minor concerns.

We thank the reviewer for the positive evaluation, and we are happy to address the few minor concerns that remained.

1. *Figure 1: The differences in cAMP between cilia vs cyto bPAC are very small, even though difference is statistically significant. Also, it was difficult to interpret the number of experiments and the number of cilia assessed? If understood correctly, the cilia-bPAC experiment was done 8 times, and from these 8 experiments the fluorescence was quantified from a total of 14 cilia? Given the centrality of this information to the thrust of this work, need to provide results from many more cilia in each group.*

We are sorry for the confusion. We have performed 8 independent experiments for cilia-bPAC (14 cilia in total) and 9 independent experiments for cyto-bPAC (26 cilia in total). Given the complicated set-up that combines high-speed imaging with optogenetic stimulation, this is quite a high number already. Other reports using similar biosensors in cilia have used much lower numbers (<10 cilia in total from n = 3 experiments, e.g., Moore et al., PNAS 2016 or similar numbers Jiang et al., PNAS 2019). Also, our data is statistically significant and we provide the values and statistical analyses for the data in the original manuscript below in a table.

Table 1: Ciliary fluorescence intensities for Figure 1E. Each row indicates the ciliary fluorescence intensity in either cilia-bPAC or cyto-bPAC mIMCD-3 cells after photoactivation. Statistical test and p value is indicated.

	**Cilia-bPAC**	**Cyto-bPAC**
1	458.75	478.976094
2	722.89718	440.444445
3	579.937864	591.972691
4	481.069714	499.107033
5	477.722727	456.346296
6	604.131264	445.459993
7	479.02381	451.644613
8	491.383929	440.988889
9	539.652381	468.310326
10	880.75641	519.568015
11	1199.93985	819.854293
12	616.158927	774.920276

13	697.903704	490.678572
14	472.373016	470.502778
15		462.810504
16		469.32963
17		440.147222
18		426.375
19		598.046523
20		435.260648
21		653.084326
22		460.622863
23		468.366453
24		486.990278
25		478.040677
26		533.047222
Statistics (calculated in Graph Pad Prism, Version 9.3.1 (350), December 7, 2021) Kolmogorov-Smirnov test		
	p value	0.0378

Nevertheless, we followed the recommendation of the reviewer and have performed more experiments to increase the numbers for this experiment to n = 14 (cilia-bPAC, 40 cilia) and n = 12 (cyto-bPAC, 31 cilia) experiments. The outcome of the result remained the same (Fig. 1E).

2. *Figure 2: Authors need to comment on comparatively low cyst formation in the cyto-bPAC samples in Fig. 2E?*

In general, ciliary and cytoplasmic cAMP signaling seem to evoke opposite effects, as we have demonstrated already for the ciliary length control (Hansen et al., eLife 2020). Here, we show that this also applies to cyst development when increasing cAMP levels in the different compartments by optogenetic stimulation. However, bPAC also displays some dark activity, which might influence cellular signaling to some extent, even in the dark. In the dark, basal cAMP levels in cyto-bPAC cells are slightly increased compared to control cells (Fig. 1C). In turn, the Forskolin-dependent cyst formation in cyto-bPAC cells (Fig. 2E) seems to be reduced.

3. *MR-L8 experiments: Need reference. Also, did agent decrease ciliary cAMP in the cilia-bPAC, light-treated cells?*

MR-L8 is a further development of the compound MR-L2 described in Omar et al., PNAS 2019. MR-L8 is part of Mironid's published patent application WO2019193342, Example 34. We have added this information in the material and methods part.
To analyze whether activation of PDE4 long isoforms by MR-L8 reduced ciliary cAMP levels in cilia-bPAC cells upon photoactivation, we compared ciliary cAMP levels measured by cilia-Pink Flamindo after light stimulation in the presence or absence of MR-L8 (see Figure 4 below). The data shows a trend towards lower ciliary cAMP levels in the presence of MR-L8 but the differences are not statistically significant. This might be due to PDE4 long isoforms controlling specific signalosomes in the ciliary compartment without dramatically affecting ciliary cAMP levels in general. Furthermore, the high catalytic activity of bPAC might not be fully overcome by the activation of PDE4 long isoforms.

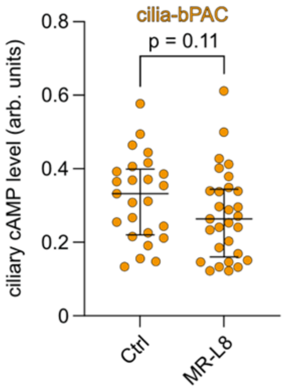

Figure 4: Measurements of ciliary cAMP levels using cilia-Pink Flamindo. mIMCD-3 cells expressing cilia-bPAC have been pre-treated with 10 µM MR-L8 or DMSO (ctrl) for 10 min before the measurement. Ciliary cAMP levels have been determined as the mean ciliary fluorescence intensity during the first 60 s after light stimulation (measurement interval 10 s); p value for an unpaired Student's t-test is indicated. Each data point represents an individual cilium (25-29 cilia from n = 6 experiments). Bars display median

and interquartile range, statistical comparison has been performed using a Mann-Whitney test, the p value has been indicated.

4. *Figure 5: In Jin, Ni 2014 paper, EP4 was present in cilia but also at the cell membrane. This was most obvious in the RPE1 cells (Jin fig7I, but also in the bottom-most IMCD3 cell in Fig7L). Need evidence that cytoplasmic cAMP did not increase in these experiments. Unless the authors document that EP4 is uniquely localized to cilia in these experiments, this conclusion is not warranted by the results.*

We agree with the reviewer and have performed further analyses to verify our results. First, we analyzed the subcellular localization of the EP4 receptor in mIMCD-3 cells. Indeed, the receptor was enriched in primary cilia in our cell line (Fig. 5A). We have shown in the original manuscript that PGE2 evokes an increase in ciliary cAMP levels (Fig. 5B-C), indicating that it evokes a response via the primary cilium. We have performed additional experiments using IFT20$^{-/-}$ mIMCD-3 cells to demonstrate that PGE2-dependent cyst formation relies on the presence of primary cilia (Fig. EV3D, E). Furthermore, MR-L8, which activates PDE4 long isoforms and blocks cyst formation induced by ciliary cAMP signaling (Fig. 4H, I), blocks PGE2-induced cyst formation (Fig. 5F). PGE2-dependent cystogenesis, like ciliary cAMP-induced cystogenesis, relies on downstream COX-2 action as we now demonstrate that celecoxib blocks PGE2-dependent cyst formation (Fig. 6I). Thus, there is compelling experimental evidence collected by us and others demonstrating that PGE2 induces cyst formation via primary cilia signaling. However, as the reviewer correctly pointed out, it is difficult to demonstrate that PGE2 exclusively functions via the primary cilium. And indeed, we have performed additional experiments, showing that PGE2 also increases total and cytoplasmic cAMP levels (Fig. EV3F-H), although significantly less than e.g., Forskolin. Nonetheless, considering all experiments presented here, our data strongly suggests that PGE2-dependent cyst formation predominantly relies on ciliary cAMP signaling. We have rephrased the text in the manuscript accordingly.

5. *Figure 6: Need to comment on possibility that Celecoxib specifically effects the cystogenic pathway as opposed to non-specific inhibition of cell proliferation.*

The reviewer correctly pointed out the celecoxib might also affect cell proliferation as 25 μM celecoxib has been shown to inhibit cell proliferation of cancer cells (Cao et al., Front. Pharmacol. 2020). To demonstrate the specificity of celecoxib in inhibiting ciliary cAMP-dependent cyst formation, we have performed additional experiments using 1 μM celecoxib, which still abolished cyst formation (Fig. 6G).

Referee #3:

The study by Hansen et al. investigates the role of ciliary cAMP in the regulation of cyst growth. The authors use the established murine inner medullary collecting duct cell line IMCD3, which can be used to study 3D cyst formation. By using an elegant optogenetic approach, they are able to induce cAMP formation inside the primary cilium by light. Interestingly, this affects cyst growth and proliferation, while cytoplasmic expression of the same p.a. enzyme does not. By RNAseq, the authors analyze transcriptional changes upon the ciliary cAMP increase and identify the CREB TF. Phospho CREB occurs in the cilium upon cAMP induction.

This is a fascinating manuscript and an important study on a very relevant topic of cilia biology. It is very well written. I really like the story, but I have a number of concerns that should be addressed by the authors.

We thank the reviewer for pointing out the importance of our data. We have tried to address all issues raised by the reviewers and hope that no further concerns remain.

Major points:

1. *The authors surprisingly show that ciliary bPAC but not cyto-bPAC results in cyst formation/cyst growth. This is exciting and, for me, the key finding of this study! However, I am not entirely convinced that cytobPAC, with its diffuse and non-targeted expression pattern, is the appropriate control here. Given the importance of this result for the entire study, I would suggest using additional controls with bPAC.mCherry targeted to the plasma membrane, the ER, or the outer mitochondrial membrane. Perhaps high local concentrations at one specific spot are necessary to exert an effect.*

As the reviewer pointed out, cyto-bPAC localizes to the entire cytoplasm without showing a distinct localization pattern. However, the increase in total cAMP levels evoked by photoactivation of cyto-bPAC is as high as the cAMP levels achieved by a saturating concentration of Forskolin (Fig. 1C). Nevertheless, only Forskolin, due to its combined ciliary action, promotes cyst formation whereas cyto-bPAC does not.
Our results further indicated that the activity of PDE4 long isoforms seems to be important to maintain the ciliary cAMP compartment by hydrolyzing cytosolic cAMP that may enter the cilium. To address the reviewer's comment, we blocked PDE4 activity in light-stimulated cyto-bPAC cells using rolipram, hypothesizing that abolishes the functional separation between the ciliary cAMP compartment and the cytoplasm, and analyzed cyst formation. In fact, inhibition of PDE4 activity caused cyst formation (Fig. 4 C, D) and increased ciliary cAMP levels (Fig. 4E), demonstrating that the PDE4 activity is crucial to functionally separate the ciliary cAMP compartment from the cytoplasm and this separation is inhibited, cytosolic cAMP entering the cilium can evoke a ciliary cAMP response. Furthermore, this also stresses again that the cAMP level produced by cyto-bPAC per se is sufficiently high to evoke cyst formation.

2. *The authors suggest that the Forskolin effect in Fig. 1 primarily results from ciliary cAMP increase. It would be good to demonstrate that Forskolin indeed enhances ciliary cAMP levels (e.g., by a cilia-Flamindo assay).*

This is a valid point and we have performed the required experiment. We now show that Forskolin increases ciliary cAMP levels (Fig. EV3A-B).

3. *Where does cilia-bPAC localize in non-ciliated, mitotic cells? Is it active? This could be relevant since the cyst formation-assay takes several days.*

We have previously analyzed that cilia-bPAC in non-ciliated HEK293 is active (Hansen et al., *eLife* 2020). However, this only applies to cells showing high cilia-bPAC expression levels. In general, under the expression conditions we used in this study, cilia-bPAC levels in cycling cells are very low (see Figure 5 below) and, in turn, the activity considerably low, indicating that cilia-bPAC in non-ciliated, dividing cells does not have a major impact on cellular signaling. In fact, we have used cyto-bPAC as a control to rule out that photoactivation of bPAC in the cytoplasm evokes cyst formation. As shown in the original manuscript, light stimulation of cyto-bPAC did not trigger cyst formation.

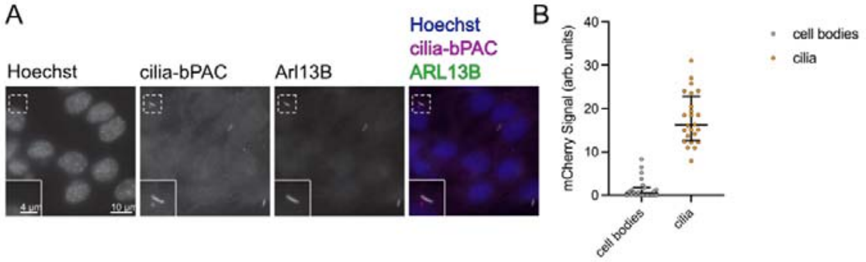

Figure 5: Cilia-bPAC localizes to cilia in cycling cells. (A) Immunocytochemistry of mIMCD-3 cell line stably expressing cilia-bPAC-mCherry. bPAC is visualized by mCherry fluorescence, nuclei were stained with Hoechst dye (blue), and cilia were visualized by an ARL13B-specific antibody (green, ciliary marker). Scale bars are indicated. Note that most cells in the micrograph are not ciliated show no appreciable bPAC signals. **(B)** Quantification of mCherry signal intensities in cell bodies and cilia in unsynchronized cilia-bPAC cells. Each data point represents the background subtracted average signal intensity in cilia or cell bodies, as indicated, from individual images (n = 24). Horizontal lines indicate medians, whiskers indicate interquartile ranges.

4. *With respect to the PDEs and EP4, it is unclear if these proteins are localized exclusively to primary cilia. They might occur at the apical or basolateral membrane and exert cilia-independent effects.*

This point is well taken and we have analyzed the localization of the EP4 receptor and the different PDE4 long isoforms in mIMCD-3 cells. Using immunofluorescence analysis, we demonstrate that the EP4 receptor is localized to primary cilia (Fig. 5A). Furthermore, we also demonstrate that the PDE4 long isoforms PDE4A-D are also localized to primary cilia (Fig. 4G). However, as the reviewer correctly pointed out, it is difficult to demonstrate exclusive localization in the primary cilium. Thus, we have rephrased the text in the manuscript accordingly.

5. *While it is striking to find CREB in the cilium and to speculate that CREB might shuttle back and forth from the cilium to the nucleus, this is actually not demonstrated by this study.*

Our data indicate that pCREB is transported in and out of the cilium as it is barely detectable in the cilium under steady-state conditions but accumulates after blocking retrograde transport when increasing ciliary cAMP levels. Although not formally demonstrated, this strongly suggests that indeed pCREB indeed seems to be shuttling through the cilium. Nevertheless, we have removed any strong statements in this direction.

Minor issues:

1. *The exact timing of the experiments remains unclear. E.g., in Fig 2 C/D: How long were cell treated with Forskolin? Is the 1h on/off cycling required for the observed effects? What is the reason for the cycling?*

We apologize for the lack of details in the description of the experiment. The cells were treated with Forskolin for 9 days with a fresh medium change every 2-3 days. The cycling between on and off phases with respect to light stimulation is necessary as continuous stimulation with blue light can be toxic to the cells.

2. *Why did the authors switch to wt control cells in Fig 3? It might be important to know whether cytoplasmic cAMP would also affect proliferation and mTOR (even without promoting cyst formation/growth).*

We agree with the reviewer and have performed additional experiments using cyto-bPAC cells. Photoactivation of cyto-bPAC cells did neither significantly increase cell proliferation (Fig. 3B) nor pS6RP levels (Fig. 3E).

3. *While it is not surprising that rapamycin reduces pS6RP, it would be essential to see whether rapamycin affects proliferation induced by cilia bPAC (as shown by the KI67 increase in Fig 3 A_C). If the authors aim to conclude that "ciliary cAMP engages mTOR signaling to promote cell proliferation", this should be demonstrated by rapamycin treatment of the cultures presented in Fig. 2.*

We thank the reviewer for this comment and we have performed the experiments accordingly. Proliferation induced by photoactivation of cilia-bPAC was significantly reduced in the presence of rapamycin (Fig. 3F).

4. *Full blots should be provided for Fig 4D.*

We have included the full blots for the Figures showing Western blot analysis (see Figure 6 below). However, when analyzing the ciliary PDE4 localization, we noticed that PDE4A was also slightly detectable in IMCD-3 cells. Thus, we repeated the Western blot analysis using a higher protein amount (60 µg). Thus, we show in the revised figure (Fig. 3F) the new blot and the respective full blot image in the Supplemental data (Appendix Fig. S2). When loading higher amounts of total protein, we could clearly detect PDE4A-D long and short isoforms. We now also included a loading control.

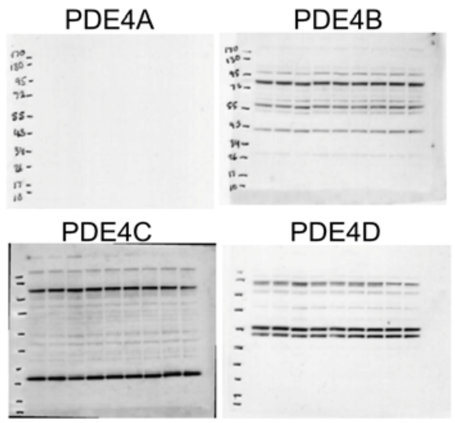

Figure 6: Original immunoblots demonstrating PDE4 long isoform expression in mIMCD-3 cells. Antibodies for the different PDE4 long isoforms are indicated.

5. *The concentration of the COX2 inhibitor with 20 µM is quite high. The EC50 is about 40nM (?). It might not be a COX2 specific effect. Can the authors reduce the concentration?*

We have followed the reviewer’s suggestion and reduce the celecoxib concentration to 1 μM. Still, cyst formation was reduced (Fig. 6G).

6. *I would suggest being more cautious when linking this story to ADPKD, in particular at the end of the abstract or in the middle of the introduction. Btw: the abstract and introduction share the very same last sentence.*
   The result section starts with "To investigate the role of ciliary cAMP signaling in kidney cyst growth, we used mIMCD-3 ....". It should become clearer throughout the MS, that's this IMCD assay is a somehow accepted 3D cell model but also quite limited in terms of "kidney cyst growth." The latter can only be studied in vivo.

We have changed the text accordingly.

1st Revision - Editorial Decision 9th May 2022

Dear Dagmar,

Thank you for submitting your revised manuscript. It has now been seen by all of the original referees.

As you can see, the referees find that the study is significantly improved during revision and recommend publication. However, I need you to address the points below before I can accept the manuscript.

• Referee #1 has remaining concerns and he/she requests some additional experiments to address them. I think adding these experiments would strengthen the manuscript. Please let me know if you would like to discuss any of the points further.
• We note that you provided 7 keywords. However, we cannot accommodate more than 5 keywords due to technical reasons. Therefore, please remove 2 of the keywords.
• We updated our journal's competing interests policy in January 2022 and request authors to consider both actual and perceived competing interests. Please review the policy https://www.embopress.org/competing-interests and update your competing interests if necessary. Also, please rename the 'Conflict of Interests' section as 'Disclosure statement and competing interests'.
• Regarding the Author Contributions, we now use CRediT to specify the contributions of each author in the journal submission system. CRediT replaces the author contribution section. Please use the free text box to provide more detailed descriptions. See also guide to authors https://www.embopress.org/page/journal/14693178/authorguide#authorshipguidelines.
• As per our format requirements, in the reference list, citations should be listed in alphabetical order and then chronologically, with the authors' surnames and initials inverted; where there are more than 10 authors on a paper, 10 will be listed, followed by 'et al.'. Please see https://www.embopress.org/page/journal/14693178/authorguide#referencesformat
• We note that Fig EV1A&B and Appendix Table S2 are currently not called out in the text.
• Please convert Appendix Table S4 into Dataset EV1 and update the callouts accordingly. Please add its name and legend into the file.
• We note that the Appendix file has a Table of Contents, but there are no page numbers. We also note a typo in the name of "Appendix Table S3". Appendix Fig S2 name needs correcting on the legend.
• Please make the GSE182339 dataset publicly available and remove the reviewers' password from the manuscript.
• Please change the title 'Main Text' as 'Introduction'.
• Materials and Methods should follow after the Discussion section.

Thank you again for giving us to consider your manuscript for EMBO Reports, I look forward to your minor revision.

Kind regards,

Deniz

--
Deniz Senyilmaz Tiebe, PhD
Editor
EMBO Reports

Referee #1:

The authors have done a commendable job in revising the manuscript and responding to my and other reviewers' comments. The crux of the paper is the specificity of the ciliary pool of cAMP in causing downstream cystogenesis that the authors are trying to argue. I am aware of the significant amount of work that the authors have already done in this important paper. So, I am leaving up to the authors if they would consider doing the following experiments before final publication.

The authors now show that the PGE2 mediated cystogenesis is inhibited in Ift20 ko cells. This is an important result that says that PGE2 function is someway regulated by cilia. The authors nicely show cAMP in cilia and cytoplasm upon PGE2 treatment. So PGE2 mediated cAMP increase is in both compartments. It might be useful for the authors to measure cytoplasmic cAMP levels in Ift20 ko cells upon PGE2 treatment if possible and test if the increase is not impacted by cilia loss.

Further, I think it would be important to test if Forskolin treatment mediated cystogenesis is also similarly abrogated in Ift20 ko cells. This would address if cytoplasmic cAMP level increase by Forskolin still requires cilia for cystogenesis.

The authors also show that Rolipram treatment shows some increase in ciliary cAMP during photoactivation by cyto-bPAC (Fig 6E). This is also an interesting result. However, have the authors measured cytoplasmic cAMP levels upon Rolipram treatment? One interpretation of Rolipram function in increasing some cystogenesis in cyto-bPAC cells is an increase in cytoplasmic cAMP levels that can signal in causing cystogenesis.

The authors now show that Ift27 ko cells have reduced Ptgs2 expression. Is it possible to test if Ift27 ko also impact on light induced or Forskolin induced cystogenesis?

Referee #2:

This is a beautiful, thorough study documenting the unexpected finding that transit of the CREB transcription factor through the cilium regulates it activity on cell proliferation. The authors have done a solid job of responding to my concerns. Although not required for publication, the authors might consider a fuller discussion of the relative roles of CREB and mTOR in cystogenesis. Also, it would have been helpful if the authors provided more on their ideas about why cAMP increases in the cytoplasm fail to lead to CREB activation in the cytoplasm.

Referee #3:

The authors have resubmitted a significantly revised manuscript and addressed all of my concerns.

Dear Dr. Senyilmaz Tiebe,

We would like to thank you and the reviewers for the positive evaluation of our work and we are happy to address the last minor issues remaining.
Please find our answers in the point-by-point response below. The text that has been modified in the manuscript has been highlighted in red. We have also created a cover art. In case of acceptance of our paper, it would be great if you would consider it as a cover image.

We are looking forward to your reply.

Kind regards,

Dagmar Wachten

Minor issues:

a. Referee #1 has remaining concerns and he/she requests some additional experiments to address them. I think adding these experiments would strengthen the manuscript. Please let me know if you would like to discuss any of the points further.

As discussed via email, we already acquired data sets to address question two and three. You can find our answers below.
However, as already discussed, addressing point 1 and 4 is also from your point of view not crucial, so we have not performed these additional experiments.

b. We note that you provided 7 keywords. However, we cannot accommodate more than 5 keywords due to technical reasons. Therefore, please remove 2 of the keywords.

We have reduced the number of keywords accordingly.

c. We updated our journal's competing interests policy in January 2022 and request authors to consider both actual and perceived competing interests. Please review the policy https://www.embopress.org/competing-interests and update your competing interests if necessary. Also, please rename the 'Conflict of Interests' section as 'Disclosure statement and competing interests'.

We did not need to update our competing interests but have renamed the section accordingly.

d. Regarding the Author Contributions, we now use CRediT to specify the contributions of each author in the journal submission system. CRediT replaces the author contribution section. Please use the free text box to provide more detailed descriptions.

We have removed the author contribution section in the main text and have instead used CRediT in the journal submission system.

e. As per our format requirements, in the reference list, citations should be listed in alphabetical order and then chronologically, with the authors' surnames and initials inverted; where there are more than 10 authors on a paper, 10 will be listed, followed

by 'et al.'. Please see https://www.embopress.org/page/journal/14693178/authorguide#referencesformat

We have changed the reference list and citations accordingly.

f. We note that Fig EV1A&B and Appendix Table S2 are currently not called out in the text.

We now mention Fig. EV1A-B in the text. Appendix Table S2 was already referred to in the Material & Methods sections under qPCR.

g. Please convert Appendix Table S4 into Dataset EV1 and update the callouts accordingly. Please add its name and legend into the file.

We have converted the Table into a Dataset.

h. We note that the Appendix file has a Table of Contents, but there are no page numbers. We also note a typo in the name of "Appendix Table S3". Appendix Fig S2 name needs correcting on the legend.

We have changed the Appendix file accordingly.

i. Please make the GSE182339 dataset publicly available and remove the reviewers' password from the manuscript.

The dataset is publicly available and we have changed the text accordingly.

j. Please change the title 'Main Text' as 'Introduction'.

We have changed the text accordingly.

k. Materials and Methods should follow after the Discussion section.

We have changed the text accordingly.

Referee #1:

The authors have done a commendable job in revising the manuscript and responding to my and other reviewers' comments. The crux of the paper is the specificity of the ciliary pool of cAMP in causing downstream cystogenesis that the authors are trying to argue. I am aware of the significant amount of work that the authors have already done in this important paper. So, I am leaving up to the authors if they would consider doing the following experiments before final publication.

1. The authors now show that the PGE2 mediated cystogenesis is inhibited in Ift20 ko cells. This is an important result that says that PGE2 function is someway regulated by cilia. The authors nicely show cAMP in cilia and cytoplasm upon PGE2 treatment. So PGE2 mediated cAMP increase is in both compartments. It might be useful for the authors to measure cytoplasmic cAMP levels in Ift20 ko cells upon PGE2 treatment if possible and test if the increase is not impacted by cilia loss.

See our comment above.

2. Further, I think it would be important to test if Forskolin treatment mediated cystogenesis is

also similarly abrogated in Ift20 ko cells. This would address if cytoplasmic cAMP level increase by Forskolin still requires cilia for cystogenesis.

We fully agree with the reviewer and have already performed this important experiment. Indeed, Forskolin treatment did not induce cyst formation in *Ift20*$^{-/-}$ cells. We have now included this information into the revised version of the manuscript (Fig. EV3F, G) and changed the text accordingly.

3. The authors also show that Rolipram treatment shows some increase in ciliary cAMP during photoactivation by cyto-bPAC (Fig 6E). This is also an interesting result. However, have the authors measured cytoplasmic cAMP levels upon Rolipram treatment? One interpretation of Rolipram function in increasing some cystogenesis in cyto-bPAC cells is an increase in cytoplasmic cAMP levels that can signal in causing cystogenesis.

To address this question, we have included a treatment with IBMX in our cyst assay as this allows to delineate the effects of the compartments and PDE isoforms by differential comparison. Whereas Rolipram evokes cyst formation in wild-type mIMCD-3 cells as well as in cilia-bPAC and cyto-bPAC cells by inhibiting PDE4 activity, IBMX only induced cyst formation in wild-type and cilia-bPAC, but not in cyto-bPAC cells (Fig. 4D, Fig. EV2B-D). IMBX targets broadly PDEs and thus increases cytoplasmic as well as ciliary cAMP levels. However, in cyto-bPAC cells, when boosting cytosolic cAMP levels, IBMX treatment does not trigger cyst formation. This demonstrates that a strong cytosolic cAMP boost does not trigger cyst formation but rather prevents it. In contrast, Rolipram, which is specific for PDE4 isoforms (with the PDE4 long isoforms being enriched in primary cilia), evokes cyst formation as it increases in particular ciliary cAMP levels while the cytosolic cAMP surge after bPAC activation in the cytoplasm can still be limited by other PDEs. These experiments highlight that a cytoplasmic cAMP increase is not sufficient to cause cyst formation and rather impairs cyst formation.

4. The authors now show that Ift27 ko cells have reduced Ptgs2 expression. Is it possible to test if Ift27 ko also impact on light induced or Forskolin induced cystogenesis?

See our comment above.

Dear Dagmar,

Thank you for submitting your revised manuscript. I have now looked at everything and all is fine. Therefore, I am very pleased to accept your manuscript for publication in EMBO Reports.

Congratulations on a nice work!

Before we can transfer your manuscript to our production team, I need your input on one more point. I recommend the following minor change in the title to make it fit better to our format:

A cAMP signalosome in primary cilia drives gene expression and kidney cyst formation

Please let me know what you think.

Kind regards,

Deniz
--
Deniz Senyilmaz Tiebe, PhD
Editor
EMBO Reports

--
Please note that you will be contacted by Wiley Author Services to complete licensing and payment information. The required 'Page Charges Authorization Form' is available here: https://www.embopress.org/pb-assets/embo-site/er_apc.pdf
--

At the end of this email I include important information about how to proceed. Please ensure that you take the time to read the information and complete and return the necessary forms to allow us to publish your manuscript as quickly as possible.

As part of the EMBO publication's Transparent Editorial Process, EMBO reports publishes online a Review Process File to accompany accepted manuscripts. As you are aware, this File will be published in conjunction with your paper and will include the referee reports, your point-by-point response and all pertinent correspondence relating to the manuscript.

If you do NOT want this File to be published, please inform the editorial office within 2 days, if you have not done so already, otherwise the File will be published by default [contact: emboreports@embo.org]. If you do opt out, the Review Process File link will point to the following statement: "No Review Process File is available with this article, as the authors have chosen not to make the review process public in this case." Please note that the author checklist will still be published even if you opt out of the transparent process.

Thank you again for your contribution to EMBO reports and congratulations on a successful publication. Please consider us again in the future for your most exciting work.

****

THINGS TO DO NOW:

Please note that you will be contacted by Wiley Author Services to complete licensing and payment information. The required 'Page Charges Authorization Form' is available here: https://www.embopress.org/pb-assets/embo-site/er_apc.pdf

You will receive proofs by e-mail approximately 2-3 weeks after all relevant files have been sent to our Production Office; you should return your corrections within 2 days of receiving the proofs.

Please inform us if there is likely to be any difficulty in reaching you at the above address at that time. Failure to meet our deadlines may result in a delay of publication, or publication without your corrections.

All further communications concerning your paper should quote reference number EMBOR-2021-54315V3 and be addressed to emboreports@wiley.com.

Should you be planning a Press Release on your article, please get in contact with emboreports@wiley.com as early as possible, in order to coordinate publication and release dates.

EMBO Press Author Checklist

Corresponding Author Name: Dagmar Wachten	
Journal Submitted to: EMBO Reports	
Manuscript Number: EMBOR-2021-54315	

USEFUL LINKS FOR COMPLETING THIS FORM
The EMBO Journal - Author Guidelines
EMBO Reports - Author Guidelines
Molecular Systems Biology - Author Guidelines
EMBO Molecular Medicine - Author Guidelines

Reporting Checklist for Life Science Articles (updated January 2022)
This checklist is adapted from Materials Design Analysis Reporting (MDAR) Checklist for Authors. MDAR establishes a minimum set of requirements in transparent reporting in the life sciences (see Statement of Task: 10.31222/osf.io/9sm4x). Please follow the journal's guidelines in preparing your manuscript.
Please note that a copy of this checklist will be published alongside your article.

Abridged guidelines for figures
1. Data
The data shown in figures should satisfy the following conditions:
- the data were obtained and processed according to the field's best practice and are presented to reflect the results of the experiments in an accurate and unbiased manner.
- ideally, figure panels should include only measurements that are directly comparable to each other and obtained with the same assay.
- plots include clearly labeled error bars for independent experiments and sample sizes. Unless justified, error bars should not be shown for technical replicates.
- if n<5, the individual data points from each experiment should be plotted. Any statistical test employed should be justified.
- Source Data should be included to report the data underlying figures according to the guidelines set out in the authorship guidelines on Data Presentation.

2. Captions
Each figure caption should contain the following information, for each panel where they are relevant:
- a specification of the experimental system investigated (eg cell line, species name).
- the assay(s) and method(s) used to carry out the reported observations and measurements.
- an explicit mention of the biological and chemical entity(ies) that are being measured.
- an explicit mention of the biological and chemical entity(ies) that are altered/varied/perturbed in a controlled manner.
- the exact sample size (n) for each experimental group/condition, given as a number, not a range;
- a description of the sample collection allowing the reader to understand whether the samples represent technical or biological replicates (including how many animals, litters, cultures, etc.).
- a statement of how many times the experiment shown was independently replicated in the laboratory.
- definitions of statistical methods and measures:
  - common tests, such as t-test (please specify whether paired vs. unpaired), simple χ2 tests, Wilcoxon and Mann-Whitney tests, can be unambiguously identified by name only, but more complex techniques should be described in the methods section;
  - are tests one-sided or two-sided?
  - are there adjustments for multiple comparisons?
  - exact statistical test results, e.g., P values = x but not P values < x;
  - definition of 'center values' as median or average;
  - definition of error bars as s.d. or s.e.m.

Please complete ALL of the questions below.
Select "Not Applicable" only when the requested information is not relevant for your study.

Materials

Newly Created Materials	Information included in the manuscript?	In which section is the information available? (Reagents and Tools Table, Materials and Methods, Figures, Data Availability Section)
New materials and reagents need to be available; do any restrictions apply?	Yes	Data Availability Section

Antibodies	Information included in the manuscript?	In which section is the information available? (Reagents and Tools Table, Materials and Methods, Figures, Data Availability Section)
For **antibodies** provide the following information:<br>- Commercial antibodies: RRID (if possible) or supplier name, catalogue number and or/clone number<br>- Non-commercial: RRID or citation	Yes	Materials and Methods

DNA and RNA sequences	Information included in the manuscript?	In which section is the information available? (Reagents and Tools Table, Materials and Methods, Figures, Data Availability Section)
**Short novel DNA or RNA including primers, probes**: provide the sequences.	Not Applicable	

Cell materials	Information included in the manuscript?	In which section is the information available? (Reagents and Tools Table, Materials and Methods, Figures, Data Availability Section)
**Cell lines:** Provide species information, strain. Provide accession number in repository **OR** supplier name, catalog number, clone number, and/**OR** RRID.	Yes	Materials and Methods
**Primary cultures:** Provide species, strain, sex of origin, genetic modification status.	Not Applicable	
Report if the cell lines were recently **authenticated** (e.g., by STR profiling) and tested for mycoplasma contamination.	Yes	Materials and Methods

Experimental animals	Information included in the manuscript?	In which section is the information available? (Reagents and Tools Table, Materials and Methods, Figures, Data Availability Section)
**Laboratory animals or Model organisms:** Provide species, strain, sex, age, genetic modification status. Provide accession number in repository **OR** supplier name, catalog number, clone number, **OR** RRID.	Not Applicable	
**Animal observed in or captured from the field:** Provide species, sex, and age where possible.	Not Applicable	
Please detail **housing and husbandry conditions**.	Not Applicable	

Plants and microbes	Information included in the manuscript?	In which section is the information available? (Reagents and Tools Table, Materials and Methods, Figures, Data Availability Section)
**Plants:** provide species and strain, ecotype and cultivar where relevant, unique accession number if available, and source (including location for collected wild specimens).	Not Applicable	
**Microbes:** provide species and strain, unique accession number if available, and source.	Not Applicable	

Human research participants	Information included in the manuscript?	In which section is the information available? (Reagents and Tools Table, Materials and Methods, Figures, Data Availability Section)
If collected and within the bounds of privacy constraints report on age, sex and gender or ethnicity for all study participants.	Not Applicable	

Core facilities	Information included in the manuscript?	In which section is the information available? (Reagents and Tools Table, Materials and Methods, Figures, Data Availability Section)
If your work benefited from core facilities, was their service mentioned in the acknowledgments section?	Yes	Materials and Methods, Ackknowledgements

Design

**Study protocol**	**Information included in the manuscript?**	**In which section is the information available?** (Reagents and Tools Table, Materials and Methods, Figures, Data Availability Section)
If study protocol has been **pre-registered, provide DOI in the manuscript**. For clinical trials, provide the trial registration number **OR** cite DOI.	Not Applicable	
Report the **clinical trial registration number** (at ClinicalTrials.gov or equivalent), where applicable.	Not Applicable	

**Laboratory protocol**	**Information included in the manuscript?**	**In which section is the information available?** (Reagents and Tools Table, Materials and Methods, Figures, Data Availability Section)
Provide DOI OR other citation details if **external detailed step-by-step protocols** are available.	Not Applicable	

**Experimental study design and statistics**	**Information included in the manuscript?**	**In which section is the information available?** (Reagents and Tools Table, Materials and Methods, Figures, Data Availability Section)
Include a statement about **sample size** estimate even if no statistical methods were used.	Yes	Figures and Figure legends
Were any steps taken to minimize the effects of subjective bias when allocating animals/samples to treatment (e.g. **randomization procedure**)? If yes, have they been described?	Not Applicable	
Include a statement about **blinding** even if no blinding was done.	Yes	Materials and methods.
Describe **inclusion/exclusion criteria** if samples or animals were excluded from the analysis. Were the criteria pre-established?<br><br>If sample or data points were omitted from analysis, report if this was due to attrition or intentional exclusion and provide justification.	Not Applicable	
For every figure, are **statistical tests** justified as appropriate? Do the data meet the assumptions of the tests (e.g., normal distribution)? Describe any methods used to assess it. Is there an estimate of variation within each group of data? Is the variance similar between the groups that are being statistically compared?	Yes	Figure legend

**Sample definition and in-laboratory replication**	**Information included in the manuscript?**	**In which section is the information available?** (Reagents and Tools Table, Materials and Methods, Figures, Data Availability Section)
In the figure legends: state number of times the experiment was **replicated** in laboratory.	Yes	Figure legend
In the figure legends: define whether data describe **technical or biological replicates**.	Yes	Figure legend

Ethics

**Ethics**	**Information included in the manuscript?**	**In which section is the information available?** (Reagents and Tools Table, Materials and Methods, Figures, Data Availability Section)
Studies involving **human participants**: State details of **authority granting ethics approval** (IRB or equivalent committee(s), provide reference number for approval.	Not Applicable	
Studies involving **human participants**: Include a statement confirming that **informed consent** was obtained from all subjects and that the experiments conformed to the principles set out in the WMA Declaration of Helsinki and the Department of Health and Human Services Belmont Report.	Not Applicable	
Studies involving **human participants:** For publication of **patient photos**, include a statement confirming that consent to publish was obtained.	Not Applicable	
Studies involving experimental **animals**: State details of **authority granting ethics approva**l (IRB or equivalent committee(s), provide reference number for approval. Include a statement of compliance with ethical regulations.	Not Applicable	
Studies involving **specimen and field samples:** State if relevant **permits** obtained, provide details of authority approving study; if none were required, explain why.	Not Applicable	

**Dual Use Research of Concern (DURC)**	**Information included in the manuscript?**	**In which section is the information available?** (Reagents and Tools Table, Materials and Methods, Figures, Data Availability Section)
Could your study fall under dual use research restrictions? Please check biosecurity documents and list of **select agents and toxins** (CDC): https://www.selectagents.gov/sat/list.htm	Not Applicable	
If you used a select agent, is the security level of the lab appropriate and reported in the manuscript?	Not Applicable	
If a study is subject to dual use research of concern regulations, is the name of the **authority granting approval and reference number** for the regulatory approval provided in the manuscript?	Not Applicable	

Reporting

The MDAR framework recommends adoption of discipline-specific guidelines, established and endorsed through community initiatives. Journals have their own policy about requiring specific guidelines and recommendations to complement MDAR.

**Adherence to community standards**	**Information included in the manuscript?**	**In which section is the information available?** (Reagents and Tools Table, Materials and Methods, Figures, Data Availability Section)
State if relevant guidelines or checklists (e.g., **ICMJE, MIBBI, ARRIVE, PRISMA**) have been followed or provided.	Not Applicable	
For **tumor marker prognostic studies,** we recommend that you follow the **REMARK** reporting guidelines (see link list at top right). See author guidelines, under 'Reporting Guidelines'. Please confirm you have followed these guidelines.	Not Applicable	
For **phase II and III randomized controlled trials**, please refer to the **CONSORT** flow diagram (see link list at top right) and submit the CONSORT checklist (see link list at top right) with your submission. See author guidelines, under 'Reporting Guidelines'. Please confirm you have submitted this list.	Not Applicable	

Data Availability

**Data availability**	**Information included in the manuscript?**	**In which section is the information available?** (Reagents and Tools Table, Materials and Methods, Figures, Data Availability Section)
Have **primary datasets** been deposited according to the journal's guidelines (see 'Data Deposition' section) and the respective accession numbers provided in the Data Availability Section?	Yes	Data availability section
Were **human clinical and genomic datasets** deposited in a public access-controlled repository in accordance to ethical obligations to the patients and to the applicable consent agreement?	Not Applicable	
Are **computational models** that are central and integral to a study available without restrictions in a machine-readable form? Were the relevant accession numbers or links provided?	Not Applicable	
If publicly available data were reused, provide the respective **data citations in the reference list**.	Not Applicable	
